# A model for the evolution of prokaryotic DNA restriction-modification systems based upon the structural malleability of Type I restriction-modification enzymes

**DOI:** 10.1093/nar/gky760

**Published:** 2018-08-28

**Authors:** Edward K M Bower, Laurie P Cooper, Gareth A Roberts, John H White, Yvette Luyten, Richard D Morgan, David T F Dryden

**Affiliations:** 1 EaStCHEM School of Chemistry, University of Edinburgh, The King's Buildings, Edinburgh EH9 3FJ, UK; 2 New England Biolabs, 240 County Road, Ipswich, MA 01938-2723, USA; 3 Department of Biosciences, Durham University, South Road, Durham DH1 3LE, UK

## Abstract

Restriction Modification (RM) systems prevent the invasion of foreign genetic material into bacterial cells by restriction and protect the host's genetic material by methylation. They are therefore important in maintaining the integrity of the host genome. RM systems are currently classified into four types (I to IV) on the basis of differences in composition, target recognition, cofactors and the manner in which they cleave DNA. Comparing the structures of the different types, similarities can be observed suggesting an evolutionary link between these different types. This work describes the ‘deconstruction’ of a large Type I RM enzyme into forms structurally similar to smaller Type II RM enzymes in an effort to elucidate the pathway taken by Nature to form these different RM enzymes. Based upon the ability to engineer new enzymes from the Type I ‘scaffold’, an evolutionary pathway and the evolutionary pressures required to move along the pathway from Type I RM systems to Type II RM systems are proposed. Experiments to test the evolutionary model are discussed.

## INTRODUCTION

Prokaryotic restriction-modification (RM) systems provide a major defence against invading foreign DNA (1–4) and as such their genes are found in over 96% of bacterial genomes and over 99% of archaeal genomes (5,6). A typical RM system (7–12) includes a restriction endonuclease (REase), whose cleavage of DNA is triggered by the recognition of a specific DNA sequence on foreign DNA. The other constituent part of the RM system is a methyltransferase (MTase), whose action prevents cleavage of host DNA by methylating the target DNA sequence. Given their significant role in protecting the host cell, it is surprising that RM systems are not essential to prokaryotic life. As such, RM systems should be viewed as necessary for the survival of the population, and not the individual cell; RM activity is the main method to prevent the spread of foreign DNA in a population (1,3,11,13–16) although additional roles have been proposed (3).

In some cases, RM functions are carried out by separate REase and MTase enzymes. However, in many systems both of these activities are fulfilled by a multi-subunit protein or even a single polypeptide (7,17). Hence, the RM systems show great variety in protein structure and gene sequence. To date, there are three classes of RM systems (Types I to III) and one class operating only on methylated DNA and thus lacking the modification function while retaining the restriction function (Type IV). These Types are separated due to differences in composition, target recognition, cofactors and the manner in which they cleave DNA (18). The defining characteristic of Type II RM systems, and perhaps the most important in terms of their use to molecular biology, is that their REase cleaves double stranded DNA at fixed, easily identified positions at or near to the target sequence (19).

### Type I RM enzymes and their structural malleability

**Figure 1.**
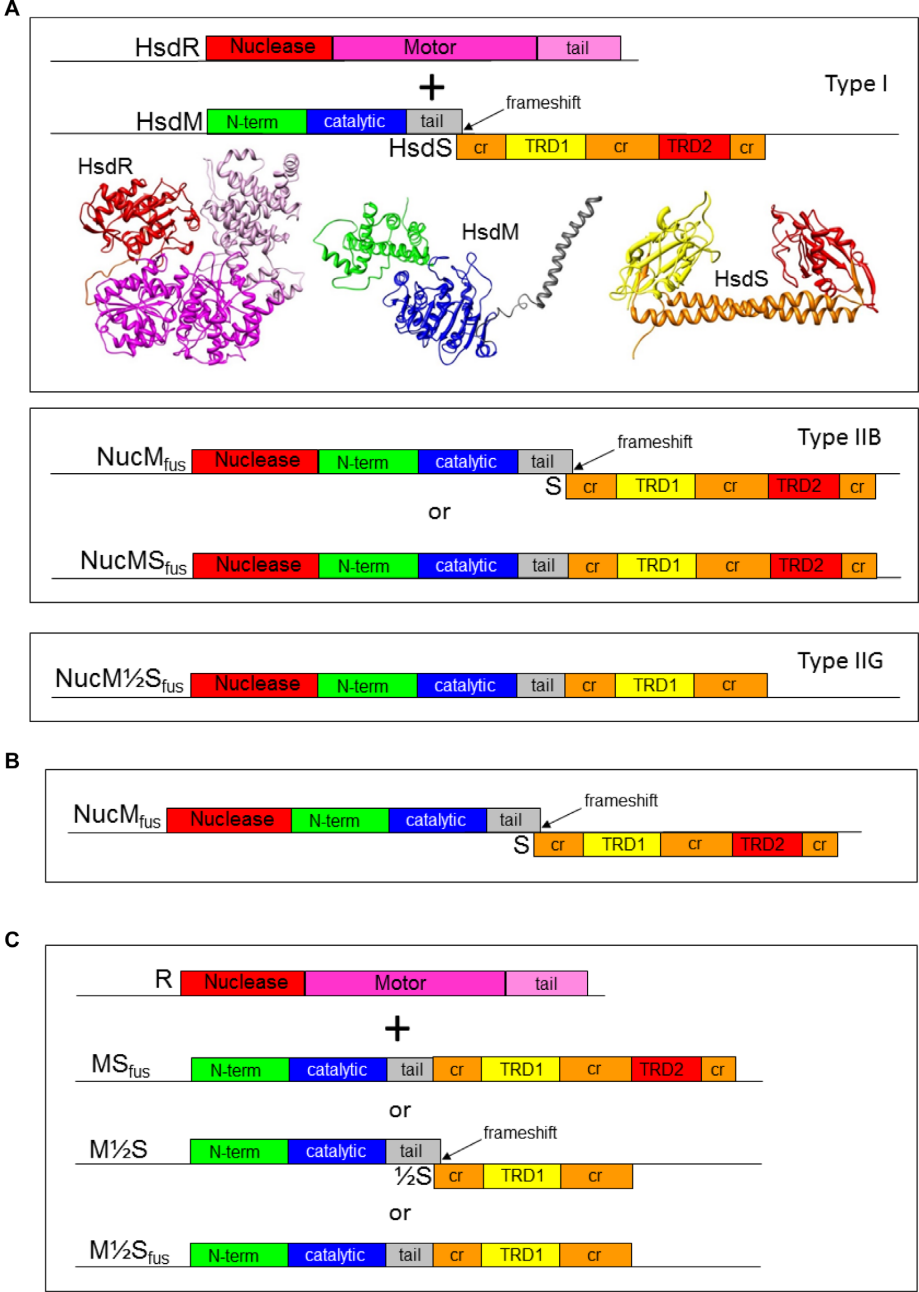
(**A**) A cartoon comparison of the domain structures encoded by Type I, Type IIB and IIG RM systems. The HsdR subunit contains three key domains, the N terminal nuclease (red), and the motor domain (dark pink) and tail region (light pink). The HsdM subunit contains three domains, the N terminal (green), the catalytic (blue), and a tail region (grey). The HsdS subunit is composed of two target recognition domains (TRDs, yellow and red), which are linked by regions of conserved sequence (‘CR’, orange). Structures derived by X-ray crystallography and modelling (24), are shown with the same domain-specific colouring (not to scale). The same colour scheme is used for the domains in Type IIB and IIG RM systems. (**B**) In this work, the fusion of the Nuclease domain of HsdR to the N-terminus of the HsdM is investigated. (**C**) Further domain re-arrangements between the HsdM and HsdS are also investigated in this work.

Type I systems were the first RM systems to be discovered (7,8,19). They are large hetero-oligomeric complexes, which perform cleavage of DNA away from their recognition site, in an ATP-dependent reaction (8,20–24), Figure 1A. A Type I restriction enzyme is composed of three separate subunits. These subunits are denoted by Hsd (host specificity for DNA) R for the restriction subunit (∼130 kDa), M for the MTase subunit (∼60 kDa), and S for the sequence-recognition specificity subunit (∼50 kDa). The ∼440 kDa restriction complex has a R_2_M_2_S_1_ stoichiometry, whilst a M_2_S_1_ stoichiometry acts as a cognate MTase for the system. Type I enzymes use energy from ATP hydrolysis to translocate DNA. The HsdR subunit binds both ATP and Mg^2+^ in order to perform the complicated process involved in producing double strand breaks in unmethylated DNA. The Type I enzyme binds its recognition sequence and the motor domains in the HsdR reel the DNA in towards the enzyme and cutting occurs when two HsdR motors collide. This can occur at anything from 40 bp to many kb away from the recognition site but is generally about half way between one site and the next target site.

Specific DNA sequences are recognised by the HsdS subunit (22–27). For the most part, these sequences possess the same general organisation of three specific nucleotides followed by a variable spacer of five to eight non-specific nucleotides (N), and then a further three to four specific nucleotides. For example, the target sequence of SauSTORF499P from *Staphylococcus aureus* clonal complex CC398 is ACC(N)_5_RTGA (26). The S subunit contains two target recognition domains (TRDs), separated by a central domain, which is conserved in members of the same family (20,25–27). The N-terminal TRD is specific for the 5′ part of the bipartite DNA sequence, while the C-terminal TRD recognises the 3′ part. The central conserved domain serves to coordinate interactions with the other subunits and more importantly, to separate the TRDs to a defined distance matching the length of the non-specific DNA spacer in the target sequence (20). The presence of additional N terminal and C terminal conserved domains, which together match the sequence of the central conserved domain, indicated a circular arrangement of the structure (28–32) which was confirmed by crystallography (33–35).

Alterations to the TRDs, which are encoded by the *hsdS* gene, can establish a new DNA specificity. TRDs may be swapped, truncated, switched and the number of non-specific nucleotides can be increased by extending the conserved sequence separating the two TRDs to engineer new specificity (25–27,36–40). Not only do the *hsdM* and *hsdS* genes share the same promoter but their open reading frames overlap at the junction created by the end of *hsdM* and the start of *hsdS* (41). Hence, the subsequent translation is coupled and during translation, a jump is required to create the two separate polypeptides. Roberts *et al.* were able to remove this frameshift from the MTase genes of the EcoKI Type I enzyme to create a fusion of the M and S subunits (42). This protein product showed full RM activity *in vivo* and was also successfully over-expressed and purified. With the addition of stoichiometric amounts of EcoKI HsdM protein, the purified fusion formed an active restriction complex *in vitro*.

### Type II RM enzymes and their classification

The most well-known and commonly used Type II REases fall into the subcategory of Type IIP as they bind and cleave palindromic sequences. The Type IIP REases, such as EcoRI and BglI, are ‘Orthodox’ ∼60 kDa homodimeric complexes, which cleave within or next to their recognition sequence (12,18). The associated MTases are usually thought of as monomers of ∼30 kDa although at least some function as dimers (17,43,44). Not all of the Type II RM systems conform to the narrow definition of Type IIP systems, and so they are separated into other sub-categories (18), Figure 1A. The factors that differentiate the many Type II sub-types are: the nature of the recognition sequence, tertiary and quaternary structure, and the type of cut produced on DNA. Type IIB systems have a subunit organization and activity not unlike Type I systems although they lack the ATP-dependent DNA-translocating motors (45,46). They possess REase, MTase and two TRDs in one enzyme, and can methylate either symmetric or asymmetric sequences which are indistinguishable from the targets recognised by the Type I RM systems. They cleave DNA in a SAM-dependent reaction and do so either side of their recognition sequence resulting in the removal of a short fragment. In effect they are ‘motor-less’ Type I RM systems. Like many Type IIB systems, IIG REases also encode REase, MTase and a single TRD in a single polypeptide. They are effectively half of a Type IIB RM enzyme or half of a ‘motor-less’ Type I RM system (47–49), Figure 1A.

### RM enzymes with similarity to Type I RM enzymes

In addition to the Type IIB and IIG RM systems discussed above, additional naturally-occurring, evolutionary intermediates between Type I and II systems are known. The Type ISP family possess all the functions of a Type I enzyme including the ATP-dependent motor, within a single polypeptide (SP) (50–52). The single TRD in a Type ISP system recognises a 6–7 bp asymmetrical target. They perform ATP-dependent dsDNA translocation and cleavage, and SAM-dependent modification. Type III RM enzymes are ATP-dependent hetero-oligomeric enzymes possessing REase and MTase in a single complex with a ‘res’ REase subunit and a ‘mod’ MTase subunit containing a well-defined TRD (9,53). The motor domain in the res subunit facilitates diffusion of the enzyme on the DNA rather than the directed translocation driven by the motor domain in the Type I HsdR subunits but the domains have the same evolutionary origin (53). There are also RM systems known colloquially as ‘Type one and a half’ systems comprised of a Type IIP REase and a Type I MTase (7) and more recently the structure of a Type IIP REase with a striking similarity to a Type I HsdS subunit has been determined (54).

### Evolution of RM enzymes

This work aims to investigate the hypothesis that Type I, Type II and Type III RM systems are evolutionarily linked. By making step-wise alterations to the subunits of the SauSTORF499P CC398-1 Type I RM system from *S. aureus* (15,25–27), active enzymes with novel specificities have been successfully engineered. Soluble fusions of the nuclease domain from HsdR to HsdM, HsdM to HsdS and HsdM to half-HsdS were produced. These new protein structures are comparable to Type II RM systems.

This leads us to propose a structural model for the evolution of RM systems which attempts to answer the following questions:

1. When did RM first appear?
2. Why did it appear?
3. What did the first RM system look like?
4. Why did it subsequently evolve to form the large range of RM variants observed today?
5. What was the evolutionary pathway?

## MATERIALS AND METHODS

### Chemicals, bacterial strains and phage

All chemicals were purchased from Sigma-Aldrich unless otherwise stated.

Bacterial strain *Escherichia coli* NM1261 (*r_K_*^−^*m_K_*^+^) was used for assays for *in vivo* activity (27). Bacterial strains *E. coli* BL21 (DE3) (Δ*hsdS_B_*) and DH5α (hsdR17 *r_K_*^−^*m_K_*^+^) were from New England Biolabs (Ipswich, MA). Bacterial strain *E. coli* ER2796, a kind gift from Dr E. Raleigh (New England Biolabs), was used in the SMRT sequencing protocol (55). *E. coli* ER2796 is a derivative of *E. coli* K12 with the phenotype*λ-fhuA2 Δ(lacZ)r1 glnV44 mcr-62 trp-31 dcm-6 zed-501::Tn10 hisG1 argG6 rpsL104 dam-16::Kan xyl-7 mtlA2 metB1 (mcrB-hsd_K_-mrr)114::IS10* lacking all host DNA methylation systems (55).

The pJF118His plasmid for expression of all genes has been previously described (27). Recombinant plasmids were isolated from transformed *E. coli* DH5α cells and the desired DNA sequences were confirmed. The amino acid sequences of the HsdR, HsdM and HsdS used in this work are given in Supplementary Table S1.

Fusions of the DNA encoding the nuclease domain of HsdR to the 5′ end of *hsdM* were created using PCR. The first RM fusion gene was created in a PCR using the oligonucleotides, ‘Mu50nucuni TS’ (5′-AGTCAGTCAGGGATCCAAGAAGGAGATATACATATGGCATACCAAAGTGAATACGC-3′) and ‘Mu50nucendalpha-CC398-1BS’ (5′-CGTTGTTTTTCAGTAATAGACATATTATTCCCTGTCTCAGTCG-3′), with the template *hsdR* gene (SauN315ORF189P) previously ligated into pRSFDuet-1 (Novagen) (27). The second PCR was conducted using the oligonucleotides, ‘Mu50nucendcoil-CC398-1TS’ (5′-CGACTGAGACAGGGAATAATATGTCTATTACTGAAAAACAACG-3′) and ‘C398-1BS’ (5′-GATCGAATTCCGGATCCAATAAACATCTTTTGAAGTAATGAC-3′), with the wild-type CC398-1 MTase genes in pJF118His vector as template (27). Further *hsdR* to *hsdM* fusion constructs were created using the same outer primers (‘Mu50nucuni TS’ and ‘CC398-1BS’) and specific primers for the different regions of *hsdR*, to which the *hsdR* portion of the fusion would be truncated. The fusion constructs, the primers used to create them in PCR and their amino acid sequences are summarised in Supplementary Table S2. The fusion proteins retain the methionine encoded by the ATG codon at the start of *hsdM*. The gene sequences used as a source for this work are SauN315ORF189P, M.SauSTORF499P and S.SauSTORF499P from REBASE (6).

To create the CC398-1 MS fusion gene encoding the protein ‘MS_fus_’, the *hsdM* open reading frame was fused in frame to *hsdS* by the polymerase chain reaction (PCR) and the resulting product was ligated into the pJF118His vector. PCR using oligonucleotides ‘HsdM-TS’ (5′-GATCGATCGGATCCAAGAAGGAGATATACATATGTC-3′) and ‘MTasefusion-BS’ (5′-GCACATTTTTCTTTTGTGTATTACTCATCTCATCTTTCAACACCCCAAG-3′), with the wild-type CC398-1 MTase genes in pJF118His as template, generated a fragment comprising the 5′ UTR upstream of *hsdM* and the entire *hsdM* ORF, fused in frame with the first 28 bases of *hsdS*. A PCR with a second pair of oligonucleotides, ‘MTasefusion-TS’ (5′-CTTGGGGTGTTGAAAGATGAGATGAGTAATACACAAAAGAAAAATGTGC-3′) and ‘CC398-1BS’ (5′-GATCGAATTCCGGATCCAATAAACATCTTTTGAAGTAATGAC-3′), with the wild-type CC398-1 MTase genes in pJF118His as template, generated a fragment comprising the last 29 bases of *hsdM* fused to the entire ORF of *hsdS*. These two PCR products were purified and fused in a reaction primed with oligonucleotides ‘HsdM-TS’ and ‘CC398-1BS’. The resulting product was purified and digested with BamHI. pJFMS was digested with BamHI, treated with Calf Intestinal Phosphatase and then ligated with the PCR product.

To create the ‘Half S’ MTase ‘M$^{1}\!\!/_{\!2}$S’, *hsdS* was truncated at the end of the central conserved region, directly before the start of the second TRD (equivalent to amino acid D220). PCR was performed with primers ‘HsdM-TS’ and ‘CC398-1 TRD 1 BS2’ (5′-GATCGAATTCCGGATCCATCTTTACCATTCTCATCTTTAAATCG-3′) with wild-type CC398-1 MTase genes in pJF118His as template. The product of this reaction was subjected to agarose gel electrophoresis and the band of the expected size was excised, gel eluted, BamHI digested and then ligated into vector pJF118His. The *hsdM* to half *hsdS* fusion gene to produce the protein ‘M$^{1}\!\!/_{\!2}$S_fus_’ was made in the same way but with the fused MTase genes in pJF118His in the PCR.

The amino acid sequences of these constructs are given in Supplementary Table S3. The fusion proteins retain the methionine encoded by the ATG codon at the start of *hsdS*.

### Bacterial genome single molecule real-time (SMRT) sequencing

Non-methylating (*dam^−^ dcm^−^*) *E. coli* ER2791 competent cells were transformed with a plasmid containing the target MTase and spread on a plate of lysogeny broth (LB) agar (25). Agar plates were supplemented with 100 μg/ml carbenicillin, which acted as a selection marker for the expression construct. Plates were incubated at 37°C overnight. A colony of successful transformants was picked into 5 ml of LB supplemented with 100 μg/ml carbenicillin and incubated overnight at 37°C whilst shaking. Cells from the subsequent culture were separated into 1 ml aliquots and harvested by centrifugation at 2380 x *g* for 15 minutes at 4°C. The Wizard Genomic DNA Purification Kit (Promega, Madison, WI, USA) was then used to lyse the cells and purify the genomic DNA. The quality of the genomic DNA preparations was initially assessed by agarose gel electrophoresis and from the shape of the absorbance profile from 240 to 340 nm. The DNA library for SMRT sequencing was prepared and subsequently analysed using a Pacific Biosciences sequencer as described in Anton *et al.* (55).

### Gene expression and purification of proteins

Overexpression of all genes was carried out in *E. coli* BL21 (DE3) competent cells, which were transformed with the plasmid expressing the target gene. Induction of expression was performed by adding IPTG to 1 mM and further growth at 20°C overnight (∼18 h).

All proteins were expressed with a hexa-HisTag attached to the C-terminus of the HsdS part of the protein. After overexpression of target genes, the *E. coli* BL21 (DE3) cell pellets were resuspended in 20 mM sodium phosphate buffer with 500 mM NaCl (pH 7.5), 20 mM Imidazole and a dissolved EDTA-free protease inhibitor tablet (Roche), in a 1:10 (g: ml) ratio. The cells were then disrupted by sonication using a Soniprep 150 sonicator (Sanyo, Tokyo, Japan), fitted with a 9mm diameter probe for ∼20 minutes with intermittent cooling. Cells were then centrifuged at 7700 x *g* for ∼45 min at 4°C. The supernatant was filtered through a filter unit (0.45 um; Sartorius AG, Goettingen, Germany) and then applied to a pre-equilibrated Histrap FF 5 ml column (GE Healthcare) at a flow rate of 100 ml/hr. The flow-through was collected. The column was then washed with 100 ml 20 mM Imidazole buffer and the flow-through was collected. This was followed by an elution of the protein with ∼10 ml of buffer supplemented with 500 mM imidazole, discarding the first 3 ml and collecting the next 6 ml. This was then concentrated to ∼4 ml, using a 20 ml 30 000 MWCO Vivaspin concentrator (Sartorius).

The 500 mM imidazole-containing buffer was removed from the sample by buffer exchange. This was performed using a PD-10 desalting column (GE Healthcare). The protein sample was concentrated to 2.5 ml and loaded on to the PD-10 column equilibrated with 20 mM Tris–HCl pH 8, 10 mM MgCl_2_, 500 mM NaCl and 7 mM 2-mercaptoethanol buffer. After elution, sample concentration was determined by an *A*_280_ reading. Part of the sample was immediately used in an assay to determine presence of DNA cleavage activity and the remainder concentrated in the Vivaspin concentrator (Sartorius). Samples were stored at -20°C after the addition of glycerol to 50% (v/v).

All HPLC analytical size exclusion runs were carried out using a BioSep-SEC-S 3000 (Phenomenex) column and a pH 6.5 buffer (20 mM Tris, 20 mM MES, 10 mM MgCl_2_, 200 mM NaCl, 0.1 mM EDTA, 7 mM 2-mercaptoethanol). This buffer was used to dilute the samples to a concentration of approximately 4 μM, 50 μl of which were then injected onto the HPLC system for each run. A flow rate of 0.5 ml/min was used for each run, which took approximately 10 min to complete. The absorbance at 280 nm was monitored and recorded by a data logger. The column was calibrated using several protein standards (Sigma Aldrich) of various concentrations, and a calibration curve was produced.

### Assessment of RM activity in vivo

The methods employed for assaying RM activity *in vivo* used the efficiency of plating (eop) of phage λ_v_ prepared from either a strain lacking the MTase genes to obtain unmodified phage or a strain transformed with the MTase plasmid to obtain modified phage have been previously described (56,57). All assays were performed in triplicate either as spot tests (nuclease domain fusions) or as full plate tests (MS fusions). The spot tests are suitable when restriction is absent and the whole plate tests are required when restriction is present to better quantify the degree of restriction. The spot tests used for assaying the nuclease fusions often give a standard deviation of ∼30% so a value of eop greater than one can occur (56). The promoter on the expression plasmids is slightly leaky so IPTG was not required to be added to the plates.

### In vitro DNA cleavage assay

Assays were conducted by incubating the enzyme under investigation with a library of plasmids. These plasmids were created by the ligation of known DNA sequences between EcoRI and BamHI sites of pUC19. The plasmids in the library were based on the DNA sequence of phage PhiED1 (a gift from Garry Blakely, University of Edinburgh). These plasmids are numbered sequentially from 1E to 20E (omitting 3E and 8E). The plasmid and specific insert sequences of these plasmids have been described previously (27) and are given again in the Supplementary Table S4. Each plasmid contains a ∼2.4 kb insert that was PCR amplified from *Bacteroides fragilis* phage PhiED1, ligated into vector pUC19. Collectively, the 18 plasmids contain >40 kb of known sequence and comparing their susceptibility to cleavage allows inference of REase specificity (27). MTases under investigation were supplemented with R subunit from *S. aureus* CC5 and incubated with the plasmid library in separate reactions. Reaction digests had a total volume of 50 μl and a typical digest was prepared using 5 μl of 10× NEBuffer 4 [New England Biolabs; 50 mM potassium acetate, 20 mM Tris–acetate, 10 mM magnesium acetate, 1 mM dithiothreitol (pH 7.9)], 2 mM ATP, 0.64 mM *S*-adenosyl-l-methionine, 0.01 mg of bovine serum albumin and 10 μl of the enzyme stock. The enzyme stock was prepared in a volume of 50 μl with 5 μl of 10× NEBuffer 4 with final concentrations of 1.16 μM R subunit and 0.42 μM MTase, thus ensuring an excess of R over the MTase to give formation of the R_2_M_2_S_1_ RM enzyme. Incubations were left for 12 min in a water bath set at 37°C. The reactions were stopped by the addition of Proteinase K (Roche) and incubated in a 60°C water bath for 25 min. Samples were then analyzed by agarose gel electrophoresis. Cleavage sites are distant from the target site for these enzymes; therefore, a computer program, RMsearch, was used to search for target sequences present in plasmids cut by the enzyme and not present in uncut plasmids (58).

## RESULTS

The four variants of the CC398-1 SauSTORF499P Type I RM system shown in Figure 1B and C were constructed. The nuclease fusion had ten subvariants with differing lengths of the 5′ end of *hsdR* fused to the 5′ end of *hsdM* as shown in Supplementary Table S2. These different lengths were chosen by comparison of the sequence of HsdR with the known sequence and structure of the HsdR protein from the EcoR124I RM system (59,60), Supplementary Figure S1. This process was informed by secondary sequence predictions and a protein model, created by the Phyre^2^ online software (61).

Two subvariants of the M$^{1}\!\!/_{\!2}$S protein were engineered with the full length *hsdM* followed by the first 666bp or 639bp of *hsdS*. The longer sequence from *hsdS* encodes all of the central conserved region of HsdS. The M$^{1}\!\!/_{\!2}$S_fus_ construct contained the first 666bp of *hsdS* fused in frame to *hsdM*.

### In vivo analysis of restriction and modification activity using phage λ

The *in vivo* assay detecting restriction and modification of λ phage is a simple way to assess the activity of the variants of the CC398-1 RM system. Efficiency of plating (eop) of phage λ is the ratio of phage titre on a restricting strain versus a non-restricting strain: in this case a strain transformed with two plasmids, one for HsdR and one for the MTase, versus the same strain transformed with the MTase plasmid. The strain containing the ‘wild-type’ CC398-1 MTase plus the HsdR showed nearly a ten-fold reduction in eop and is therefore active in restriction, Table 1.

**Table 1.** The *in vivo* restriction and modification activity of the wild type CC398-1 Type I RM system and its derivatives assessed by the efficiency of plating (eop) of phage λ_v_ on *E. coli* strain NM1261 transformed with plasmids expressing CC398-1 (HsdR + HsdM + HsdS) or its derivatives (first column). NM1261 has no RM activity overlapping with RM systems in this investigation. The first column also shows the length of the nucleotide sequences taken from the 5′ end of the *hsdR* gene for fusion to the 5′ end of the *hsdM* gene, and the equivalent number of amino acids (aa), to make the RM_EB_X series of fusions. Two versions of the M$^{1}\!\!/_{\!2}$S construct were tested. The second column shows the eop of phage infecting the strain transformed with the RM system relative to the strain cotransformed with MTase and pRSFDuet-1 (the vector used for supplying *hsdR*). A low eop (∼0.1) indicates restriction proficiency. The third column shows the eop of phage recovered from the experiments in the second column on reinfection of strains transformed with the RM system relative to the strain cotransformed with MTase and pRSFDuet-1. A high eop (∼1) indicates modification proficiency

RM system	Eop of phage λ_v_ on *E. coli* NM1261 expressing the system	Eop of recovered phage λ_v_ on *E. coli* NM1261 expressing the system
HsdR + HsdM + HsdS	0.15	0.88
HsdS + RM_EB_2 (1–786, aa 1–262)	1.1	1.67
HsdS + RM_EB_3 (1–651, aa 1–217)	0.68	0.8
HsdS + RM_EB_4 (1–621, aa 1–207)	1.5	1.13
HsdS + RM_EB_5 (1–645, aa 1–215)	2.25	1.06
HsdS + RM_EB_6 (1–675, aa 1–225)	1.0	0.67
HsdS + RM_EB_7 (1–732, aa 1–244)	2.0	1.0
HsdS + RM_EB_8 (1–513, aa 1–171)	0.75	0.89
HsdS + RM_EB_9 (1–549, aa 1–183)	1.25	0.8
HsdS + RM_EB_10 (1–816, aa 1–272)	0.75	0.59
HsdS + RM_EB_11 (1–855, aa 1–285)	0.5	1.25
HsdR + MS_fus_	0.09	0.78
HsdR + M$^{1}\!\!/_{\!2}$S (M$^{1}\!\!/_{\!2}$S shorter version)	0.58 (0.40)	0.75 (0.70)
HsdR + M$^{1}\!\!/_{\!2}$S_fus_	0.10	1.02

No significant change in eop from a value of one (values range from 0.5 to 2.25) was observed in strains expressing any of the ten nuclease-HsdM fusions along with HsdS, Table 1, indicating that none of these fusions possess restriction activity. However, phage recovered from these assays had all become modified as shown by the titre of these phage on the strain harbouring the wild-type CC398-1 MTase + HsdR compared to their titre on a strain lacking HsdR, Table 1. This indicates that these fusions are active in modification and have the same sequence specificity as the wild-type system. However as described in the next section, the protein instability noted during purification may also be present *in vivo* so the modification activity may be due to an assembly without the nuclease domain, a possibility that is difficult to rule out at present.

In the presence of HsdR, the M$^{1}\!\!/_{\!2}$S protein was only active in modification but not restriction, Table 1. This is in contrast to the situations found with other truncations of the S subunit of Type I RM systems (28,29,31). This may be due to protein instability as observed when purifying this protein as described below. In contrast, both MS_fus_ and M$^{1}\!\!/_{\!2}$S_fus_ in the presence of HsdR were active in both modification and restriction, Table 1. The apparent regaining of restriction activity when comparing M$^{1}\!\!/_{\!2}$S with M$^{1}\!\!/_{\!2}$S_fus_ is presumably due to an improved protein stability as discussed below as the amount of HsdR subunit being expressed in the cells should be the same.

### Protein expression

In our attempts to make a Type IIB RM enzyme, only one of the nuclease domain-HsdM fusions, RM_EB_2 protein, could be expressed and partially purified along with the S subunit, Supplementary Figure S2. SDS-PAGE of fractions from a size exclusion chromatography column indicated that the HsdS initially coeluted with a small amount of RM_EB_2 (lane 4 in Supplementary Figure S2) followed by coelution of HsdS with RM_EB_2 and a ∼70 kDa fragment (see lanes 6 to 8 in Supplementary Figure S2). This was then followed by coelution of HsdS with RM_EB_2 and a fragment of ∼61 kDa (see lanes 8 and 9 in Supplementary Figure S2) and lastly by coelution of HsdS with RM_EB_2 and a fragment of ∼65 kDa (see lane 10 in Supplementary Figure S2). As it is known that deletion of the C-terminus of the HsdM subunit prevents assembly with HsdS (62) then these fragments of RM_EB_2 must be deletions of the N-terminal region and their size indicates that these fragments have lost the nuclease domain. Thus, it appears that RM_EB_2 predominantly forms a complex of the form RM_EB_2 + truncated fragment + HsdS and this may explain the absence of nuclease activity *in vivo* as the complex mostly contains only a single nuclease domain.

The MS_fus_, M$^{1}\!\!/_{\!2}$S and M$^{1}\!\!/_{\!2}$S_fus_ fusions and truncations all expressed well and were soluble, Supplementary Figures S3, S4 and S5, respectively. Although it had a tendency to aggregate with time, the M$^{1}\!\!/_{\!2}$S_fus_ protein was the purest of these three proteins.

The MS_fus_ protein was unstable during the purification procedures and underwent some proteolysis. The additional bands on an SDS-PAGE gel were excised and sent for analysis by peptide fragmentation mass spectrometry at the Mass Spectrometry and Proteomics Facility (University of St. Andrews). Mass spectrometry results confirmed the larger of the contaminating species contained a C-terminally clipped form of the MS_fus_ species of ∼73 kDa and the smaller species was an unrelated *E. coli* protein (data not shown). The ∼73 kDa fragment comprises all of HsdM and about half of the first TRD of HsdS. A Sephacryl S200 size exclusion purification step was added to the purification method, however the larger fragment remained bound to the MS_fus_ suggesting that a proportion of the CC398-1 MS_fus_ protein is copurified with the clipped fragment.

Analysis of pooled protein fractions on a calibrated analytical size exclusion column showed a single major elution peak for CC398-1 MTase, M$^{1}\!\!/_{\!2}$S and M$^{1}\!\!/_{\!2}$S_fus_ proteins with apparent molecular masses of 241, 225 and 251 kDa respectively, Supplementary Figure S6 and Supplementary Table S5. The expected molecular masses for these proteins are 166 to 173 kDa assuming the forms M_2_S_1_, M_2_($^{1}\!\!/_{\!2}$S)_2_ and M($^{1}\!\!/_{\!2}$S_fus_)_2_ for these proteins. It has been previously noted that Type I MTases elute with this higher than expected molecular mass due to their highly non-spherical shape (22,63–69). The M$^{1}\!\!/_{\!2}$S and M$^{1}\!\!/_{\!2}$S_fus_ proteins also showed material eluting before the main protein peak indicating the presence of very high molecular mass aggregates. As noted above, the MS_fus_ protein co-eluted with a proteolytic fragment from the Sephacryl S200 column. The analytical column showed a complex asymmetric elution profile with the maximum UV absorption corresponding to a molecular mass of 325 kDa, Supplementary Figure S6. This higher mass may indicate that the fusion protein can also exist as a dimer (MS_fus_)_2_. The broadness of the elution profile may also suggest monomeric fusion protein and the existence of a complex equilibrium of different quaternary structures.

### Plasmid cleavage activity in vitro

**Figure 2.**
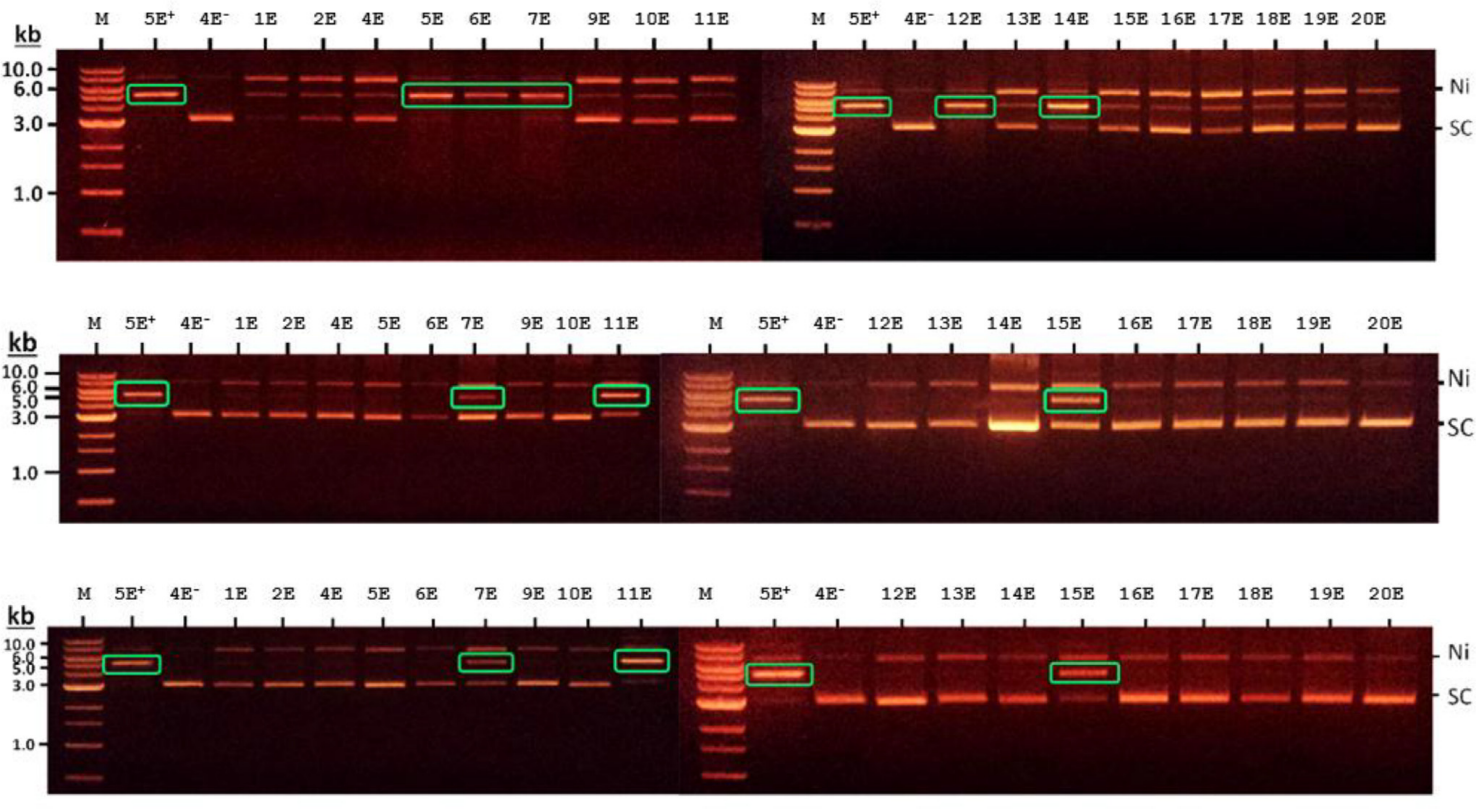
Agarose gel electrophoresis analysis of samples from a plasmid cleavage assay using the ‘E’ series of plasmids described previously (27). From top to bottom the gels show the effect of the purified HsdR incubated with MS_fus_, M$^{1}\!\!/_{\!2}$S and M$^{1}\!\!/_{\!2}$S_fus_ on the plasmid. The gels show three different plasmid DNA species. The species that occurs at the top of each gel lane has a single strand cut and is ‘nicked’ (Ni). Below this is the linearised species, when both strands have been cut by enzymatic activity (highlighted by green boxes). The lowest species is the uncleaved supercoiled DNA (SC). The lanes marked M are molecular size markers (kb). Lanes marked 5E^+^ and 4E^−^ are plasmids incubated with HsdR plus CC398-1 MTase that contain a single site or no site for the RE and act as positive and negative controls, respectively.

A set of plasmids containing inserts of known sequence was used for cleavage assays. Conversion of closed circle, supercoiled plasmid DNA to linear form was used as evidence of cleavage. As previously noted, the introduction of single-strand nicks into the plasmids to give an open circular form is attributed to the presence of nuclease contaminants in our enzyme preparations and is not evidence for the presence of a target site (27). In addition, the MS_fus_ enzyme produced a small amount of linearised DNA with every plasmid used, Figure 2 top row. However, in this case the absence or near absence of closed circular DNA in plasmids containing the target site was obvious and gave a target agreeing with the results of SMRT sequencing.

The partially purified complex of nuclease-HsdM fusion, RM_EB_2, and S subunit showed no cleavage activity when incubated with a plasmid containing a target site (data not shown). This is in agreement with the *in vivo* results shown in Table 1.

The MS_fus_, M$^{1}\!\!/_{\!2}$S and M$^{1}\!\!/_{\!2}$S_fus_ proteins were all active in cleavage when complemented with HsdR as shown in Figure 2. Of note is that extra HsdM was NOT required for MS_fus_ to be active. This is in contrast to the MS_fus_ protein constructed from the EcoKI Type I RM system which needed additional HsdM to be added to the reaction (42). This is consistent with the identification of a fragment of the fusion protein remaining associated with CC398-1 MS_fus_ throughout protein purification. RMsearch analysis showed recognition by MS_fus_ of the wild-type CC398-1 RM enzyme target of ACCN_6_TGA as anticipated (26). The $^{1}\!\!/_{\!2}$S proteins, M$^{1}\!\!/_{\!2}$S and M$^{1}\!\!/_{\!2}$S_fus_ recognise a different target, ACCN_5_GGT, which is palindromic and has a spacer change resulting in the adenine targets being one base pair further apart than for the wild type CC398-1 RM enzyme (9 bp rather than 8 bp) even though the overall target sequence is one base pair shorter (11 bp rather than 12 bp). The DNA cleavage activity of M$^{1}\!\!/_{\!2}$S_fus_ and M$^{1}\!\!/_{\!2}$S contrasts with the *in vivo* restriction activity, which was only clearly observed for M$^{1}\!\!/_{\!2}$S_fus_, suggesting an impaired association of M$^{1}\!\!/_{\!2}$S with HsdR or too low an amount of HsdR being expressed *in vivo*.

### Single-molecule real-time (SMRT) sequencing

SMRT sequencing is able to identify modified bases in sequenced DNA and can therefore be used to identify the DNA recognition motif of a Type I MTase (e.g. (25,26)). The method is particularly useful in that all modification is conducted *in vivo* eliminating the need for large amounts of purified protein.

The expression plasmids containing the wild-type CC398-1 MTase, MS_fus_, M$^{1}\!\!/_{\!2}$S and M$^{1}\!\!/_{\!2}$S_fus_ genes were used to transform competent *Escherichia coli* ER2796 cells, a strain with no inherent DNA methylation. Genomic DNA was isolated from individual *E. coli* colonies expressing the protein of interest. The results from SMRT sequencing, Table 2 and Supplementary Figure S7, confirmed that the manipulated MTase genes were expressing active MTases *in vivo* in agreement with the results in Table 1 and Figure 2. The DNA recognition sequence for MS_fus_ was ACCN_5_RTGA as expected although some methylation of ACCN_5_RTGG was also detected. Analysis of M$^{1}\!\!/_{\!2}$S and M$^{1}\!\!/_{\!2}$S_fus_ show the novel enzymes methylating the palindromic sequence, ACCN_5_GGT. This finding also confirmed that the M$^{1}\!\!/_{\!2}$S and M$^{1}\!\!/_{\!2}$S_fus_ dimerise to recognise the palindromic target sequence.

**Table 2.** SMRT results determining the sites of methylation on the genomic DNA of *E. coli* ER2796 using cells transformed with plasmids expressing the wild type CC398 MTase, MS_fus_, M$^{1}\!\!/_{\!2}$S or M$^{1}\!\!/_{\!2}$S_fus_ proteins. MS_fus_ recognises the same target as the CC398 MTase as expected but also modifies some sites with the second part of the site having the sequence HRTGG rather than RTGA

Enzyme	Motifs	m6A modified position	% Motifs detected	Number of motifs detected	Number of motifs on genome
CC398 MTase	ACCNNNNNRTGA	1	99.69	971	974
	TCAYNNNNNGGT	3	99.69	971	974
MS_fus_	ACCNNNNNRTGA	1	76.88	1663	2163
	ACCNNNNHRTGG	1	69.46	523	753
	TCAYNNNNNGGT	3	76.05	1645	2163
M$^{1}\!\!/_{\!2}$S	ACCNNNNNGGT	1	78.64	2095	2664
M$^{1}\!\!/_{\!2}$S_fus_	ACCNNNNNGGT	1	99.66	2655	2664

## DISCUSSION

By making step-wise alterations to the structure of the CC398-1 Type I RM system, novel, soluble, sequence-specific enzymes have been engineered. As previously assumed from sequence comparisons (8), our results indicate that the Type I RM enzymes are close relatives of the IIB and IIG RM enzymes in terms of the organisation of their structural domains.

Although not restriction active, fusions of the nuclease domain of HsdR to the HsdM could be produced and purified in an assembly with the S subunit. Most of the purified material is a complex of RM_EB_2 with HsdS and a fragment of RM_EB_2 lacking the nuclease domain. This complex retains methylation activity but lacks nuclease activity suggesting that two nuclease domains are necessary. However, the successful expression and assembly of the fusion protein with HsdS suggests that our approach eventually could create a viable RM system, but that different lengths of HsdR would need to be fused to HsdM and tested before a fully active analogue of a Type IIB RM enzyme is obtained. To achieve full restriction and modification activity will require more optimisation but as the proteins are expressed, soluble and bind to the S subunit this is technically feasible although probably very challenging.

Manipulation of the *hsdM* and *hsdS* genes proved more successful and represents a step towards a Type IIG RM system. Three variants with HsdM fused to HsdS (MS_fus_), a half HsdS which dimerised to produce a ‘complete’ HsdS subunit (M$^{1}\!\!/_{\!2}$S) and a fusion of the half HsdS to HsdM which also dimerised (M$^{1}\!\!/_{\!2}$S_fus_) were all active MTases and, when complemented with HsdR, active restriction endonucleases. The modification was efficient *in vivo* as shown by the eop assay and the high motif coverage observed in the SMRT sequencing results. The target sites were as predicted from knowledge of the TRD specificity although the number of base pairs separating the methylation sites was increased by one base pair. The restriction *in vivo* was poor given the number of targets on phage λ (12 for ACCNNNNNRTGA and 14 for ACCNNNNNGGT) but in line with previous results with the Type I RM systems from *S. aureus* being expressed in *E. coli* (25–27). The observed eop for the restriction positive strains is poor when compared to the much higher levels of cutback observed with, for example, the EcoKI Type I RM system (eop = 10^−5^ typically) (42). This poor eop may be due to poor protein stability, inadequate expression of the HsdR subunits compared to the MTase subunits or a lack of target sites on the phage. Given the number of targets on phage λ for these enzymes it is more probable that poor stability, especially for M$^{1}\!\!/_{\!2}$S, and a mismatch in the intracellular concentrations of the HsdR and MTase proteins is responsible for the poor eop.

An unexpected result was observed in the new palindromic sequence, recognised by the dimerising M$^{1}\!\!/_{\!2}$S and M$^{1}\!\!/_{\!2}$S_fus_ fusion proteins. The sequence is a nucleotide shorter than that recognised by the wild-type enzyme, but the methylated bases are one bp further apart. This effect was not seen in the half HsdS enzymes created by others (28,29). The main role of the central conserved region has been assumed to be in separating the TRDs to a specific distance to recognise the bipartite target sequence but the new target spacing suggests that there is not a simple relationship between the length of the central conserved region and the DNA target.

In theory, one final step could be taken to fuse the nuclease domain of HsdR to the M$^{1}\!\!/_{\!2}$S_fus_ fusion protein to give an arrangement of domains identical to a Type IIG RM enzyme shown in Figure 1A. Our results would suggest that such a protein would be expressed and active as a MTase but that the nature of the fusion of the nuclease domain to MTase would need much optimisation in the laboratory to produce a fully functional RM enzyme. Over long evolutionary time periods, Nature has presumably made many attempts to make the Type IIG RM enzymes by fusing the nuclease domain of HsdR to the M$^{1}\!\!/_{\!2}$S_fus_ fusion protein. Most of these fusions have failed to function and been eliminated but at least one attempt must have succeeded and spread through bacterial populations such that today Type IIG RM systems are almost as prevalent as the Type I RM systems, Table 3.

**Table 3.** The number of restriction enzymes, both confirmed and putative, in REBASE as at April 2018. The numbers were derived by simply counting the number of R subunits for the Type I RM systems, the number of REases for the Type II systems and Type IV systems and the number of res subunits for the Type III RM systems. The proportions are very similar to those obtained previously by Oliviera *et al.*, (5)

RM system	Number of examples	Percentage of total	Percentage of total ignoring Type IV
Type I REase	18580	26.6	31.8
Type IIP REase	13648	19.6	23.4
Type II REase (not IIP or IIG)	7144	10.2	12.2
Type IIG REase/MTase	11737	16.8	20.1
Type III REase	7305	10.5	12.5
Type IV REase	11356	16.3	-
Total	69770	100	100

### A structural model for the evolution of RM systems

This work aimed to alter a Type I RM system to structural forms resembling ‘simpler’ Types of RM systems to provide evidence for the theory that the RM Types are evolutionarily linked. The Type I, IIG and III systems are by far the most common RM systems in bacteria and archaea and are well conserved in terms of genetic structure and protein structure reusing the same domains in different combinations (5,8,16,70), Figure 1A. Table 3 shows the relative proportions of the different RM Types with Type I, IIG and III making up ∼55% of known and putative RM systems in REBASE (6). The methylation-dependent Type IV restriction systems make up a further 16% leaving the most well-known Type IIP plus Type IIS and other unassigned Type II RM systems to make up the remaining ∼30% of RM systems.

**Figure 3.**
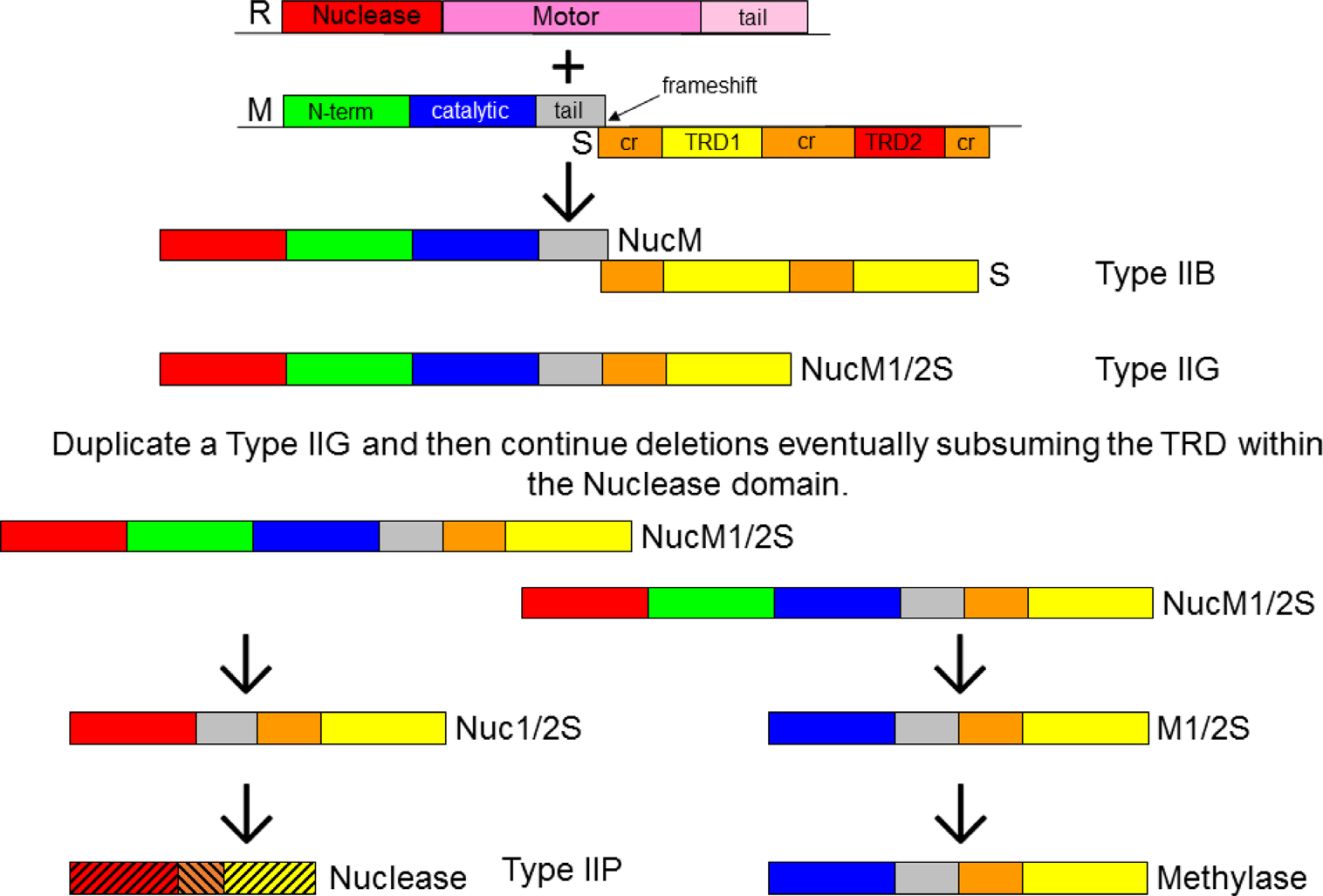
Schematic diagram delineating the steps that could be taken to alter the structure of a Type I RM system to create Type II systems. The colour scheme for domains is the same as in Figure 1. In the step from Nuc$^{1}\!\!/_{\!2}$S to Nuclease, the domains are merging together so are shown with hatching.

Our results linking different Types of RM system suggest an evolutionary model for the diversity of RM enzymes. The model, shown in Figure 3, is based upon suggesting answers to the questions posed in the introduction and makes specific proposals for experimental tests as described below.

When did RM first appear in evolutionary history and why did it appear?

It would seem very probable that RM systems appeared as soon as horizontal gene transfer (HGT) between early cells arose and they came in contact with the first phage or other mobile genetic elements (71–74). RM would help any host cell to control the rate of uptake of foreign DNA and this would probably be crucial to generate stability in the primitive genome. If HGT was not controlled, then it is difficult to see how a stable genome could be established and maintained in an environment with a lot of mobile genetic elements (MGE). Conversely too much control of HGT would slow down the subsequent evolution of the RM host as no foreign DNA on MGE could enter the host which would then have to rely solely upon vertical evolution.

What did the first RM system look like?

The first RM system would have to be constructed from whatever components were present in the early cell. It seems to us to be highly improbable that classical Type IIP RM systems with separate REase and MTase enzymes using entirely different chemistries to perform their separate reactions while recognising the exact same DNA target would evolve easily. The classical Type IIP RM systems are not ‘simple’ systems despite their small size in molecular weight terms.

We suggest that the first RM enzyme would be akin to a Type I RM enzyme as the subunits for these enzymes have close homologues in ancient essential enzymes and proteins vital for genome repair, nucleotide modification and regulation of transcription. When an early cell evolved a primitive RM defense system, it would have to build upon the components already present. Maintenance of an early genome would require DNA repair enzymes and these frequently contain ATP-dependent DEAD-box SFII superfamily proteins of which HsdR is a member (75). Nucleotide methylation utilises an ancient SAM-dependent fold and this fold is found in the MTases used in all RM systems (76,77). Gene regulation using transcription factors to recognise long palindromic sequences often use homodimeric proteins perhaps akin to the dimers of half-HsdS. For instance, the BmrR repressor recognises split DNA targets similar to the targets recognised by the Type I RM systems containing half-HsdS and its structure is strikingly similar to HsdS (78). When under attack from phage, any early cell that could assemble an RM system from these components would have an advantage.

Why did it subsequently evolve to form the large range of RM structural variants observed today?

This early ‘Type I’ RM enzyme built from pre-existing components would subsequently evolve to form other ‘simpler’ RM systems by the sorts of domain fusions/deletions investigated in this work and elsewhere (28,29) under the evolutionary pressure of antirestriction (79–81). Most antirestriction / antimodification (anti-RM) systems are known to be directed at the Type I RM systems (79). These anti-RM systems, such as DNA mimics (81), would provide the evolutionary pressure to force the early ‘Type I’ RM system to evolve to an anti-RM-resistant form. These anti-RM-resistant forms would, by definition, be the Type II and Type III RM systems. It appears the Type II and Type III RM systems are not targeted by the existing DNA mimics but this clearly needs further investigation as so few have been tested for inhibition by a DNA mimic (82). The first step in this ‘simplification’ of the structure of the RM enzyme would appear to be via the evolution of the Type IIB and IIG RM enzymes by the gene fusions and deletions investigated here. Once a cell had evolved a RM system resistant to the MGE-borne DNA mimics, the MGE would then be under further pressure to evolve new anti-RM mechanisms and it appears that target site avoidance and nucleotide modifications are particularly prevalent choices (79,83).

What was the evolutionary pathway for the appearance of these structural variants?

If the proposed scenario is at least approximately correct then what happened after the appearance of the Type IIB/G enzymes to generate the Type IIP and Type III RM systems?

Type III RM systems, composed of ‘res’ and ‘mod’ subunits (9), would appear to use a variant of the HsdR subunit plus a fused HsdM-HsdS where there has been some circular permutation to move the *hsdS* to within the *hsdM* gene (17,18,43,53). The relative locations of the TRD with respect to the motifs for SAM binding subdivide all SAM-dependent MTases into six groups, α to ζ, with the γ group predominating (17,43). This circular permutation, with different relative locations of the amino acid motifs of the SAM-binding domain with respect to the TRD, has also occurred in some Type II RM systems with separate MTases leading to the formation of α and β subgroups from the γ subgroup, which is the main subgroup in the Type I and IIB/G RM systems (17,43,84).

To generate separate REase and MTase as found in ‘classical’ Type IIP RM systems would require a gene duplication of the nascent Type IIG gene followed by evolution of the REase by deletion of the M domain and evolution of the MTase by loss of the nuclease domain and in both cases retaining the TRD(s), Figure 3. At this point, due to the selfish nature of RM systems with separate R and M (11), the MTase would be under pressure to maintain sequence specificity while the REase could start to mutate to obscure the amino acid conservation present immediately after the duplication event. The REase could even acquire new functions (3).

Of course, one may ask why the Type IIP REase has two ‘TRD’ regions to recognise a palindromic target while the cognate MTase has only a single different TRD? This would seem to be a problem with our model. However, very few RM systems have structures known for both REase and MTase (PvuII seems to be the only pair apart from those with combined R and M) (85,86) and there is a regrettably little information on the quaternary structure of the active enzymes. Of note here is that many simple Type II MTases appear to exist as dimers despite the fact that they are usually assumed to operate as monomers (44). A dimeric MTase may have two TRDs but only use one at a time as this would allow them to simultaneously scan both DNA strands or to move around on DNA via DNA looping (87). Furthermore, many REases appear to require rather complicated quaternary structures to assemble on DNA to be active (88–92). Recent structures of some REases show distinct similarity to an HsdS subunit (54) and this would support our suggestion that a proto-IIG enzyme lost its MTase domain resulting in a fusion of a nuclease domain to a half-HsdS domain which would then dimerise to produce a IIP REase with a homodimeric structure and a palindromic target (the cognate MTase would lose the nuclease domain instead). One of these REases, R.SwaI, recognises 5′-ATTTAAAT-3′ and the two long alpha helices forming the dimer interface have their helical axes almost perpendicular to the DNA helical axis (54), Supplementary Figure S8. This arrangement places the two nuclease domains close to the centre of the target to allow cutting at the centre to produce blunt ends. A similar arrangement is found with R.HincII which recognises GTYRAC and makes a blunt cut (91,92), Supplementary Figure S8. In comparison, models of HsdS bound to DNA (22,24) have the equivalent alpha helices aligned nearly parallel with the DNA helical axis and this places the TRDs far apart to allow recognition of the classic bipartite Type I target sequence, Supplementary Figure S8. Perhaps by changing the angle between helical axes of the alpha helices and the DNA helix, Nature has been able to bring the DNA recognition domains of a proto-half-HsdS closer together to recognise shorter targets and to incorporate the necessary features of the nuclease domain to facilitate DNA cleavage? Even Type IIP REases without such an obvious structural relationship to an HsdS use extensive alpha helical regions to form the interface between the two subunits in a similar manner to the interface formed between two half-HsdS monomers (12).

Our model may also suggest why C5C methylation uses a very highly conserved MTase domain and why it is found predominantly in Type II MTases and hardly ever in other RM Types. Type I, IIB and IIG MTases, almost without exception, are N6A or N4C MTases (93). Some are capable of methylating both as they can be tricked into methylating the wrong nucleotide (85) but they do share a common MTase domain architecture which is very ancient (76). The MTase N6A/N4C catalytic domain, although having relatively few conserved amino acid motifs, has a well conserved fold and catalytic site (7,43,70). The MTase domain in a C5C MTase has exactly the same fold as the N6A and N4C MTases but the amino acid sequence is much better conserved and 10 motifs, including motifs equivalent to those in N6A and N4C MTases, are easily recognised (7,43,70). This suggests that C5C methylation is a more recent innovation in evolution and that it is a difficult chemistry to evolve as it would require multiple, simultaneous amino acid changes in a precursor N4C MTase domain (12). Thus, C5C methylation chemistry probably appeared once and, by chance, became the main methylation found in eukaryotes.

### Further experiments suggested by the structural model

Previously evolutionary models have been proposed for the Type II RM systems (17,95,96) and similarities between several subgroups of the Type II RM systems and the Type I and III RM systems have been noted (7,8,45–49). However, when our results are coupled with these evolutionary models it is possible to suggest a ‘global’ model the majority of the defined ‘Types’ of RM system (excepting the Type IV restriction systems). Of course, our model shown in Figure 3 is not all-encompassing as there are other Type II RM variants such as IIS and those using other nuclease chemistries which exist, but these are a small proportion of the total number of RM systems (5,6). However, our model covers the great majority of known RM systems (5).

The model, even if subsequently proven to be incorrect, highlights several areas of RM research that have been neglected and which should be investigated. These areas are listed below. All of these suggestions are readily addressed due to easy availability of purified commercial RM enzymes.

Perhaps surprisingly, there are very few structures of REases and MTases from the same RM system (85,86). More RM ‘pairs’ from Type IIP RM systems should be solved to see if they use similar structural features to recognise their DNA target sequence. In other words, do the separate REase and MTase show any sign of a conserved ancestral TRD?

Anti-RM DNA mimics have never been tested against the majority of RM systems other than the Type I RM systems (82). Given the commercial availability of many REases and MTases, anti-RM versus the Type II RM enzymes could also easily be investigated to see if the DNA mimics targeting the Type I RM enzymes are more versatile than currently assumed. The phage T7 ocr DNA mimic (97,98) and the ArdA DNA mimic from conjugative plasmids and transposons (56,57,99) could be easily employed for this purpose but further anti-RM proteins such as T3 SAMase (100), Ral (101) and Lar (102) could be investigated.

The quaternary structure of the RM enzymes when bound to DNA needs to be examined as, in the few examples studied, drastic changes in their structure upon binding have been observed (91). Electron microscopy would seem to be a particularly useful technique in this area (91), especially if applied to the large protein-DNA complexes required for the REase activity of the Type IIB RM enzymes (89). Atomic force microscopy, although of lower resolution than electron microscopy, may also be useful for elucidating DNA topology in these complexes (90,103–105).

The evolution of methylation of cytosine at the C5 position is clearly difficult as it appears to have only occurred relatively recently and only in the Type II RM systems. Assuming that the predecessor of C5C methylation was N4C methylation, it may be possible to mutate the catalytic site in a N4C MTase to recognise and methylate C5C (or *vice versa* which may be an easier experiment to pursue in practice) with a few amino acid changes. In this vein, and as suggested by the structure of the SwaI REase (54), it may also be possible to mutate a TRD in a MTase or HsdS subunit to incorporate the PD-(D/E)XK nuclease motif and generate a new REase.

## Supplementary Material

Supplementary Data
