# Supplementary material for: A model for the evolution of prokaryotic DNA restriction-modification systems based upon the structural malleability of Type I restriction-modification enzymes
Source: Nucleic Acids Res. 2018 Aug 28;46(17):9067–80. doi: 10.1093/nar/gky760 (PMC6158711; doi:10.1093/nar/gky760)
Supplement: Supplementary Data [file gky760_supplemental_files.pdf]

A model for the evolution of prokaryotic DNA restriction-modification systems based upon the structural malleability of Type I restriction-modification enzymes.

Edward K.M. Bower, Laurie P. Cooper, Gareth A. Roberts, John H. White, Yvette Luyten, Richard D. Morgan, David T.F. Dryden

Supplementary Information

Table S1. Amino acid sequences for the HsdR, HsdS and HsdS used in this work.

HsdR subunit (SauN315ORF189P 929 aa)
MAYQSEYALE NEMMNQLEQL GYERVTIRDN KQLLDNFRTI LNERHADKLE GNPLTDKEFQ RLLTMIDGKS IFESARILRD
KLPLRRDDES EIYLSFLDKK SWCKNKFQVT NQVSVEDTYK ARYDVTILIN GLPLVQVELK RRGIDINEAF NQVKRYRKQN
YTGLFRYIQM FIISNGVETR YFSNNDSELL KSHMFYWSDK QNNRINTLQS FAESFMRPCQ LAKMISRYMI INETDRILMA
MRPYQVYAVE ALIQQATETG NNGYVWHTTG SGKTLTSFKA SQILSQQDDI KKVIFLVDRK DLDSQTEEEF NKFAKGAVDK
TFNTSQLVRQ LNDKSLPLIV TTIQKMAKAI QGNAHLLEQY KTNKVVFIID ECHRSQFGDM HRLVKQHFKN AQYFGFTGTP
RFPENSSQDG RTTADIFGRC LHTYLIRDAI HDGNVLGFSV DYINTFKNKA LKAEDNSMVE AIDTEEVWLA DKRVELVTRH
IINNHDKYTR NRQYSSIFTV QSIHALIKYY ETFKRLNKKL EQPLTIAGIF TFKPNEDDRD GEVPYHSREK LEIMISDYNK
KFETNFSTDT TNEYFNHISK NVKKGVKDSK IDILIVVNMF LTGFDSKVLN TLYVDKNLMY HDLIQAYSRT NRVEKESKPF
GKIVNYRDLK KETDDALRVF SQTNDTDTIL MRSYEEYKKE FMDAYRELKM IVPTPHMVDD IQDEEELKRF VEAYRLLAKI
ILRLKAFDEF EFTIDEIGMD EQENEDYKSK YLAVYDQVKR ATAEKNKVSI LNDIDFEIEM MRNDTINVNY IMNILRQIDL
EDKAEQRRNQ EQIRRILDHA DDPTLRLKRD LIREFIDNVV PSLNKDDDID QEYVNFESIK KEAEFKGFAG ERSIDEQALK
TISNDYQYSG VVNPHHLKKM IGDLPLKEKR KARKAIESFV AETTEKYGV
HsdR subunit nucleotide sequence (SauN315ORF189P 2790 nt)
ATGGCATACCAAAGTGAATACGCATTAGAAAATGAAATGATGAATCAACTTGAACAATTGGGTTACGAAAGAGTAACGATACGTGATAATAA
GCAATTGCTTGATAATTTTAGAACGATTTTAAATGAGCGTCATGCGGACAAATTAGAAGGCAATCCCTTAACAGATAAAGAATTTCAACGTC
TGTTAACGATGATTGATGGGAAAAGTATTTTCGAGAGTGCCCGTATTTTACGTGATAAATTACCACTTAGACGTGATGATGAGTCTGAGATT
TATTTGTCGTTTTTAGATAAGAAAAGTTGGTGTAAAAATAAGTTTCAAGTGACGAATCAAGTATCTGTCGAGGATACATATAAAGCACGTTA
TGATGTAACGATATTAATCAACGGACTACCCCTTGTCCAAGTTGAATTGAAACGTCGAGGTATTGATATTAATGAGGCGTTTAACCAAGTAA
AACGTTACCGCAAACAAAATTACACAGGCTTATTCCGCTACATACAAATGTTTATCATTAGTAATGGTGTTGAAACGCGATACTTTTCTAAT
AATGATAGCGAACTATTGAAGAGTCACATGTTTTATTGGAGTGATAAACAGAATAACCGAATCAATACATTACAATCATTTGCTGAGTCGTT
TATGAGACCCTGTCAATTAGCTAAGATGATATCGCGCTATATGATTATTAATGAAACAGATAGAATACTGATGGCAATGCGTCCGTATCAAG
TGTATGCGGTAGAAGCACTTATTCAACAAGCGACTGAGACAGGGAATAATGGATATGTATGGCATACAACTGGAAGTGGTAAGACATTGACT
TCTTTTAAAGCGAGTCAGATTTTATCACAGCAAGATGACATTAAGAAAGTTATCTTTTTGGTTGACCGTAAAGACTTGGATAGTCAAACAGA
AGAGGAATTTAATAAATTTGCTAAGGGTGCTGTAGACAAAACTTTTAATACCTCGCAACTGGTACGCCAACTAAATGATAAAAGTTTGCCAC
TTATTGTAACGACGATTCAAAAAATGGCTAAAGCGATTCAAGGGAATGCCCATTTATTAGAACAGTATAAAACGAATAAAGTTGTATTTATT
ATTGATGAGTGTCATCGCAGTCAATTTGGTGACATGCATCGTCTAGTTAAACAACATTTCAAAAATGCCCAATACTTTGGATTCACTGGTAC
GCCACGTTTTCCAGAAAATAGTAGTCAAGATGGTAGAACAACTGCAGATATTTTCGGTAGATGCTTACATACGTATTTAATTAGAGATGCCA
TTCATGATGGTAATGTACTTGGTTTCTCAGTTGACTATATTAATACTTTTAAAAATAAAGCTTTAAAAGCAGAAGATAACAGCATGGTTGAA
GCAATTGATACGGAAGAAGTATGGTTAGCGGATAAACGTGTGGAATTAGTAACACGACATATCATCAATAATCATGATAAATATACACGTAA
TCGTCAATATTCAAGTATATTTACAGTCCAAAGTATTCACGCGCTTATTAAATATTATGAGACATTTAAGCGACTTAACAAAAAGTTGGAAC
AACCGTTAACGATAGCTGGTATATTTACGTTTAAACCTAATGAAGATGATCGTGATGGTGAAGTGCCATATCATTCACGTGAAAAATTAGAG
ATAATGATTAGTGATTATAATAAAAAGTTCGAGACGAATTTTTCAACAGACACAACTAATGAGTATTTTAATCATATTTCAAAAAACGTTAA
AAAGGGCGTTAAAGATAGTAAAATTGATATCTTAATCGTTGTTAATATGTTCTTAACTGGTTTTGATAGTAAAGTACTGAACACTTTATATG
TTGATAAGAATTTAATGTATCATGATTTAATTCAAGCGTATTCACGTACAAATAGGGTTGAAAAAGAATCAAAGCCATTTGGTAAAATTGTA
AACTATCGTGACTTGAAAAAAGAGACAGACGATGCACTGAGAGTATTCTCACAAACAAATGATACGGATACAATTTTAATGCGCAGTTATGA
AGAGTATAAAAAAGAATTTATGGACGCTTATCGTGAGCTTAAAATGATTGTGCCGACACCACACATGGTTGATGACATTCAAGATGAAGAAG
AGCTAAAGCGCTTTGTTGAAGCTTATCGTTTATTAGCTAAAATAATATTACGTTTAAAAGCATTTGACGAGTTTGAGTTTACAATTGATGAA
ATTGGAATGGATGAACAAGAGAATGAAGACTATAAAAGTAAATATTTAGCTGTGTACGATCAAGTAAAAAGAGCGACGGCTGAGAAAAATAA
AGTATCCATTTTAAATGATATTGATTTCGAAATAGAAATGATGCGTAATGATACGATTAATGTGAATTATATTATGAATATATTGAGACAAA
TTGATCTTGAAGACAAAGCGGAACAACGTCGTAACCAAGAACAAATTAGACGCATTTTAGATCATGCAGATGATCCGACATTGAGGTTAAAA
CGAGATCTAATTAGAGAATTCATCGACAATGTTGTACCTTCTTTAAATAAGGATGATGATATCGATCAAGAATATGTTAATTTCGAAAGTAT
TAAAAAAGAAGCGGAGTTCAAAGGATTTGCTGGAGAGAGATCTATCGATGAACAAGCCCTAAAAACAATTTCAAATGACTACCAGTATAGTG
GTGTTGTAAACCCACATCACCTTAAAAAAATGATTGGTGATTTGCCATTGAAAGAAAAGCGTAAAGCAAGAAAAGCCATTGAATCTTTCGTG
GCAGAAACAACTGAAAAATACGGTGTG

HsdM (M.SauSTORF499P 518 aa)
MSITEKQRQQ QAELHKKLWS IANDLRGNMD ASEFRNYILG LIFYRFLSEK AEQEYADALS GEDITYQEAW ADEEYREDLK
AELIDQVGYF IEPQDLFSAM IREIETQDFD IEHLATAIRK VETSTLGEES ENDFIGLFSD MDLSSTRLGN NVKERTALIS
KVMVNLDDLP FVHSDMEIDM LGDAYEFLIG RFAATAGKKA GEFYTPQQVS KILAKIVTDG KDKLRHVYDP TCGSGSLLLR
VGKETQVYRY FGQERNNTTY NLARMNMLLH DVRYENFDIR NDDTLENPAF LGHTFDAVIA NPPYSAKWTA DSKFENDERF
SGYGKLAPKS KADFAFIQHM VHYLDDEGTM AVVLPHGVLF RGAAEGVIRR YLIEEKNYLE AVIGLPANIF YGTSIPTCIL
VFKKCRQQDD NVLFIDASND FEKGKNQNHL SDAQVERIID TYKRKETIDK YSYSATLQEI ADNDYNLNIP RYVDTFEEEA
PIDLDQVQQD LKNIDKEIAE VEQEINAYLK ELGVLKDE
HsdM nucleotide sequence (M.SauSTORF499P 1557 nt)
ATGTCTATTACTGAAAAACAACGTCAGCAACAAGCTGAATTACATAAAAAATTATGGTCGATTGCGAATGATTTAAGAGGGAATATGGATGC
GAGTGAATTCCGTAATTACATTTTAGGCTTGATTTTCTATCGCTTCTTATCTGAAAAAGCGGAACAAGAATACGCAGATGCCTTGTCAGGTG
AAGACATCACGTATCAAGAAGCATGGGCAGATGAAGAATACCGTGAAGACTTAAAAGCAGAATTAATTGATCAAGTTGGTTACTTTATTGAA
CCGCAAGATTTATTCAGCGCGATGATTCGTGAAATTGAAACGCAAGATTTCGATATCGAACATCTAGCGACGGCAATTCGTAAAGTTGAAAC
ATCAACATTAGGTGAAGAAAGTGAAAATGACTTTATCGGACTATTCAGCGATATGGATTTGAGTTCAACGCGACTAGGTAACAATGTCAAAG
AACGTACTGCGTTAATCTCTAAAGTCATGGTTAATCTTGACGACTTACCATTTGTTCACAGTGATATGGAAATTGATATGTTAGGTGATGCA
TACGAATTCCTTATCGGGCGCTTTGCGGCGACAGCGGGTAAAAAAGCTGGCGAGTTCTATACACCACAACAAGTATCTAAGATACTGGCGAA
GATTGTCACAGACGGTAAAGATAAATTACGTCATGTATATGACCCAACATGTGGTTCAGGTTCATTACTGTTACGTGTAGGTAAAGAAACGC
AAGTGTATCGTTATTTCGGTCAAGAACGTAACAATACCACATACAACTTAGCGCGCATGAACATGTTATTACATGATGTACGTTATGAAAAT
TTCGATATACGTAATGATGACACGTTGGAAAATCCAGCCTTTTTAGGCCATACATTTGATGCGGTTATTGCGAACCCACCATACAGTGCGAA

ATGGACAGCAGATTCAAAATTTGAAAATGACGAACGATTCAGCGGATACGGCAAGCTTGCGCCAAAGTCTAAAGCAGACTTTGCCTTTATTC
AACACATGGTACATTACCTAGATGATGAAGGTACCATGGCAGTTGTACTACCGCATGGTGTCTTATTCCGTGGGGCTGCAGAAGGCGTCATT
CGTCGTTACTTAATTGAAGAAAAGAACTACCTAGAAGCCGTGATTGGCTTACCAGCGAATATTTTCTATGGGACAAGTATTCCAACATGTAT
TTTAGTATTTAAAAAATGTCGCCAACAAGACGACAACGTATTATTTATCGATGCATCCAATGATTTTGAAAAAGGAAAAAATCAAAACCATT
TAAGCGACGCCCAAGTCGAACGCATTATAGACACATATAAGCGTAAAGAAACAATCGATAAATATAGCTACAGCGCGACATTACAAGAGATT
GCCGATAACGATTACAACTTAAACATTCCGAGATATGTCGATACATTCGAAGAAGAAGCGCCAATTGATTTAGATCAAGTCCAACAAGATTT
GAAAAATATCGACAAAGAAATCGCAGAAGTTGAACAAGAAATCAATGCATACCTGAAAGAACTTGGGGTGTTGAAAGATGAG

HsdS (S.SauSTORF499P with His tag recognising ACCNNNNNRTGA)

MSNTQKKNVP ELRFPGFEGE WEEKKLGEFA GKVTQKNVDK KYIETLTNSA ELGIISQKDY FDKEISNIDN IKKYYVVEEN
DFVYNPRMSN YAPFGPVNRN KLGKKGVMSP LYTVFKIQNI DLNFIEFYFK SSKWYRFMAL NGDSGARADR FSIKDRTFME
MPLHIPCMDE QIKIGQFFSK LDRQIELEEQ KLELLQQQKK GYMQKIFSQE LRFKDENGKD YPEWEETTIK EIAQINTGKK
DTKDAITNGS YDFYVRSPIV YKINTFSYEG EAILTVGDGV GVGKVFHYVN GKFDYHQRVY KISDFKNYYG LLLFYYFSQN
FLKETKKYSA KTSVDSVRKD MIANMKVPRP IYIEQKKIGQ FIKRVDNKTK IQKQVIELLK QRKKSLLQKM FIPGGSHHHH HH

HsdS nucleotide sequence (S.SauSTORF499P with His tag)

ATGAGTAATACACAAAAGAAAAATGTGCCAGAGTTGAGATTCCCCGGGTTTGAAGGCGAATGGGAAGAGAAGAAGCTAGGTGAGTTTGCTGG
TAAAGTTACCCAAAAAAATGTTGATAAAAAATATATTGAGACATTAACTAATTCATCTGAGTTAGGTATCATATCTCAAAAGGATTATTTTG
ACAAAGAAATTTCGAATATAGATAATATTAAAAAGTACTATGTAGTTGAAGAGAATGATTTTGTTTATAACCCTAGAATGTCTAATTATGCT
CCATTTGGACCAGTAAATAGAAATAAGTTAGGGAAAAAAGGGGTCATGTCACCTCTTTATACTGTGTTTAAAATTCAAAACATTGATTTAAA
CTTTATTGAGTTTTATTTTAAATCTTCAAAATGGTATAGATTTATGGCATTAAACGGTGATTCAGGTGCTCGAGCAGATAGGTTTTCTATTA
AAGATAGGACATTTATGGAAATGCCACTTCATATCCCATGTATGGATGAACAAATAAAAATCGGTCAGTTCTTCAGCAAACTTGACCGACAA
ATTGAATTAGAAGAACAAAAACTTGAATTACTTCAACAACAGAAAAAAGGCTATATGCAGAAAATCTTCTCGCAAGAATTGCGATTTAAAGA
TGAGAATGGTAAAGATTATCCGGAGTGGGAAGAAACTACTATAAAAGAAATTGCTCAAATTAACACTGGAAAGAAAGATACAAAAGATGCCA
TTACTAATGGGAGTTATGATTTTTACGTTAGATCTCCGATAGTTTATAAAATTAATACTTTTAGTTATGAAGGAGAGGCTATTTTAACTGTA
GGAGATGGAGTTGGCGTAGGTAAAGTTTTCCACTATGTAAATGGGAAATTTGATTATCATCAAAGAGTATACAAAATATCTGACTTTAAGAA
TTATTATGGATTATTGTTATTTTATTATTTTTCACAAAACTTTTTAAAAGAAACAAAGAAATATAGTGCGAAGACATCAGTTGATTCAGTTA
GAAAAGACATGATTGCTAATATGAAAGTACCGCGTCCTATTTATATAGAACAAAAAAAAATCGGTCAATTCATTAAAAGAGTAGACAACAAA
ACAAAAATTCAGAAACAAGTGATTGAATTACTTAAACAACGCAAAAAGTCATTACTTCAAAAGATGTTTATTCCCGGGGGATCCCATCATCA
TCATCATCATT

Table S2. Oligonucleotide sequences for fusion of nuclease domain of *hsdR* to 5' end of *hsdM* and the amino acid sequences of the part of HsdR fused to HsdM. Nucleotide sequences in bold are part of the gene coding sequence.

Fusion Name	PCR oligonucleotide name	Nucleotide Sequence
RM_EB_1 (not used)	Mu50nucendalpha-CC398-1TS-	5'**GCACTTATTCAACAAGCGACT**ATGTCTATTACTGAAAAACAACG 3'
	Mu50nucendalpha-CC398-1BS-	5'CGTTGTTTTTCAGTAATAGACAT**AGTCGCTTGTTGAATAAGTGC** 3'
RM_EB_2	Mu50nucendcoil-CC398-1TS	5'**CGACTGAGACAGGGAATAAT**ATGTCTATTACTGAAAAACAACG 3'
	Mu50nucendalpha-CC398-1BS	5'CGTTGTTTTTCAGTAATAGACAT**ATTATTCCCTGTCTCAGTCG** 3'
RM_EB_3	Mu50nuclease- CC398-1TS	5'**CATTTGCTGAGTCGTTTATGAGA**ATGTCTATTACTGAAAAACAACG 3'
	Mu50nuclease- CC398-1BS	5'CGTTGTTTTTCAGTAATAGACAT**TCTCATAAACGACTCAGCAAATG** 3'
RM_EB_4	RM_EB_7 TS	5'**CAGAATAACCGAATCAATACA**ATGTCTATTACTGAAAAACAACG 3'
	RM_EB_7 BS	5'CGTTGTTTTTCAGTAATAGACAT**TGTATTGATTCGGTTATTCTG** 3'
RM_EB_5	RM_EB_8 TS	5'**CAATCATTTGCTGAGTCGTTT**ATGTCTATTACTGAAAAACAACG 3'
	RM_EB_8 BS	5'CGTTGTTTTTCAGTAATAGACAT**AAACGACTCAGCAAATGATTG** 3'
RM_EB_6	RM_EB_9 TS	5'**CCCTGTCAATTAGCTAAGATGATA**ATGTCTATTACTGAAAAACAACG 3'
	RM_EB_9 BS	5'CGTTGTTTTTCAGTAATAGACAT**TATCATCTTAGCTAATTGACAGGG** 3'
RM_EB_7	RM_EB_10 TS	5'**CTGATGGCAATGCGTCCGTAT**ATGTCTATTACTGAAAAACAACG 3'
	RM_EB_10 BS	5'CGTTGTTTTTCAGTAATAGACAT**ATACGGACGCATTGCCATCAG** 3'
RM_EB_8	RM_EB_11 TS	5'**CCGCTACATACAAATGTTT**ATGTCTATTACTGAAAAACAACG 3'
	RM_EB_11 BS	5'CGTTGTTTTTCAGTAATAGACAT**AAACATTTGTATGTAGCGG** 3'
RM_EB_9	RM_EB_12 TS	5'**GGTGTTGAAACGCGATACTTTTCT**ATGTCTATTACTGAAAAACAACG 3'
	RM_EB_12 BS	5'CGTTGTTTTTCAGTAATAGACAT**AGAAAAGTATCGCGTTTCAACACC** 3'
RM_EB_10	RM_EB_13 TS	5'**GGCATACAACTGGAAGTGGT**ATGTCTATTACTGAAAAACAACG 3'
	RM_EB_13 BS	5'CGTTGTTTTTCAGTAATAGACAT**ACCACTTCCAGTTGTATGCC** 3'
RM_EB_11	RM_EB_14 TS	5'**GCGAGTCAGATTTTATCA**ATGTCTATTACTGAAAAACAACG 3'
	RM_EB_14 BS	5'CGTTGTTTTTCAGTAATAGACAT**TGATAAAATCTGACTCGC** 3'

Fusion Name	Amino acid sequence from HsdR
RM_EB_2	MAYQSEYALE NEMMNQLEQL GYERVTIRDN KQLLDNFRTI LNERHADKLE GNPLTDKEFQ RLLTMIDGKS IFESARILRD KLPLRRDDES EIYLSFLDKK SWCKNKFQVT NQVSVEDTYK ARYDVTILIN GLPLVQVELK RRGIDINEAF NQVKKRYRKQN YTGLFRYIQM FIISNGVETR YFSNNDSELL KSHMFYWSDK QNNRINTLQS FAESFMRPCQ LAKMISRYMI INETDRILMA MRPYQVYAVE ALIQQATETG NN
RM_EB_3	MAYQSEYALE NEMMNQLEQL GYERVTIRDN KQLLDNFRTI LNERHADKLE GNPLTDKEFQ RLLTMIDGKS IFESARILRD KLPLRRDDES EIYLSFLDKK SWCKNKFQVT NQVSVEDTYK ARYDVTILIN GLPLVQVELK RRGIDINEAF NQVKKRYRKQN YTGLFRYIQM FIISNGVETR YFSNNDSELL KSHMFYWSDK QNNRINTLQS FAESFMR
RM_EB_4	MAYQSEYALE NEMMNQLEQL GYERVTIRDN KQLLDNFRTI LNERHADKLE GNPLTDKEFQ RLLTMIDGKS IFESARILRD KLPLRRDDES EIYLSFLDKK SWCKNKFQVT NQVSVEDTYK ARYDVTILIN GLPLVQVELK RRGIDINEAF NQVKKRYRKQN YTGLFRYIQM FIISNGVETR YFSNNDSELL KSHMFYWSDK QNNRINT
RM_EB_5	MAYQSEYALE NEMMNQLEQL GYERVTIRDN KQLLDNFRTI LNERHADKLE GNPLTDKEFQ RLLTMIDGKS IFESARILRD KLPLRRDDES EIYLSFLDKK SWCKNKFQVT NQVSVEDTYK ARYDVTILIN GLPLVQVELK RRGIDINEAF NQVKKRYRKQN YTGLFRYIQM FIISNGVETR YFSNNDSELL KSHMFYWSDK QNNRINTLQS FAESF
RM_EB_6	MAYQSEYALE NEMMNQLEQL GYERVTIRDN KQLLDNFRTI LNERHADKLE GNPLTDKEFQ RLLTMIDGKS IFESARILRD KLPLRRDDES EIYLSFLDKK SWCKNKFQVT NQVSVEDTYK ARYDVTILIN GLPLVQVELK RRGIDINEAF NQVKKRYRKQN YTGLFRYIQM FIISNGVETR YFSNNDSELL KSHMFYWSDK QNNRINTLQS FAESFMRPCQ LAKMI
RM_EB_7	MAYQSEYALE NEMMNQLEQL GYERVTIRDN KQLLDNFRTI LNERHADKLE GNPLTDKEFQ RLLTMIDGKS IFESARILRD KLPLRRDDES EIYLSFLDKK SWCKNKFQVT NQVSVEDTYK ARYDVTILIN GLPLVQVELK RRGIDINEAF NQVKKRYRKQN YTGLFRYIQM FIISNGVETR YFSNNDSELL KSHMFYWSDK QNNRINTLQS FAESFMRPCQ LAKMISRYMI INETDRILMA MRP
RM_EB_8	MAYQSEYALE NEMMNQLEQL GYERVTIRDN KQLLDNFRTI LNERHADKLE GNPLTDKEFQ RLLTMIDGKS IFESARILRD KLPLRRDDES EIYLSFLDKK SWCKNKFQVT NQVSVEDTYK ARYDVTILIN GLPLVQVELK RRGIDINEAF NQVKKRYRKQN YTGLFRYIQM F
RM_EB_9	MAYQSEYALE NEMMNQLEQL GYERVTIRDN KQLLDNFRTI LNERHADKLE GNPLTDKEFQ RLLTMIDGKS IFESARILRD KLPLRRDDES EIYLSFLDKK SWCKNKFQVT NQVSVEDTYK ARYDVTILIN GLPLVQVELK RRGIDINEAF NQVKKRYRKQN YTGLFRYIQM FIISNGVETR YFS
RM_EB_10	MAYQSEYALE NEMMNQLEQL GYERVTIRDN KQLLDNFRTI LNERHADKLE GNPLTDKEFQ RLLTMIDGKS IFESARILRD KLPLRRDDES EIYLSFLDKK SWCKNKFQVT NQVSVEDTYK ARYDVTILIN GLPLVQVELK RRGIDINEAF NQVKKRYRKQN YTGLFRYIQM FIISNGVETR YFSNNDSELL KSHMFYWSDK QNNRINTLQS FAESFMRPCQ LAKMISRYMI INETDRILMA MRPYQVYAVE ALIQQATETG NNGYVWHTTG SG
RM_EB_11	MAYQSEYALE NEMMNQLEQL GYERVTIRDN KQLLDNFRTI LNERHADKLE GNPLTDKEFQ RLLTMIDGKS IFESARILRD KLPLRRDDES EIYLSFLDKK SWCKNKFQVT NQVSVEDTYK ARYDVTILIN GLPLVQVELK RRGIDINEAF NQVKKRYRKQN YTGLFRYIQM FIISNGVETR YFSNNDSELL KSHMFYWSDK QNNRINTLQS FAESFMRPCQ LAKMISRYMI INETDRILMA MRPYQVYAVE ALIQQATETG NNGYVWHTTG SGKTLTSFKA SQILS

Table S3. Amino acid sequences for MS_{fus}, ½S and $M½S_{fus}$. The sequences derived from HsdS are underlined.

MS_{fus}
MSITEKQRQQ QAELHKKLWS IANDLRGNMD ASEFRNYILG LIFYRFLSEK AEQEYADALS GEDITYQEAW ADEEYREDLK
AELIDQVGYF IEPQDLFSAM IREIETQDFD IEHLATAIRK VETSTLGEES ENDFIGLFSD MDLSSTRLGN NVKERTALIS
KVMVNLDDLP FVHSDMEIDM LGDAYEFLIG RFAATAGKKA GEFYTPQQVS KILAKIVTDG KDKLRHVYDP TCGSGSLLLR
VGKETQVYRY FGQERNNTTY NLARMNMLLH DVRYENFDIR NDDTLENPAF LGHTFDAVIA NPPYSAKWTA DSKFENDERF
SGYGKLAPKS KADFAFIQHM VHYLDDEGTM AVVLPHGVLF RGAAEGVIRR YLIEEKNYLE AVIGLPANIF YGTSIPTCIL
VFKKCRQQDD NVLFIDASND FEKGKNQNHL SDAQVERIID TYKRKETIDK YSYSATLQEI ADNDYNLNIP RYVDTFEEEA
PIDLDQVQQD LKNIDKEIAE VEQEINAYLK ELGVLKDEMS NTQKKNVPEL RFPGFEGEWE EKKLGEFAGK VTQKNVDKKY
IETLTNSAEL GIISQKDYFD KEISNIDNIK KYYVVEENDF VYNPRMSNYA PFGPVNRNKL GKKGVMSPLY TVFKIQNIDL
NFIEFYFKSS KWYRFMALNG DSGARADRFS IKDRTFMEMP LHIPCMDEQI KIGQFFSKLD RQIELEEQKL ELLQQQKKGY
MQKIFSQELR FKDENGKDYP EWEETTIKEI AQINTGKKDT KDAITNGSYD FYVRSPIVYK INTFSYEGEA ILTVGDGVGV
GKVFHYVNGK FDYHQRVYKI SDFKNYYGLL LFYYFSQNFL KETKKYSAKT SVDSVRKDMI ANMKVPRPIY IEQKKIGQFI
KRVDNKTKIQ KQVIELLKQR KKSLLQKMFI PGGSHHHHHH

HsdM
MSITEKQRQQ QAELHKKLWS IANDLRGNMD ASEFRNYILG LIFYRFLSEK AEQEYADALS GEDITYQEAW ADEEYREDLK
AELIDQVGYF IEPQDLFSAM IREIETQDFD IEHLATAIRK VETSTLGEES ENDFIGLFSD MDLSSTRLGN NVKERTALIS
KVMVNLDDLP FVHSDMEIDM LGDAYEFLIG RFAATAGKKA GEFYTPQQVS KILAKIVTDG KDKLRHVYDP TCGSGSLLLR
VGKETQVYRY FGQERNNTTY NLARMNMLLH DVRYENFDIR NDDTLENPAF LGHTFDAVIA NPPYSAKWTA DSKFENDERF
SGYGKLAPKS KADFAFIQHM VHYLDDEGTM AVVLPHGVLF RGAAEGVIRR YLIEEKNYLE AVIGLPANIF YGTSIPTCIL
VFKKCRQQDD NVLFIDASND FEKGKNQNHL SDAQVERIID TYKRKETIDK YSYSATLQEI ADNDYNLNIP RYVDTFEEEA
PIDLDQVQQD LKNIDKEIAE VEQEINAYLK ELGVLKDE

½S
MS NTQKKNVPEL RFPGFEGEWE EKKLGEFAGK VTQKNVDKKY IETLTNSAEL GIISQKDYFD KEISNIDNIK KYYVVEENDF
VYNPRMSNYA PFGPVNRNKL GKKGVMSPLY TVFKIQNIDL NFIEFYFKSS KWYRFMALNG DSGARADRFS IKDRTFMEMP
LHIPCMDEQI KIGQFFSKLD RQIELEEQKL ELLQQQKKGY MQKIFSQELR FKDENGKGSH HHHHH

$M½S_{fus}$
MSITEKQRQQ QAELHKKLWS IANDLRGNMD ASEFRNYILG LIFYRFLSEK AEQEYADALS GEDITYQEAW ADEEYREDLK
AELIDQVGYF IEPQDLFSAM IREIETQDFD IEHLATAIRK VETSTLGEES ENDFIGLFSD MDLSSTRLGN NVKERTALIS
KVMVNLDDLP FVHSDMEIDM LGDAYEFLIG RFAATAGKKA GEFYTPQQVS KILAKIVTDG KDKLRHVYDP TCGSGSLLLR
VGKETQVYRY FGQERNNTTY NLARMNMLLH DVRYENFDIR NDDTLENPAF LGHTFDAVIA NPPYSAKWTA DSKFENDERF
SGYGKLAPKS KADFAFIQHM VHYLDDEGTM AVVLPHGVLF RGAAEGVIRR YLIEEKNYLE AVIGLPANIF YGTSIPTCIL
VFKKCRQQDD NVLFIDASND FEKGKNQNHL SDAQVERIID TYKRKETIDK YSYSATLQEI ADNDYNLNIP RYVDTFEEEA
PIDLDQVQQD LKNIDKEIAE VEQEINAYLK ELGVLKDEMS NTQKKNVPEL RFPGFEGEWE EKKLGEFAGK VTQKNVDKKY
IETLTNSAEL GIISQKDYFD KEISNIDNIK KYYVVEENDF VYNPRMSNYA PFGPVNRNKL GKKGVMSPLY TVFKIQNIDL
NFIEFYFKSS KWYRFMALNG DSGARADRFS IKDRTFMEMP LHIPCMDEQI KIGQFFSKLD RQIELEEQKL ELLQQQKKGY
MQKIFSQELR FKDENGKDGS HHHHHH

Table S4. Nucleotide sequences inserted into pUC19 to make the "E" series of plasmids for the DNA cleavage assay.

CLONE E1
GTGTTTCTCGAAAACAGGCAAAATCTTTAATGAAAGGGTTCTTGTTACTATGGGAATAGATATTATTTGCGCAATTGATCCCGGTGTGTCAG
CTGGTGGAATAGTGGTATATAAGCCGGGTAATAGTCTTATTACTATCCCAATGCCACGCACGGCAAAGGGTATTTTTAACGTGTTTCAAAAA
GTGAAGCGTTCCGGTAGCCCTGCAATATTCATTGAGCGTCTTTCGGTTCGTGGGGGTGACTCCGGAGGCGGGAAAGAATTTAGAATAGCAAC
TATGTTGGAGAACTACAACTACCTTGTATGTTGTGCGCTCGTTCTTGATATTCCTTTATTTCTGTGTGCGCCTATTTCGTGGCAAAGTGGTT
TAAATCTGAGGGAGAAAGGAGAGAAAGAGGAAAAGAAGGATAGAAAAGAAAAGTATCTGAATTATGCGATGAAGCAATTCCCGCTTGCAAAC
GTGAAATTATGGAATAGTGACGCTATATGTATTTTGCGCTTCGCACAGATGAAGATGATTTGTGATGTCGATTGGTTTTCAAGTAACATGCA
GAACGAAAACAGCACAGAAATAGCATTTTCTCCCCCTCTGTTGGACGATAGTATTAAATTCGTAGAAAGATATGGATTCAAAAGAAAACGAT
CTAAAAAACGCTCTAATTGAATCGGTGAAAGAATTGAGAAGCGCACAGAAGAGATTTGAGCGATTCGGGGAGAGATACAGAGAGAGAAAAGA
AAAGGCGGAAAAGAAAGTAGATGATATAATTTCTAAGCTGACGGACACGCAAATAAGTTTATTTTAATTTATTAACTGTTTGTCTATTGCTA
TGTTCGATTAAGTGCGTATATTTGCAGTGTAAAATTTGTCCGCCAACAAATTTAAAGATATTGCTTAATAGCGTTAATGCCTCGGTTCGTGT
TGTTTGGCGGCACACGAATCGGGGCATTTAATTTTAAACTGTAATGATATGGAAATTTCGGTGATTGATCAGAGAGAAGTTTTAGGAGTTGA
TTTTAAAATATATGGTACTTTTGATGATCCTTTGTTTTTGGCTAAAGATGTTGCTATTTGTATTGATTATGATTTGAGTAGCGTTAATAAAC
TGGTGAACCTTGTAGATGATAATGAAAAGGTACGGAACATTATTCCGACACTTGGAGGTAATCAAGAAGCTTGGTTTTTAACTGAAGATGGT
TTATACGAGGTGTTAATGCTAAGTAGAAAACCTATGGCAAAAGCATTTAAATCAAAAATAAAAGAGATATTGAAAGATATACGCAGACATGG
TATATATGCAACTGATAATGTTATAGATCAGATATTAAATAACCCAGATTTTGGAATAGGACTATTAACTAAGTTAAAGGAGGAAAGATCGG
CAAGAATAGAGGCGGAAAAGAAAAATGCAATACTTATGCACGTGAATAAAACTTATACATCTACGGAGATAGCGAAAGAGATAGGTTTAAAA
TCGGCAAACGAATTAAATAAAATACTCGAATCTAAGAAGATTCAGTTTAAAGTTAATGGTACATGGGTATTGTATTCTAAGTACTCTAATTG
CGGTTACGAAGAAGTGAAACAAGAAGTTCTTGATAGTGGAAGGGTTATATATCATAGACGTTTCACGCAGCTTGGGAGAGATTTTATATTAA
ACCTATTCAAGTGATATTCTAAACTAAAGTTAAATAACGGGTATTTCGGAAAGATTTACCCGTTTTTATTTGCGTTAATTTAAAGTTTTGCT
TTAATTTGCAGCGTAGAAATAAAAACAGTAGTAACAATAAAATCAATTAATTATGCAGGAAATTAACAAGAAATTAAGTGAACAGTCAGTAG
AAAAGGTTTTGGATAGACCGGAGTATAGAAAAGAGCTTTCTATTTATTGGGATGGCTTAAAAGAGCAACGGAAAAAGGCTTCGTTCGAAATA
TTGCATCACGGAGGTATTCCTAAAAGAATAACAATAGATAGAGTAGATAAAATGGATACGGATCAACTTATATCAGAATTTAAGCTGATACT
TGACAGAAAGAGTGAGTTGCCTGCAAGTCTGAGGTACTTTATTTCGGATGTGTGCGGAAAGGTATTTATTAGTTGGTTTACAAAAGTGATCG
AAGATGAAGCAAAAGAAAATAACGATACCCGGGAAGGTAACTAAGGACGGTAAGTTATCCATCTACATGGGCGAGCTTAACGAGTTTATGAA
GAACAACGCAGGGAAAAATATTATTGCGGAGTTTACGGTATTAGAACCGTCTGATTCTTCATCCTTGCGTGGATACTACTTTAAATACGTTG
TTCCCCAATTTCAGAAAGGGATGTGCGAAAATGGGTACAGGTGGAGCGAAGAAGAAACGGAGGCTTATATGCGTAGTATTTGCCCTATTACG
ATGGGTGAAGTTGTAGATGTTGAAACTGGTGAGTATAG

CLONE E2
GTTGAAACTGGTGAGTATAGAAAGGACTCAGTTAAAGTTACCGATTTAAGCAATAGCGAATTTGTCGAATACATAGAATTTTTAAAGCAGTT
TGCGGCAGAAGAATTTAGTATTTATATTGAAGAACCAAATAGATTTGTAAGATGAAAGAAAATGAAGAAATGACTTTAGAGGAAAAGTTCAA
TTTGATGTGCGAAGCATTAAGCATATCTCCGGAGAAAATTATATCACGGGATATTACACGTTATGTATCACTTCGAAGGAATTGCATTATCC
ATCAGCTTTACGCCTATAAAAATCACGGTTTACCCGAATTGATAGGTCGCACGAAGGTTTTAATTATGAAAGCGCATGAACGTTTTCAAGGC
GAATTAGATGTGAAAGATATGACAGCCGTAGAGTTTGTACGGCTTATAGACGAACGACTGCAAAAGTATATTGATGGCAAAGAAGATTAAGA
ATCTTGTTCTTGTTCATTGCACGGAGTGTAGGTTCAGTTCAGATCACCATAATTTGATTTGCTATTGCAAAAAGAGAGATAAAAAGTTATGC
AGTTGCCCGAACATTGGGCGGGTCTGTGAGTTTTACATTAAAAAATAAAGTATCATGTTAAAAGACAATTTTGAATTAAAGAGAGTTAAGTT
CTTGAATAACGGTTTAGAGGTTGATTACAATGATTGCCGTTTGGTTGATGGTGAAGAAACAAAGACGTTTCACAAGGTAAAATGCCCCGAAT
ATCCGCATAGAGATTTAGGAATTGCGGCAAATGAGCTTCGTTCATACATAGTTGAATTGATGGGAATAATGAATTTTAGGAACATCACCTAT
TTGTCTGATTTGGCAAAACAAGACAATGAGTTAAGTAGACAATTCGATGAATATTTTGAAACGCTTGCTACCCGTGTCGCCATTAGCGAGAT
AGTTTACGATCCCGAAAAGAATACAATCGTTTTCAAATATATTTTCGCAGGCGTAGATTTATCCCGGTTGAAAATGCAAACGAGCAAAATTA
TGTTGGACGGTGAAGGGTTGAAATTTGAAATAGCACTACAAGAAGATTTTGAAGCACTGAAAGATGAAATTTTCAAGTATCTTTTTGAGAAT
AAGCGTGCACAATTGGAGCTATTCGGTGAGACAGCAACGGCAGAACAGGACGATAGTTTGACGCCCGATGATGATTTAGAAGGTGACGATAC
GTTTTTTGATGATGAAGAAGCAGAGCAGCCGGAGTTGATCGAAGAAGATGTACACGATTGATACGTTTGAGGAAATAGATTATTGTTTAAGC
AGGGGGTATAACCCCTTGCTATTTAATAATAATTTCGATATTGAGCCTAAAACAAGGTATGAATATTTAAAACGGATGTTCGGAGAGGGTCA
CGGACAGAGGGGAAATGAACGTTTCTTCCGGTATATGTGGGATATTAAGCCTCACTATTGTGAAGAATGTTTAAAGCCGTTAGCAGGGTACT
CAGCCGTTTATATTTCGCATATTATAACGAGGGGATCGAACCCAATGATTGCGCATGATCCTCGTAACATAAACATACTTTGTTTCAATTGC
CATAATCGTTGGGAGCACGCTAACACCCGGAAGGGAATGCGGATATATCAAAGTAATTTAGAAAAAATAAAAGTCCTCAAAAGGGACAGTTT
AAAACTGCAAAAGAAATGAAATTAGTAAAATTTGAACTTGTATCTGGAAATGAAATTATGATTAACCCTAAATCTGTGGAATCAATAGTTAG
ATATACAGATGATTCGGTGTATATTAACACAGTAGGTGTAGATATGCCGTATAGGGTTAAAGGTTCAATTGAAGATGTCAATAAAGCACTAA
GCGAAGGTAGCAAGATTGATTCAATAGCAGGACTTATGGTTATCGTCTTTATTGGAATTTACATATTATCAACATTAGCAAATTTATTATCG
TAATGAACTTAAACAAAATCGAATTGATCGGGCGTGTTTGCGCTGATCCGCAAGTAAAAACCTTCGATAACGGAGGGAAAGTATGTAATCTT
TCTATCGCAACGAACGAAAGGGCATATAAAACGAGTAACGGTATCGAAGTTCCGGAAAAAACAGACTTTCATAATGTAACATTCAAAGGTAA
ATTGGCTGAGATTTGCGGGCAGTATGTTACCAAGGGAATGGAGTTATACGTAGAAGGTAGTTTGCACTATCGAAAATATACCGACTCCAATA
ACGTTGAAAGAACTATTTCTGAGATCGTTGTTAGGTCTATGCAGATGGGAAGAAAAGCAGGTGAGGGAAACCAGCCGACAACCGGAGGCAAC
GGAAACCAACAGCCGACAACCGGAGGTTATAGCGGTCAACAGCAACCGCCTCAGCAGATGTTTACGCAAAATGATGATTTGCCGTTTTAATG
TAGTTTATAAATTGGGGATGTATATTGCATCCCCTTTTTTGTTAAATACGTGTTAAAACTTAAAGTTTCGCTTGTAATATC

CLONE E3
CTTAAAGTTTCGCTTGTAATATCAAATTAAGCCCTTATATTTGCAGTGTCAAAAGGAAACAAAGTACTAACATTTAAAAATAAATATTATGG
TAACAATGACATCAAAACAATTTTGTGAGAGAATGTATGCAATGTATAAATTACTTGGTGGGAGTGAGTCCGGATGTGCTCAATGTTCAAAC
GATAGATTTTCTTGCGGATATGGAAAGGAGAATACGGTTTTAACCAATGCACTTATGAAAGCGTGCGATAATCACAAAGTTCCTTATAAGAT
AGAAGCAAACGAATATTGTATCAATTTCGTAGTAGAATTTAAATAATAATAGCGGTAGAAATACCGCTTTAAACTTATAGTTATGGAAAAAA
GAAGATTGTCCGGTCAATACAAAATAGCGATGTGCAAAAATAGAGGAAACGATACATTTGCCGGAACTGTTGGAATAAGAACAGGTTTTATG
TATCAGTGCGGTGCGTATCAGTATTTTACTTATTGGGAGAATGACAATAAAATATCGGTTACTGAATCAAGTACAGGTTTTCGTGTAATGTC
TTTGGATGTTGAAAAGGGAGAAACTCCTAAAACTGCGCATGATAGGATAGTTGATAAGTTGAAGGGTTTTGATCCATCTTTAGAAAACTGGA
ATAGTGCTAAAGAGATGATGAAGAAATATAATATTCCCTATCCTCTTAATGAATGGATAATAGGGCTAAAAGACATAAACCATGAATGAAGA
GGTAGAGAAAGCAAGATCGGTAAGTAACGAGGTTATTTCGGAAACTATCAGAAAATCGACTGATAATATAAAGGCAATGGAGGACGATTTCA
GATTAGTAAGAAAGAAGTTGCGGAAAATTGGCGATCGAATAAAATTTGAGAGAAAGAAACTTGATATATACAACGAAGAAATAAAAAGGAGG
GTTAAGTATGGAATTTGGTAACTTACTGTTATATAGATTGGGGTTCAACCGTGAAATGTTGGAAGATAAACTTTCAGAAATATCCGCTAAGG
AGAAAGAGATAAGAGTCCTAAAGAAAGAAGTTTCCGGTATAATGGAACACATATCAAAATTGGAAAGTGCGTTAAATCATGGAGAGCATTAT
TATTGCGGTGCTTGCTGCTATCTTGAAAGTAAATGTAATAAGGGAAAATATAAGTGTCTTGAAACCGGAGAATACAAGAAATATCACTGTAA
GGCGTGTGAGAAATTTAGAGATTTACCATTTTAATAACTAATTATAAATTAAATATTATGATTGATTTTAATCAAAAAAGTATCTCTTTAGC
TAAAGAGTGTACAGAACAACATGAAAGAATGAAGGCAAAAGGTTTTTATGAATCAGATGTTTTTGAGTGTAAAAAATGGGCGTTGATAGTGT

CTGAGTTCTGCGAAGCTATGGAGGCTGAAAGAAAAGGCAGAGTTATAGAAAACGATGTATATGACTTTGCTATGAATAGGAAATCAAAGGTA
GGCTTTGAGTCGTATTTCAAGAAATGGATAAAGGATACAGTTAGTGATGAACTCGCAGACGTGTTTATCCGGTGTATGGACGCAATAGGACA
TTCTATTGATAAAATTGCGTGTCCTACCGAAATTTTTGTTTTTCAAAGTATGGTTAGCGATCATTTCAATAGGTTATTGTATCTTGAAAAAT
CTATTTCATCAATTGTTTATTATGCCATTCAATTTGTGCCGAAATCTGTATTTGGCAAATCGTGCATTACCGAGTATACTAACATGATGGCA
ATAACCATTGCAGCCGCAAAGCTTTATAACATAGACCTATCTAAAGCAATAGAGGCAAAGATAAGATATAACGAGTTGAGAGGTAAAAAATA
TGGGAAATAATATTAATTCAATTGATATGGAAGAAAAAATTATTGATTTAGCAAGAAGAAGCGTTTATTATGGTGATCCGGAAGGTTACCAA
GTTGGGGGAGGCCATTACAAGACATCCGGCATGCAGCTTTCTGAATTTTTAGAAAGGAATAAAGTTGGTTTCTTGGAGGGGAACGCAATGAA
ATATGTGTTTAGGCACGATAAGAAGAACAAAGAAGAAGATTTGCTAAAGGCTATTCAGTATATCAAATTGATTCTAAAATACAAATATGGTA
AATTCTTAGTAGGTGATATTCTGTTGAGTGAAGAAGAATATAGAAAACTGGATGAGCTTATTGAGAAGCAAAATACGATTGAATTTGATACT
ACTTTTATCAGAAATGCGTTAAAAACCGCATCAATCAGAGTAAACAAAATATCGGTAGAAAAAGCAAACTTGTATGTTGCAAAGCTAAGAGA
GGTTAAAGCCGAATATATCGAAAATTTCGTTTTGTCGGATATACAAAAACACAGGCTTTTAGATATGGGTCTACAATGGATAAGAAATAGCA
CGTATTGCCTTAATTTTACGGCAAAAAATGGAGATATGATATGTGTTAAGCCTGGGC

CLONE E4

GATATGTGTTAAGCCTGGGCAATATATTGTGTTGTTTGAAGAAGGTAAATATCAAGTGTTCTCAAAAGGAAGGTTTGAATTTCTTTTTCAAC
CGAAATACTAATAAAAATAAATAATGATAGGTCACGTTGCAAATATAGCAGCGTGACTTTATTTTTATATTATCTATAATAGTGTTATTTTT
GCGCATATTGAAAGATTATATAATTTGTAGTACAATATACAGAATAGAAATTATAACTTAAAAATACGTCTTAAAATGGATAAAAAAATAGG
TTCAATGAAAAGAGGGCAGGGAAGGCATAGCCGGACGGACGAACAGACTGAAAGAGACCGTTCCTTTGCCTCTGATTTGTTTTTGAAAGGTT
ATTCTTATAGAAGAATAGCGGAAGCGATTAACGAGCGAAATAAGGCGGATGAAGTGCCGTATACCGTGACTTATCAAACAGTGTATAATGAT
ATTCAGTTTTGCCTGACTCAGTGGAAAAGAGAACAGTTCGATAATATAGATCAGTATATTACGCAGGAACTCCAATCTTTGGATAATGTAGC
TCGTGAAGCGTGGGAAGAGTGGGAAAAGTCTAAGCGTCCCAAATGTAAGACAAAGTATATTTTAGGGAAGGCCAAGGAGGTGCAAAAGGAAA
CAACAACGGGTGATCCTTCTTTCTTGAATGTAGTTCTCAACGTGCAGCAAAGAAAAGCAAGGTTATTGGGATATGATTCTCCGCTATGTATA
AACTTGGTAGGAGACAAAGAAAAAGAAAAGCCTAAATACGATTTTTCGGATGTTCCGGAGGACGTTTTAGAACAATTGGCAGATTCTTTGCA
AAATACGGAGGGTAAAAAGTGAAAAAAGTAAATGAAATACCACCTATTGAGATTGTGAAGCATGTTGCGAGGAAGAAGTTTAAGAACTATGC
TAAATTCATAGATGATAAAATAGTTCTGAGTCAGTTTCACAAAACTTACTACGAGATTCTCGATAGGTTTGCACATGGTAAGATCAAAAAAT
TGATTGTTACCGTTCCGCCTCAAACTGGAAAATCAGAGGGTAGTAGTAGAAAGCTACCTTCTTTCCTTTTGGGGCTTAACCCGTCTTTAAAG
ATATTGATCGGTTCTTATGCCGCATCACTCGCAGAGGGGTTTAATAAGGATGTACAAAGAATAATGGATACACCGGAGTATAAAAGCCTATT
CCCCGACACCCGAATAATGGGAGAGGAAAAAAAATCGAGGTATCAAGCGTTTGCGAGAAACTCAAAAATGACGGAAACAATCGGAAAGGGTG
GGTATGTTATATCAGTTGGACGTAATGGTAGTTTGACGGGTAAATCTGTTGATATAGCCATATTGGACGACTTGTATAAGGATCATATGGAG
GCAAATTCTCCGATTATCCGGGAAGCTGCTTGGAAATGGTACACCACAGTTGTAACCACCCGTCTACACAATAACAGTCAACAACTTATTGT
GTTTACGAGATGGCACAAGGACGATTTAATAGGGAGGATCGAAGATAAGGAGAATGTTATCACTGTTGAGAAGTGGGAAGATTTGGATAATA
TACCGGATGGCGCATGGGTTAAAATAAACTTTCCTGCTTTAAAGGTGGGAGAACCAACAGAGATTGACCCACGTTTACCGGGTGAAGCACTT
TGGGAAGAAAAACATAGCGCTAAGAAATTGAACGCACAAAGAGAACTTGATAGAAATGAATTTGAATGTTTGAATCAAGGAAACCCGGGTAG
TGCTGAGGGGACTCTATACGGTAACTTTAAAACGTATACCGATAAAAACGATTTTGGTGTGTTGGTCGGAAGGGGTAACTATACAGACTGTG
CGGATACTGGTAGCGACTACCTTTGTTCAATTTGCTATGATAAATACCAGTCAAAAGAAGCGGTTTGGAATGAAAAGGAAAGGAGGTATAAG
CATCTTATTTTCTGCCTTGTAACGGATATTGTTTATACTACCGCACCAATCGAAGAAACGCAGGTTAGTGTTCCTAACATGTTGAATATGAA
TAGTACAGATTACGCATACATAGAGAGTAATAACGGAGGGCGATCCTTCGCTGTAAACATCAGTCCAAGAACTAAGACTGAAATTAATTGGT
TCTGTCAAAGGTTAAATAAAGAGGCTCGTATATTGTCGAACGCTGCAAACGTTACTCAATCTATTGTTATGCCGTACGGGTGGGAGTCACGT
TTCCCGAAATTCCACGAACATATAACGAATTACCTTCGTGAATTTTCCGCTAACAAACATGATGATGCGGCAGACGTTTTAACTGGTATAGT
CGAGAAAGAAGTTATTCCAACTATATATCAAAAAAGAAGAGGAATAAGGGTTATAAACTGATAAAGTAGGAAAATGTATCAGACTTTCAAGT
TTATACGGTATATTTGCAAAGTAAAATCAATTGTTTAACTAAATTTTTATAATTATGTTGTATTGTAATTGTCC

CLONE E5

TTATGTTGTATTGTAATTGTCCTTTAGGCGAAGCAATTCCGGATATTCCCGCATTTAGCTGTCCAGACAATTTCGGGCAAGTTCAAAAACTT
GCTTTTCAGAGACTCGAAAAAACGGCAGGAACTGCAAATACTATGACTGCCGAAAGTATCGTAAAGTTGGCTACATGGACTCCTTTACTGTC
AGCAAAAGACGGTACTAAAGTAGTAGTTACGCCTTATATTTACGAGCCGACAGTAGAGGCGGGAGCTGCCCTTACTTATGGAGGTGGAAACG
CAACTCCCGGAGGTATTGTAGAAATTTTGGGGTCGGAGTCGACACCGTTTACGGCTTCGTTCAAGAAGTTGCCGCAAACCATTATCAAAGCT
ATGAAAGCGTTGATGTGTGAAGCGGGTCAAATCGGTGTGTTCCTTATCAACGGTAACGGACAGATTGCTTGTGATAAGACGGGTAATAATTT
GCACGGTTTCCCTGTTTGGTCGCTGTTTATCGGTGATAAGACTATCGGAGGTTTAGAAGCTCCGGATAGCAATGCTATTACGTGGAACTTCA
TCCCTAATTGGTCGGACAACTTCACTATCGTGAAACCTGAGTTTAACCCTCTGACTCAGTTAGTGCCTTCTACGGGTATAGGCGGATGATAG
CTAAAAAAACGTATATTTCCCTCAGTTGTGAAGAACTGGGGGAAACTCGTTTATTCGATATTGAACACGCTGAGAGACTTTTGGGAATGGTT
AATAATGGAGGGTGGCATATACCGGAGGACTCAGAATTTAAATTAAACGAAAATGGGAAAATCATTAGACGAAATAAGGGAGATATACAGAC
ATCCGGAGGGGATCAGTCAAATAGCGAAAGCAAAGGAACACGAAGAAAGAATAGCGTTTCACACACGGGTGAGAACGAGTGATGATCGTAAT
AAGCCAGTAATTGACTTTCTTTCTAAGGTTAAGACGTGGATAGCGAAAGATAAATATGATATTTTCCTATCTATGTTCCATTTCCCGGTTAA
AACAAATGGTGTTACTTCTGAGATATTCGACAAACTGAGCCGTGTTTTCGATGGTAGGAATCCGGTTTATAACTATCAGTTTAAATCATCTG
AGGATCGGGATGACTGGGAGTATTACCGAAAGGATGTTTTAAAAGAACCTTCGGTTTGGAGTACGGACGGTTGGGATAATTTCAAGCATAGA
ATTAACTCTGTTTTGGTCGTTGATATGCCGGAGGTACAGGTAGGAGAAAAACCAGAGCCGTATTTTTTTTGGTTGCCTATTGCAAACGTACT
TTCTTATCGCACATGTGGGAAAGACTGTAATTTGATGGCTTATATCATGTACGTAACGGACGAAAATAAGATCGTCTATATTGATGAAGAAC
GTTATGTAAGATTTGATAAAACGAGGGGAAACGACTTGATTTTAGAGGTAGACAATATGCACGATTTGGGCTATTGTCCGGCTCGTTTCTTT
TGGTCTGACTCTATATCATTGAGTGAACCCGACATTAAAATAAGCCCTATAACGAGCGAACTCGACTCTTTCGACTGGTATCTTTATTATTC
CACTGCAAAGAAGCATTTAGATTTATACGCGTCTTATCCGATTTATTCCGGTTATGAACGTGATTGTCACTATGAGTCACACGATGGCAAAG
AACGGTGCGATGATGGTTTTTTAAAGAACGAAAAAAACGAGTGGATAACAGGTGAGGACGGAAAACCGATGGCGTGCCCGATTTGCTCAAGC
AAGCGGTTGCGGGGTGCAGGCTCTTATGTTGAGATACCTATCCCGGACGAAATGCACAACGTCCCCGACTTGAAAAACCCGATCACTATGCT
ATCCGCTGATACCGGATCACTCGAATATAACGTAAACGAGGAAAAGAGGCTGAGAGAGGAACTTGTAAGATCGGTAACCGGTGGAGAAGGGG
AATTAAACAGGTCTGAGGCTATTAACGAAAAGCAAGTTAAAGCGGGTTTTGAGTCCTTGACTACTAAACTAAACAGAATCAAACGAGGCTTC
GAGGAAGCGCAAACATTCGTAGACTCTACTATCTGTTTACTCCGTTATGGTGATAGCTTTGTTTCTTGCAATATTAACTACGGGACTGAGTT
CTATATCTATACACCGGAAGAGCTTTCAGAGCGTTATAAGATCATGAAGGAAACCGGAGCGTCCGAGGCGGAACTTGATGCACTGAGGCAAC
AGATCATCGAAACGGAGTATCGGAACGACCCTACACAGATGCAAAGGTTATTAATCCTTAACGAGATAGAGCCTTATTCACACTTAACGAGA
GAAGAAGCGGTAAATCTGTATAAAGAAAACGTTATAAGTGAGGAAGATTTGCGAGTTAAATTAAACCTTACTACATTTGTGCGTAGATTTGA
AAGGGAGAACATGAATATCATTGAGTTCGGTTCTGCACTTGAC

CLONE E6

GAGTTCGGTTCTGCACTTGACTATAAAAAGAAAATTGAAATAATTATTAATACTTTAAAAAAGTACGCAAATGGTTTACAGAACGGATCAGT
TAGATCAACTGAATGAAAGTAATTACGTTTGCCCGAAGGATGAAGTTAAATTGTATCACGTTATTCAAGAAGTGAAAGAGTTTAACCCGAAA
ACAGGGCAAAGAATCAGCGTCCCGGTGTTGCAAAAATACAAGCGAAAGACTTTTGAACTTGATATTTTGCCGAGACTGCCAAGATTGGGTTA
TACATTGAGAGTTGTTTTCGACCCGGTTAAATATGAATCTACAATTTCAGAGGCAAGACGAGCCGCAGAACTGGCAGCGAGAGCCGAGGCAA
AAATGAAGGCAGACGAAGAACTGAGAGAGCAAATTAGACGTGAAGAGGCTGCAAAACTTCGTGCGGAATTGAAGAAACAAAAAGAGAAAGGA
GAAAAGTAATGTTAACAGTAGAATTGCTTAGGCAGAATAAAGCGTTATCGGAGCTATCGGATGAAGTTCTTAACGCTATTTCGGAGCTTTCA
AAAAACGATGAAGCGCAGACGGTTGCGGCAAAGGTGAGAGAAGCCGAAAACAGTATTGCTTCTCAAATGAAAGAGGCTTTTGGTATTGAGGG

TGTAACCGATCTCGATTTGAAAACCGCAATTGAGTTTGGCAAAACAAAACTTTCTAAATCTGATACCTCAGCTTTTGAAAAACAGATTAACG<br>ATCTGAAAGAAGAACTAAAAGCTGAGAAAGCTAAAAAGGGAGGAGACCGGGATACTGATAAAATCAATCAGCTTACTGCCGAACTAAACGAC<br>ACCAAGCAAAAATTTGCTGAGTTGAACAACCAACTTTCAGAGAAGGAAAAGGAGTTTAACGGTAAGTTGAACGATTACAAGATCACTTCTTA<br>CATTTCAAGCGCTATGCAGGGGATGAAGTTTAAGAAAGATATTTCAGAGCCAGTTCTAAACGTTGTGAAGCAACAGGCGGTTAACTTGCTTA<br>AAACTCAATTCTCACCCACTTTGCAGGGTGACGAAGGTTCTGAAAGTCTTATTTTCATGAAAGACGGTGTACCTTACAACAACCCTGCAAAC<br>AGTCTGAAACCGTTTACCGCATCAGAACTTCTGTCTCAACAGTTTGAACAGTTCGGTGTGCTTGACAAAGGTAGACAGGCAGGAGGTGCGGG<br>TAGTTCCGGAGGCGGACAGGGTAACGGTAGCTTGCTTGATTTAAGCGGTTGCAAAACCAAAGTAGAGGCAAACAAGGTTGCGCAGGAGTATT<br>TAGCTAAGAAAGGTTATACAAGCGAGTCGGAAGAGTATCAAACGGAGCTTGATAAAATTTGGGTTGAAAACAAGATCGCAGATTTGCCAACA<br>GAATAACTAAAGAGGGGGTTAACCCCTCACAATATAAACTTTAAAACAATAGATTTATGTCGTTAATTGCTACAAGAACACAGGAGTTCAGA<br>TTAAAGAACCCTAACATTGACAAAAACATGGCTCGCATGACCGAATGGGGTGCGTATGACTTCTTTTTGTCTCAAACAAATGCGATGGACTC<br>AATGCTTTCCGATGAAACTAAGCGTAGAGCGTTCGCATCTATGGGAAGCGATATTAAGATTCCCGTAATTGATTACGATAAAAACGTAACGG<br>TGTCACACGCTCGCACATGCGTTATCGCAGATGCGGAGAACATTTCTCGTTTGATCGGTGTAACTTGGAAGACCTATGCTTTCGGTTTCACT<br>ATGACACCGAACATGTATTCAAACAACGAAATCGATTACCAACAGGACTGGAACAGAAAGCTACAAAAGCACATCCGTAAGTTCATGGATAC<br>CGTTGATAAGGACGCTATTGCGGCTTTGGAGGCAAACAAAACGCAAGTGTTCGGAAACTTGCTGTATTACACAAAAGAAGGTAATGATGTAC<br>AGGTGAAATTCACTCAGCGCAACGACATCCTCAGCGATTTGCACCCGATGTTCCGTGCAAACGACTATTCCGGTCAACTTCATATCATTGGC<br>GACACTGGTGTAGACTCAATGTTGCGTAAACTGGAACAGCACGGTTTGTACAATGACGTTAACAAACAGTTGGAGTATGCAAACAAAGTGTT<br>CCATTTCACCAACAACATGACTTTAGAGCCGGAAAACTTCGCTCAGATGTATGCTGTTGAATCGGGCAACGTTGGTTTGTTGACCCGTGTAG<br>ACCGTGCAGCCTACAACAACACTAAGTCGGGCACGCATGAATTTGGAAAAGTTGTTCTTCCTTATTTCGGTAAAGAGGTCGGAACACACTAC<br>TACGAAGAGGTGGGCGATCAGTCGGCTATCGCAGGCGCAGCAACTGCCGACATGACTTGTGACGTTAAACACTTCTACGGTTTCTCAGTAGA<br>TATTGCTTTCGTAGTAGCTTTTAACTCCGATACTTCAACAATCGCCAACCCGATTATGAAGATC
CLONE E7<br>CGCCAACCCGATTATGAAGATCGAAGTAAACAAAGAAAATTCGCAGTTTGGAGGTACTCCGGTATTTATTACCAATGCTGAACAGATCGGTG<br>GAGGTTCTCCGGCTGGCGAATTATCGGTTAACCTTGCTAAAATCGGAGGTAGCCCGGTTGCTGAATCTGCTTTGAAAGTAGATTTGGATAAA<br>GTCAAAGGTGCAGCGGTTTCGGCTACTGGTGGCGTAGTTGATGTTAAAGTCAATGCGCAGGCTGCAAATCTTAATGTTGAGGTGAAGAACTC<br>TGATAGCGCACCTGTACCAACAAAAACTGTTGGCGGAGCGTAACGAGAAAGTAAACTAAGTATTAACAAAGGGAGGGGGACAAAATCCCTTC<br>CCTTTTTTTATTTATAACCATGTACAGATTAAAGGATATACAAAAAGAACTTGCCACGCTCGTAGGATGGCGGCAGTCGTACGATAGAGACG<br>CTAAGATAGACGAAAGTTTAACGGTGTCCGATAGTGGTGTTATGTTTCAAGACGTTCACCCGCTTGTGACGCTAAGAAACATTGAATCTATT<br>ATGCCACTTGATTACTATTTACGTTATCCGGAGTATCGGGATACCGACACTTATAAGCCGGGTGACAAGGTAGTTTACGGCAAGGACGTGTT<br>AACGCTTCGTCCGGACGTATGGGAGGCAATAACAGAGAATGTTGGTGTAGAGCCTTCAGAGGGTGATAACTGGAAACGGTACAATCCACTAA<br>GCGATTATTTGCGTGAATTGAACGAAAGAGCGATCACCAATACCGTTACTCGCTTCATCAATGAAAAGTTGATTGCAGGGGAAACAAAGACG<br>CTTTTGGAGCGTACAAACTTCTTCGATGGTTCGGGGAAGATAAATAACGAGATTGACCCTACCGATAGTATTGTAGGATATGAAATATTGCC<br>AGTCCGTTCTATGGGAGTAACAGCCAAGATCGAGAAGATAGGTTTGCAGTTTAACAAGCCGGGAAGGGTAAGACTTTACCTTATGCACACCT<br>CACAGGTAGACCCGATTAAGACGTTTGATTTGAATTATACTAAAAATGGTTCTTATCAATGGTTTGATGTCGGTAACGATGTGTTACTCCCT<br>TATATGTCTGAGGAAACCTCACCCGGTGGCTTGTGGTACTTGTGTTACGATCAAAAAGAATTGCCGTTGGGGATGTATGCTATAAACGTCTC<br>TAAGGACTTTTCACGTGACCCGTGCGGTACTTGTAATATCGGAAGCGTGCAGGCGTGGAGAGAGCTAACAAAGTATATCAGAGTGTCACCGT<br>ATAGAGTTGACTCTACGCAGTCGGAGGATGGCGTAAAGATGTGGAATATAGAAATGAACATGTATACGTCTGCAATCTGCTACGGTTTAAAT<br>GTTCAATTGTCGGTAGGATGTGATATAACTGACTTTATCATTCAGTCTAAGTATGCCTTCACGCATGCCGTTTCTCTGCAAATGGCTTCTTA<br>TGTGCTGCGAGAGCTTGCATTAAATCCGAACGTCCGGCAAAATGCCAACCAATTGAATATCGACCGTGAAACACTATTGTACGAAGTTGACG<br>GAAATTCACAAGGGCGTGCGCAGGGTATCGGATACGAACTAAAGAAGGCTTTTGAGGCTCTTTCTATTGATACAAAAGGGATGGATAGAATA<br>TGCCTTTCTTGCCGGAACAACGGGATAAGATTTAAAGCAACATGATAAGCGGTCTAATAGATAAGTTTAAAAAGGTAGGTGAGGAACTCGAC<br>ACCGGAGAAATAGCAAAAAAGATTGTGCGTGACAATGATAATATACTTATTGACATGAACGCACAGGATCAGCTATACGCAAAGGGTGTTAA<br>CCGTTTGGGCGTTCGTATAGATGAATACCAACCCTACCGACCCTTAACTATAAAGGTCAAAATAGAAAAGAGGCAACCGTACGACCGGGTGA<br>CACTAAAAGACACAGGAGAGTTTTACGACTCTTTTTATGTTGAGACAGCAGAAGATCGGTTTTACATAAAAGCCTCAGATGAAAAAACTAAT<br>TGGCTTATCAAAAAATACGGTGCTGAGATTTTCGGGTTAACAAATGATTCACTTGCTGAGTTTATTAACGATTATGTGAAAGACGAAGCATA<br>TAACAGAATAAAGGAGATATTAAATGAACGATAGGGCTATAATTAGACCAAATGCGACACTTTTCGATAAAACGATAGCCGATGTACAGGTA<br>AGCCTAACAAAATCGCTTAAATGGCTTAATTTCGCTTTCGGGAACGTGGTTAAATTGGTAGAGAGAAACGAGAGGGGGAAATTTGTTACCCC<br>ATCAGTGTATTTTAAGGGAAATGATTATTTGCGCTTAGAGCCGGACGATAAGCGGGGCAACGTTTGTTTTTTCTATATGCACGACTCACAAG<br>ATTATGAAGGGGGAGACTCTTTGTCTGGCTTTGGCGATCTGAGGGGG
CLONE E8<br>GGCTTTGGCGATCTGAGGGGGACGGTTAGCATTATCTTTTGGTTCGATACCCGTAAAATCCCGGGAGCAGAATATTACAACGTGGAGTTTGT<br>AAAGTCAGAAATACTGAGAGCCTTAACGCACGAACTTTATCTGCCATCCGGTGATATACAGGTGAGAAAGATATTCCACGACGCCAACAACG<br>TATACAAGGAGTTTTCTATCCAAAAGACGGAGAATCAATACTACGTTTATCCCTATGCGTGTTTGCGGTTTGAGTGTGATATTCATTGCGAA<br>GAAGGGTGTTATTAAAGGGGGAGTTTCCCCCTTTTTGTGTTAAATACATGTTAAAACTTAAAGTTTCGCTTGCAATATTAAATAAAGTCCTT<br>ATATTTGCAATGTCAAACAACGAAAGACCCCACAATCTAACCAAGACGCAAAAAGATTGTTGAAAGATTAAATTCATAAGAGTAGAAAATAA<br>GCAACGGTATCTACGAAGGGTTAAATGAAGGTTCGGTATCCGATTAAATGAAGCTATAAAGCCTAAATCTTTCGAAGTATGACAAATTATGT<br>AGTAACAATTAAAAATCAAGATTATGACAGCAAGTTTATTTATTCAGAGAACAGTTGAAAAATTTATTATGATGGAATTTGTTAAGGGAAAC<br>ATGGATACCAAAGAACAAGTTGATACAATGATAGAAGTTATCAAAAGAAAGTTAGACTTTTCGCATGATGAAGCATGTGATTTTATAAGAAA<br>AGCGATCGGAATAAACGAATAACTTTAATTATTGACAGGTGGGGATAATACCCCACTTCCTAAAATAAAAGCCATGAAAGTAGATAAGTATC<br>TAAAGAGCCACAAGGCAAACGAATTTTACGTGAAAAAGTGTAGAGGTTATTATCTCGTTATGGATAGCTATGATAAAAGTTTGGCGTCTATG<br>GAGGTTACAGAAGAAGAAGCTAAAAAGGTAGCCGAGCAACTTAACAAGATTCGCAACGAAAGATTGAATCTAACCGTTAAATAATAGTTAAA<br>TATTAAATTTAAACGTGCAATATTAAATAAAGTCCTTATATTTGCAGTGTCAAAAGGAAACAAATTACTAACAATTAAAACCCAAAGTTATG<br>AAAAGATATTTTGTAAACGGAAAAGAGATAAGCGAACAAAAAGCAAAAGAGATTGAAGCTAATAATAAAAAGTATATGGAAAGCAATGACCT<br>TTCTCTTTGGGCGAAATGTGAATTTATAACAGTTATTGTAAAGTAAAACAAGTGGGGGTAACACCCCACATAAAAAAAATAAATATATGACT<br>ACTTACATTTATAAAGGACAAAAGATAAGCCACTCCAAAATATTATCCCTATTGCGTAGTGCAGGCATTTACGGAGGAAACAAACTATCATA<br>TTATGAAGTTTTAGTTAAAGCTGCCGAAAACGGCAACGAAAGAGCCGCATATATTTTGAGAGACTTAAAAGTGATATAATAACCGTGGGAAA<br>CCACACAAATTTTAAAGGTATGTTTAACAAGGAAAGAATTGAGAATTTAGAAAAGAGAGTTAAGGAACTGGAAAGAATAGAAGGTATTTCAA<br>AACTTTCGGAAGATGTATCTAAAAGGTTGATACATGCTTTAATTGACTCTAACAACGAAGTAGTGAAAAGAGTTTTTGATGATGTGTGTGTT<br>CGTTCGGTATATGGTACATCTGTGTTTCAGCCACCTAAAGCCGGAGACATTCACGCAAGTAATGCGGTGTTTAACGGTATACCCGATTCTTT<br>AAAGAAGGTAGAAGAGGATAAGGCAGAAGCACCTACCATTGCAAGCGTGTTGGAAAAAGCAAGAAGGAATACGATTGCGATACAGGAGCTTT<br>TAAAACGAACAGGATGTTCGAACGTGAACGAAGTAATAAGCAAGTTTGAACTTGGGGTTTCTTTCAAAAAGATGTATGATGAAGAAGCACGC<br>AAAAGGAAAGAGCTTGCAATACAAAGAGATCGTTTAGAAAGTGAAATGATGTGCAAAATACAGGACTTAGAATCAAGAAGAGAAGAGTTGTT<br>AACAGCAACAGGGTGTGCAAACTTTAGCGACTTGAAAAATAAGTTCTTATGTAAGGATATAAAGAGGGATGAACTAATAGAACAAATAAAAG<br>AGCTTGCACGGCAAAGAGATCTTTTATATCGTGATTTAAGGAATCAAATAGCTAAACTTTCTGATAAAAACCAATCTTTGATGCAAAGTGAA<br>AAAAGCCTTATTTGCAAACTTGCAGACAAGAACGAGGAATTAAAGAGAGTGGAAGAACTTTCAGACGGTAGATATAAAGAAGTCGTTTGGAT<br>TCGTGGCGAACTGAAAAATCAAGAACAGGCGGTAGAAAAACTAAAAGACGAAAACAAACAGCTTAAATATGCTAATTCGAAAATGGCAAAAA<br>GAACGGTTGATTCTATTTGTTCAGAATCGGATATTGCGGTAGGATATTCAAATTTGCAAAAGAGATGTAAGGATTTGG

CLONE E9
GCAAAAGAGATGTAAGGATTTGGAAAGAGATAAAAAATCATTGCTTAATTCCGAGAGAGAATTACAAAAACAAGTATTTGACCTTTCGAGAG
ATAAAAAATACTTGGAGATGGCAAACACTGCACTCCTGCAAGAAACTCGTAAAATTAGAGAGTCTTTGAACGAAAGAATTAAGAAGCTAAGA
CAGAGACTTAAAAAATCGTCTATCCGTTACAGAGACTTAAAAGAAATCATTTCGCACAACGGTTTGAAATCAGTATAACAATAATAGCCGGG
ATATTATCCCGGCACAATATTAAAAGATATGTTGAAGGTAGATATAAGAAATAAGATATTAAAACCCAAAATAGGTGAGATTATAACCGTAA
TAGGAGATATGTATACCACGGTAGCGGAAACAATACCGTATAAAGGTGAGGGGTGTGAAAAATGTGCTTTTCATGATTCCGGAAAGGTAGGG
GGTGATTGTGGTGATTTTGTGGCATGTTCTAAACTTACCCGTGAAGATGATGTTATGTTTAAACTAATAGAAAGAAAGAGAACTAAGGAGGT
TGAAAATGAATAGAATATCTTTGTATGATAGAGATAGATTCGTACCGAAAGAGGGTGAAGTGTTTTTTGCAGAAGTTCCGGGAAAGGGAATA
GACCGGAAGGTAGAGGCGGTATTATTGAAAGACAATAGCGGTTGTAAGAATTGCGCATTTTTTAAAGGAGAATTAAAAGACTTGTGTATGCA
AATAAACTGCCTTAATCGTGGTAGGCAATTAATTTTTAAGAGGGTGAGAAGATGAAGAGATTTAAAAGAGTAGAACTGTATGACACCTTCAC
AATGGATCACCCGATAACAGGTGAAGTTATCAGAGTCCAAGCAATTGAAGGCAACAATGTAATATCATGCCGGGAATGTTTATTCAGACAAA
AGGGGTTTAAAAAGATATGCCCACTTATGAGATGTTTAGATATGTCTACGGGAAAGAGTCAAGCGTATAAACAAGTAAAGTTATGAAGAAAT
TAGATTTGTCAATTCTACCGATTGATTTAAAGGTAGGTGAGGAAATGGAAATATTAACGCCCGAAGGTGATAAGGTTACAGTAAGGTGCGTT
GAGGATGAAAGGGAAAATATGTGTGATTATTGCTTCTTCGGGAAATCGGTTTCTAGAATTTGTTATAGCGTTAAATGCAGCGTAGATGAGCG
TAAAGACAGTGTAAGTTTCAAAGAAGTAAAAAGGGAGAGACTCGAGAAAGGAAAATTATTATGAAAGAAGTGATTGACTTTAAAATCGGTGA
TGAATACAAGGAGGGTGATATACTAAAGACGAGAGAGGGGATATATTTGCTCGTAGAAAAAACGGATAACAAACATTGCGTTAAATGTTGTT
CTTCGTGTTGGTTTCAAAATTCACCTTTGGACGTATGTGTACAAATAGATTGTACCGCAGGAAATTTTTATTTTAGACCGTTTGAAACATAC
AAAGAAGGTGAGGAATACAACGTAGGTGATCTATTGAAAATACCTAAACCGGGAGAGCCGGGAAAGTTCATCCTTTCAATAGTAGTAGAGGA
TGATATTGTAGACGAATTTAATAATAGTTGCGAACGATGTGTATTCAAAGACTGGGTATATCCTACGGATGATTGTTGTTCAAGAAACAGAT
GTGTAGATTGTCTTAGAGATATAAACTTAGACGATGTATATTACAAACCATTAGCGGAGGTATAAGAAATGAAGCAAAAGAAAGTGAGAGAT
TTTGAGGTGTTCAAAGTAGTACATCCGATCACAGCAAACGAAGTAACAATTCAAGCAATACCACGGGATACCATTTCATGCAACGGATGTGC
CTTCCGAAAGGGAGATTTAGAAAGCATGTGTAAAGCATATCTGTGCGTTAGTGAAAGAACGTTAGATTGTTTGGTGTTCAAGAAAGTAAAGG
AGAAATTCAAATGAAATGTTACAGAGTTTTAAAAGTTAAAGTATTAATTTAAATGTGTTGACTTATGAAAGAAGAAGTTGTTTTAATGCTCT
CTGAGCTAAGATCGCAAATTGATGATACAATTAGTCGTGTTGAGAAAGAAAGTGCAGCAGAGGACAAAGAAATCATGTCTACGTTAGACAAC
TGCGAATACATTATGTTAGGTTCTTGCATTATCTTCCCACAGCAATTTATAGACTGGTGTTTATCCCGTGGCTTTTTGTCTACCGCCACTGA
GGAAAATTCTCTTTTTGGAGGTTCTTATATGATTCAAAGATATGTGTCAAGAACAGACCTTATCCGTATGAATGGAAGCGATATAACCGTTC
ATCCTTCATTGTTGTACGCATACTTACACAGAGGTGAAAATGAATAATACAAAGGACTGGGAGGAATACCTCAAAGATGTGAGTAACTCCGA
AAAGGTGGGCGAATATATAGCCAATATGATAGATCACGATGAAAGGGAGAAACTAAAGATAC

CLONE E10
GAAAGGGAGAAACTAAAGATACTTTTAGATATTTGCGAGAAAAGCAAGGATAGCAGCTACGATCTGCCTATCGACTGCAAAATATACCTTCC
TTTAGGCTCTTTGTTGTTTGAACATGAAATGCTTGATTTTATAACGTGGGCTGATAAGATGGGATATATTCGGTGTGAAGAAGACAAGATTA
TCATTATTTCGTCTATGATTAAAAGGCGGTTTATCGTTGGATCGCTTAAAGTTATGCCGGAGATTGTGGAAGCGTTCGTTTTATACAGAAAG
CATGTAGGTTAGGGGTCTTTTAGACTCCTTTTCTTATTTATAAACATTTCGTTTTTATCCACCTTCGGAGAGTTCGAGACTAACGTTTTAAT
AATCAATATCTTTGCAAAATTGCTTTTTATTCATACTTTTGTACAAACTAATATTTGAATTATGGAGATTTATAATTATTTTCTTTCTTGCG
TGCTACTTGTTTCGTTTGTAGCGGCATTTTGTGTTAACTTTGCCCGAAAGACGGGTGTAATTGAACGGATGTCAGTGTTTGGTGATTCTTGG
TTATCTAAGGTGTTCCGGTGGTATGGTGATAGATCACTGATTAACGAGCTAATCAACTGCGATTTCTGCCTATCGTTTTGGGCGTGTGTTAT
TTGTTCAGTGATCGTGTCGATCGGAACGCTAAGCCCTATTTTCATCCTTACACCGATCTTTGCAACACCTATTTGTAGAATTTTAATTTAAT
GATTATGGAGATTAGAAATTATGTATCAATTATCCCGCCTTTCGAGATCGTGAAGGCGGTTAAGTTTAACGGTGATGTTCACGAATTAGCGC
AGCTATTGCCAAGTTTTGAACTCCTTTCCGCAATGGATGGCGTAATGATGGCACGAATAAACGGCAGCGCTTTTCGGGTGTTTGATAACGAT
TTTATCGTTCTTGGAGAAAAAATTGCTTACTCAGTTGACGAAGAAACGTTTGCCATATTATACGAGCAGGCAGATAAGGAGGTGACGAATGA
ACACGATTAAGGTAGGGAATCACACGGTAACGGTATACGAAGGCATTGATGAAATGCCTATCGTCCGTTATCAGAAGTTTAACCGTCTTATG
CTGATTGAGTCGGGAGTCGGTAGCACTATTGAGGAACTCGATACGCATTTGCAACGTGCTATTGTCTATTGCAGGACACAGCCGGAACATAC
GTATAACGAGCTAATGAATCTAAGGCAGTGTTTCAATATGGCAGCGAATGGTGTACATCCCGGAATGATGGCTTTTGCCGCCTTCGTTAAGG
CGGTCGATGGCGTGGAATATCCGGTTAACGCATCCGATTCCGATCTAAAGGCGATATTTGACAGCCTCAGCGATGCAACTATTAACGAACTT
TCTGAGCCGTTTCAGAAGGTCAAAAAAAAATAGAGGCGGAAGTATCGGTATACTTTCCACGGATGGCGGACGATCCTCTGATTAAAGAGTAT
TACGATATTAAACTATCTCTGATAAAAGCAAAGTTAGATAAGCTTGTGAACAACGTAGATAACAGTGAGGCGGTGAAGGAAATAGAAGATAA
GTTGCTAACCTTCTTCCCGCCTCGAATATTCTACGGGACTGATTCGGTAGAGATAAAGACGGATAAGGAGTTTCAAGAAATGTGCTTGGTAA
TCACTCAGAATATGCACATAAATGCACGTGAAATGTCGGTGTCTGAGTTTTACACCGCTTTCGAGATGATAAAGAGACAGGCAAAGAGAAGT
AAGAATAAATAAATTTAAATCAAATGGCGAACGAAGTAAAGGGAATAAAGTATAGCGATCTTATACAACCGGACAGCAGTATAAAGGACGCT
ATTACGCAGTTGGAGGGACTGCAAAAGATATATGACGCTATGTTAAAGCGTATCGAGGAAGGCGCAAAAGGGCTGCAAAAACCTATTTCAGA
AGGTGGAGGCGCAACGGAGGAAGGGCGCAAAAAGATAGACGCCTACGAAAAGCAAGTGCGATCCTTGGCGAACGCTGAGATACAATTGAAAT
TGGCACTGACAGAGACAGCGCAGGAAATCGCAGTATTGAAGAAACAGACAGCCGATCAAAACTATCTGAATAAGTTGCAGGCGAAGTTGGCT
AATAGTATGGCAGGAAGCTATAACGCTTTGTCGGCACAATACGAGCTAAACAAAATAAAGATGAATAATCTGTCGCAGGCTTATTTGGAGAA
TACGGAGGCAGGAAAGAAGCTTGTTAAAGAGACTGCGGAGATTTACGCAGCGATGGATAAATACCAAAAGAGCACGGGAAAGCACACGTTAA
GCGTTGGTAACTACAAACAGGCGTTCGATGGTTTAGGCTTTTCTATATCACAGGTCGCTCGTGAACTTCC

CLONE E11
CACAGGTCGCTCGTGAACTTCCATCCTTGGCGATCAGCGCAAATACCTTCTTCCTTGCTATTTCCAATAACATTCCTATGGTTATAGACGAA
ATACAGAAGTTGCGTGCGGCAAACGAGGCAGCAGCGAAAGCAGGGGAGGCACAGGTAAGTATAACCGGGAAACTGGTTAAATCTCTGTTCTC
GTTTAATACCGTGATGGTGTTGATATTGACCGCCTTTTCTATTTGGGGTAAGGATATAACCAACTGGATAGGTAGCCTATTCAAAGGTAAAA
CAACAGTAGATCAGTTGAAGCGATCTACTACCGACTTGAAAGATTCCATGTTAGAGGCTGGAAAGAGTGCCGTAAACGAGTCTGTGAGGTTG
AATATCCTGTATAAAGCGGCTACCGATTCCACACGCAGCCAAAACGAGCGTTTGAAAGCTGTTAAGGAGCTAAAGAAAGAGTATCCGGAGTA
CCTTAAAAACCTATCAGATGAAGCTATTATGACGGGAAACGCATCAAAGGAATACAAGGAACTTGCAAAACACATTCTATCGGTCGCAATGG
CACGTGCCTACGAGGAAAAGATACAAAAGAACGCAAAGGAAGTTATTGACCTCGAAGAAAAAAAGAACCAAGTATTAGAGGAAGGTCGGAAG
ACTTACCAAAAGCAACAAAAGGAGATCGAGGAACTTAAACGTTCGTCTAAGGGTATCGGTGTTGGTGCGGTGGCTTTGGAAGCGGCTTTGCA
AGGGCAAGCGTCCGCATGGAATACCGCCAAAAAGGAGGCAAAGAGCTATGACGAACAAATAGCAGTTATCAATAAGTCGAGTGAGGAACTTG
CTAAAAAGGTGGTTATCCCCGATCTTCTTGCAGGGGACAAAGGAGGTAAGACGAAGGAAAGGACAAAGAAGGACTTTGATCTACAAGCTGAG
TATGAAAATAGCCGTATAGCTCTTATTATTGATTCACGTTTGAAAGAGCAGGAAGAGCGTAAAAAGGCAACGGCAGATGAACTGAAAAAGCT
AAAGGATAGCACAACGGAGAAACAAAGAGCTACGCAGTTATATGCTGATACCGTATACAATATCGAGGCAAAATTGCGTAGAGACTTGGATA
AACTGCAAAACGACTGGCGGGTAGAGGACTTGCAAATCACGCATGACCGATTGAGTGAACGCCTAAAGGCTGTTAGACGTGGCACGGCTGAC
GAGCTATTAATACAAGTGCAGTTACTCGAAAACGAAAGAGCGCAGGACGAATTGCGTATTAAACAGTCAACCGATAGCGAACAGGTGAAGAA
TGAACGTTTGCTTATCCTGCAAAGGTCGTATCAGCTTGCATCTATCCAACTGCAAAAGGATTTCACGGAAAATCAAGACAAACGTATAATTG
ATCGGTCGGTGTTCCGACTTAATCAGCAGCAGCAGGCGGAGAGTGCCGCCTTTAATATGGTGCAACGTTCGGAGAAAGAACAGAGCCGTTTC
CGGTTGAAATTAGAGCGTGAAAAGTGGGAGCAAATATTAGAGTTAACAAGGCAGTACGGAGAGCAAATCACGGGATACAACGTAAAGACGGT
AGAGGATACCATTAAGGGAATAGACAATGCAATTAAGCGTGATACTTCCGGATGGGACAGCAATCAAGGCGTATTTGGAAATCTGTTTGATC
TTGTTTTCGGTGACGCCTTTAGCGCAAAAGATGGTAAGTCAGGCGCAGAGCGTGCAGAACAGTTTAAAGACTCCATATTAGAGGCTTCGGAG
TTCGCCATAGAAAACCTAAAGAGTGTTGCGCAGGCAAGGGTAGAGGCGGCAGAAGTTGCGGTACAGGCAGCAGAGAAAGAAGTTTCAGCCCG

ACAAAAGGTTTTGGACGCTGAGATACAAGCGAGGGCGAACGGATACGCCAACAACGTAGCAACCGCACAAAAAGAGCTTGATTTTGCACGCA
AACAGCAGGAAAAAGCACTGAGGGATAAGAAGAAGGCGCAGAAGCAGCAAGAACGCATAGATACACTTATGCAGGCAAGTTCTTTGGTAACA
GCAACCGCTAACCTATGGAAAGATTTAGGTTTGGCAGCGATCCCGGCTATTGCGTTGATGTGGGGATCATTTGCTTTTGCTAAGATAAAAGC
CTCACAGCTATCTAAAGCCTCGCAGGACACGGAGGAATACGGTGACGGTACGGTAGAAATGATTGATTACGGAGGTTCGCACGCATCCGGAA
ACGATGTAGATTTAGGTACGACTAAGGATGGTAAGCGTAGACGGGTAGAACGTGGTGAATACTTCGCAGTAGTGAACAAACGTTCATCTCAG
AAGTATAAGAAACTCGTTCCGGACTTGATTAATTCGCTAAATAAAGGTACTTTTGAACAGAAATACTTAAACGCCTATTCCGGTAGTGATGA
AGTAACGAATATAATGCAAGGTTCAACGGTTGATCTGTCTAAGGTCGAAAAAGATCTGAAATCAATCAAAGAGCAGGGGCGTGTTAAGTACA
TCACAGGTGCAGACGGCACGATAATTGAAGTAAGGGGAAATATTAAACG

CLONE E12

GTAAGGGGAAATATTAAACGAATAATTAAATCATAATGAACGTTAAAGATTTGCGGTTTAAATTGGGGGGTGTAGAAATACATCCCCACTAT
TCAGAGCTAAAACGGAAGTTTGGCAAAGAGAATCAACAGGAGTTTTTCAGAGAGTCGATAGAGGGGAGTTTAACGCTGATCGGGGCGGACTA
CCTTCTTGTTAAAAATGCGAGTATTGAGGATATTTTGTACTTGCAAATAGAGCAAAAGGATAAAGGGCAGCTATCAACGCAGTATCAAGTAA
TATTTGAGGGCTATTTCAGTAAGACAGATTGTGAGATAGACAGCGATAACCGTACGTGCAAAGTGAAGATAAGCCCACGGGACGAATACACC
GACATAATGAAGGGTATTGAGAACAAATACGATCTTATCAAGCTTGCACCCGCATTGTCTCAAATAGGGGTATCCAAGCGTCCGATTGTGCA
AGTTTACATTGCGGGTGCATCTACAATATCGAACTACCTTGCAGGCACTCACTACGAAACTGAGGTTTTCAACGTTGTAACGGATAACAAGG
AGCTAACGGATAAGAATTTCTTTGCCTTCTTCGCTGCATACAACGAAATAGAGGTAAAGGCAGTGCCTTATCAGTTCTTTAACGGAAAGTAC
TACGGAACGAATGGAACGTACACTAAATTGGATGGCAATTTCTCAATAAAATGGACTCTAAGCGAAGGTTTAAATATTGGTTTCCTTCACTT
GGAAAATAAAGAGGGAACTATATTGTACCGATCCGATAAGATCAATTGGAGCGATAAAAGCTACTACTACATAGATGTTTCTGAAATAACAT
TCACAAGAATAGTAGATGATCCGACACTTCCTCAAAAGTTTGGCGGAAACACTGTTCTTTTGCAGAAGCTATTTCAAAGAATGTTGCTTAAC
CTTCCGGAGTTGGACGGTAAACCTACCGGGAAACTATCATCAGAGGACGTTTACCCTACCAATAGCAACTACATGTATGCCGCACCATTAAA
AGGGAACTACTTTTATACGTCTACGAAGGTTCAGAACGAGCCAACAGAGTATGGTGTAAATGATGAAGGCAATTATTTTACCGATAACTTCG
TTCCGTCTGTGGCGGGTACTGGAAAGTTGTATCCGGTATGCCGTTCACGATGGGGGAATATGTCGATTTGGTTCGAGTTTGATTTATCCTAT
GCGCCATTGGAGGAAAGAGCGAGAAAGGAGTATGTTTTAAAGGACTCGTTCGCCATACAGGACGCTATTAGGGCGCTTATTAAGCAAATTGA
TCCCACTTTGACGCACGAAGCTACGGAAGAATATAGTAAGTTTTTGTATGCTGCCAATAACCCTATTTCCGGTGCACCTTTTAAGGTGTTCA
TCACACAGAAAAGCAACATCCTAAAGGGTGAGTATGACCGTCCGGCAAAGAAGGCGGAAACAACCCTCAGCGATATAATGAAGATGTTGCGT
GACACGATGAAACTATATTGGTTTATAGATGGCGATAAGTTTAGGATAGAACATATTTCTTACTTCATAAATGGCGGAAGTTATACCGGTAG
CGGGACGGTCGGCATAGACTTAACAAAGCTTAGATATGCAAAATCGGGTCAGTTAATGACGTGGAAAACTAACACGGTCAAATATGATAAAA
CCGATCTGCCTTCACGGTTTGAATTTTCTTGGATGGACGATACAACAAATACGTTTGCGGGTTTCCCTATTGATGTGAAATCAAACTACGTG
CAAGAGGGAAAGAAGGAAGAAATAAGGGTGTCTAACTTTTCGTCCGATGTAGATTATATGCTACTATCACCGGGTGACTTTTCACAGGATGG
TTTTGCGCTGCTGGGAGCTACGCAGATAGGCGGTAAATGGAAACTACCGTTTGTTACATTCAATTTGGTAGACAAGAACAATAAGAAGTACA
CCGTAAACCCCCAAAACGGCTACATGTCGTTCTTGCACCTCGTTAAATACTACATGCACGATATGCCAGCCTCAGAGATTGAACACGGAGGC
GATCAGACGATAAGAGTGAGAGGAATAAAGCGGAGTATGACGCAAGATTTATCTTTCACATACGACACCACACCAAACCCCGTGCAACTGAT
AACAACGGATATAGGCAACGGGAAACCGATAACTATGACTGAGGATCTAACAACTCGCCAAATAACCGTATCTTTATCTTACACCCCCTTAT
AATAGGGGGTGTTTTCTTTTAAATTGCTATCTTTGTGCCTATAATCAATTTTTTAATCAAAATGGAAGTACATAACAACTTTAGTCCTTTGG
CGTTTAGAAAGAAAGAATCTAAAGCCACATACGAAAAATGGTACGCTTTCGGGAAGAACTACGCTATTCCTGCAAGCGCAAACACGCTAACT
CCTTTCCAGTTTACAGAGTTGAACATACCAGTCTTTGATCCCG

CLONE E13

CATACCAGTCTTTGATCCCGACACGATCGAAGTAGAAGCGGTTAACGAGGAAACGGGAGAGTCGACAAAAACGGGTGTATATGTTAGCTTCG
ATGTAATGCCCGAACATGGCGGTGTATTGTACGTGTCACCCGGCAAGAACTCGTTTAGGGAGGCTTTGCCACAGGGAACGTATAGAGCACGT
TTTTCAATCGGTGATGAAGTATATATTTCGACTCCTTTTTGCGTTATACCCGGCATAGAAACGAGTAGCAAATATCTATTGATTGAATATTG
GAACGATGAAAAGATTGCCTATCCGGGTGGATTTATTACAACGGGTGCGAACAATGACTTCCGGTATCAGATGTATGTTCCTGCAACGATCT
GCAAACCTAAATACGAGTTTGAAGAAGAGCTAACCAAACGTGCCGGATACAAGTTTTTGGAACTGCAAACGTCTACGAAGGTGTACGCCTTT
ACATTCGTTGCACCGGAGTTTATTTGTGACGCTATGCGACTGATTCGCCTATCTGACTATATCCGAATTTCGCACGATGGCGAATATTACAA
CGCTCTCAACTTCGAGTTTGATGTTGATTGGCAGGAACAATTATATTTGGCTGCTGTTGACTGCCAGTTTGAGACGGACTCAATCATACAAA
AACTCCCTTCTTTCAATAGACGAGATAAAGCGTCTTTTTATAATGCCCTATTAGCGAACATTGATACACCTATAATGTTCTCTCCCGATACC
GTAGGGCTGTATTACAAAGAGTATCGGGAAACAGAGCCAGTAGTCAAGGGTAAATTGATACGGGAGTTATCCCCTATTGACTTGATAGATGA
AAATACAACTATTGCCGTTGATTTGGGTACAGGTGAGGCGAGAAAGTTTAACTTATATCGAATGTTGCAGGACTACATTTCTAAAACCCATG
AAGATGCAACAGACTTTTTGTTACACCTTCGTGGAGGCGCAACGTTCGGTGAGGGCATAACTGGTTCTGCCGCTTCTATCAACGCAGTAGGA
GATGCGGAGGTTCAAGGGCTAAACGCACGTGTAACCAAAGTTAAATCGATTGATTCGGAAGATTATGTAACTGTTAATAAAACAGCCTTCAC
CGTAAACAAACAAGGTGATACGGCTTTAAATGCGCTTAATGCGAGGGGAGATTCCCACTTGCAGCAAGATGTGTATACCGGAAACAATACCG
GAAAGATCACCAAAGAAGGACAATTGCAGTACCTATCAGCCGTTATACAGGAGTTTATCACATCACCCACCTTTGTTCCCGGTTTTTTGGGT
GAGGGCTTTAAAATATGGGTTGAGAATGGCAATTGGCATATAGAATGTGACAATTTGACAGTAAGACAGACTATGAATATATTTGAACTACT
TATCCAAAAGATAAGGAGCGTTAACGGTGCATTGGTCGTGTCCCAATCAAACGGCAAATTGTCAGCTGTTGAAGAAGTAGGAACGCAATATA
AGCTAACCACAGGAGAGGAATTTCCCACTTTTCAAGAGGGCGATTTAGTCAGGTGTCAGACGTTTGCAGGCTATCAAGGTGCGGGACTTACC
TTTAACTTTACTCAGTTTGCAAAATATGACTATTCCGGTGGTGCTTTTGATAGCAGCTTGATTGATGTTACACCCGACTCTATTAGCTTTAA
CTTGAATGATACTGGTAATTCCGGTTTTGCATTCTATAAATTTTCAGAGTCAAGCCCTACACCAATTGAAATACCTTCATTTACCTTGACTT
TGGAGGGAGGCTATCCTGGTATGATGGCTTTTGCTGCCGGACTTGATTCAAATGACAGTCCGGTAGAGGGTGTAGGCGTGTTGCTGCAAAAC
GGTGATAATGTTATTCCGGCTATTAAAGCAGCGCAAGGCATACACAACTTTGCTATAACAATAACTGGTGATTCCGGTCACGGTGATGGTAA
GGTTACAGTAAAGCAAAAGAAAGCGGCAGGAAGCGCACCAAACAATAGCTTAGTTAAATTCTATTGGGTGGAAGTTAAAGCGGTTGACGGGA
CTTCTTTCTTTGCAGATAAAGCGGAGTTCAACGGTGTTGTTCCGGCTGTCGGTGATGAAGTCGTTCAGATGGGAAATACGAAGAACCCCGAA
CGGCAGGCGTTAATTTATATCACAGCGCAGGAAAGCGGACACCCGTACATAGAGATATTGAACGGAGTTAAAACTAAATCGTTGTCCGGTAC
GAATAGAACACGTCTTGGCGATTTAAGCAACATACAAGATTCTGCATTTCCGGAAGGACAACAGCCATCCGGTAGCGGCTTGTATTGCGATA
ACGCTTTCCTTCGTGGTATATTCTTGTTGAGAAACGGAAAGTCAGTTGAGGATGAAGTAAACCAAGCGAAGCAAGATGCAGCCAACGCAGCA
ACAGATGCGGAGAGAGCACAACAGACAGCGCAGGAGGCGAAAGATAGGCTTAATAAATGGGCTGACGATGGTTTTATATC

CLONE E14

GGGCTGACGATGGTTTTATATCTCCTACTGAAAAGCCTGCTTTGATTGATGAAGGAAAGCGCATACAGGCAGAGTTTTTGCAGATAAAAAAT
AACGCTGACAAATACGGTGTATCCGTTACTGAATATACCAAGGCTTATGAAGATTATTTAAATGAACTTAGATACCATTCCGCCCAACAGCC
GGAAGATATTGCGGTGCGTCCGGAACTTGCAAAGACGCAAACGATATACTACGATCGGAGAAACGGAGCGTTGAATGCTATTGCGAACGCTG
CAAAGAGCTACGTAGATGAAGCTGACAAAAAGCTAAAGGAGTATTTAGATACGGAGATCACAGCAATACCGGGTAAGATTGAACTCGCTGTG
CGTAGTTTGAAAGTGGCTGATGTTAACTTATTGAAAGGTGCATATAAGGAGCTGAAAAATGCTGACTATATCGTGGGGTATTACTTTTATGA
TGTACCAGTTATAGACGGAAAGGAGTACACTTTAACCGTATGTTACACACTTGGAAGCAGCAATACAGAAATGGCAGTATACTCTAATGGTG
GCACAAATCGTATTGCTACCCTTGTGACAAAAGGAGATAAAGTTGTAGAAAGTTATAAGATTACTATGGCAGGTTATAAACCGACTTCAGCT
ATGTATTTCTTTCAATTCCCAAACGGTACATACGGCTCAAAAGTACATTGGGCTGTTTTGACTGATGGCAATTTAGGTGTAACCAGTTGGAT
ACCGTCTGCAAGCGAGAAAAATGTGGGGTTAAAGAATCTTTGTTCTTTTAAGCGTATTACTGATGCAGGGTTTACCTATGCTAGAAACTACG
AAGATGATGGAAGTTGGTATATTAATCCTGAACTTTTGCATAGTGAAACCAGATTAGCTAATAAGGATATGTTTGGTCTAACTTATGACCCG
AATAAGCAATATTATCTATTCATAGACGCTGCTAAAAGGACTGATACAGAAACGGACAAAAGATGTACTTATTTATAATTAAATATACTGA

TGGCTCAGAGGATATAGTTTGTGAGGTTTATTCAGATAAGGTAACACATAATTTCATCACTACACAAAAGCCTATTTCTAAAATAGTAGGTA
GTTATAGTTATGGTTATGGTACAACTATTAGAGTCGGTTTATACGAAACGAACTTCCCCGTTTCTTGGAGTCCAGCGCCGGAAGATCAGTTG
TATCAATCTGTTAAGTACACCGATACGCAGATACTTGCCGTTGACGGAAAAATAGAACTATCTGTAAAGACTAAGGTAGAAAATTTGGGAAT
AGGAGCTAACAATTTGTATAGCTACACGAGTTCACCGCTTAATCAAATGAGTTCTGAGTCAATGACTATAACAAGGCTTATAAGTGAACACG
GATTTCATTTGGTGGGTGCAAAAGGCGGTCAGTCCTTTGTAAGAATACCTAATGTTATACCGCCTATGCCCGGAAAATACACTATTTCCGGA
TGGATTAAAGGTAGCCAAAATACCCCAGTTGGTTTTAGATTGGATATTTGTGACTCTGAATCGTATATTGTTAGGTCTAATGCACAAAATAC
ATGGAGTTATTTTAAGCACACTTTCGATGTTACGAACAATACAGAAGAGCAAAGCGGTATATATAATTTTGTTGATATACAAGATATATCAT
GGGCCAATATATGGATTAAAGACTTTAAAGTCGAATACGGTGAAATAGCAACCGCATGGAGTCCAAACGAGGCTGATTCGGTGTATTTTTCA
AAAGAATATACAACGTCACAAATAAACATTGTTGAAGGTAAGATAACATCCACCGTTGAAAAGATTAATGCGGTTGACGGGAAAGTTACCGG
ACTTGCTTCACGTGTAGACCAAACCGAAAAGAGTATAACGTCAGTTGTTGGGGATATTAGTGTTATTAATAGTACCACCAATAGGCATATAT
CAAAGCTAATAGATTTAAGAGGATGGGACAATAATAAGTTTTTCCCGTTGGTTATAAATATTCCGGTTTACCACAAAACAAGGGTTGAAATA
AGTAGACCTCTTGATGCGGGATACGGAAAACCTTCATACGGTACACATGATGGCGGTTTTTCTATGAACTTAACGTTTGAGATGTCCGGTTC
GGGTTGGGGTTCGTTGCCAGCAGTAACTAATATCTTTGACTATACTAAAGCATGGACTTCTGCGGGTGCAAAGATAGTTGTTGATTTGGGAC
AAATAACTGAGACTTCTACTTGTGTGATGGGTATCCGTGGGGGGTCTAAATATGATGTAACCGTTTACGACACAACAGACCCCAATGTGATA
AACGTTTATCAAACAGATTATCACGGTTCGTACGGTACATCTTTCCCCGTTCGCACCGATGGTACAGAGCCATTCCGGACGTATGGTTACTA
CTCTGAGATAAAACAGACACAAGAAAGTCTCTCAGCAACGGTTGCAAAGGTAGACGATC

CLONE E15

CGGTTGCAAAGGTAGACGATCAAGGCAGGCGGTTAAGTGCGGCTGAGTTAACATTATCTGCCGATCACGCAAAATTATCGGTGGTAGAAACG
ACTGCAAACAATGCCAATTCACTTGCCGGAACTGCCAACAACAAAGCGGAAGCGGCAGGCGGTCGAGTTACCGCCACACAGAACGGTTTGGT
CGAGACAGGAATAAACATCACATCCCGCAAAATCGTGCTAAAGTCTGATAACGTCGTTTTCCAAAACAACGCAGGACAGCAGACATCCGCTA
TCAATGCGAACGGAAAACTTACTGCAAACACAATCGAGGTTGGAGAAGTTGTTGCCGGAGGTTTTGCGGCTCAGAGAATCACTACCGGGAAC
TTGACTGTGACGGATGGGGCGGTGATTGCAGGAATGACTATTTCGGGAGGTGTTCTTACTGGGAAGAATATACATATCACAGACGGGGCGAA
GATCGGTAACTTTACGATCGAAAGCGGTATTCTTTCTGCTAATGGTATGGTAGCAGGAATGCAAATGCGCTTATCTAATAACCTTATGGAGA
TAAGTAGCTCAGGACTTCGCATAGATCACAATTCCGGTGGTTACGCTATCAATGTTACAGGCAACGGACGTTGCTATATGCAAGGTGTAGAG
TCCTACCGTAAATTCGGTGTATATGAGTTTGCTGGTGCGACACAATGGAAAGCCCCCGGAGTACATTTTGCGTGTACTATTTCCTCAGCGGC
AGCAGTACGCCAAAAGTGGGGAAACCCCTCTATCGGTGTGAGTGCCGTAAAACACTCTACCGGAAGATATACAGTTCGGTTTACCGGAGTTC
ATTCACAGGGCTTTATTCCTATGGTGATGGCTCTAAATGCTACCAAGTGGGTAAACGCTTGTGTAGAGGATATTGTTAGTACGACACAGTTC
ACCGTCAAACTTATGGACGTTAATACTGGAATGGTAGATAGCGACTTTGCTATATACATTTGCGGTTTTGTTTAATAGTTAATATTTGCGGT
AAGTTGGTTTATACCTTCTTACCGCTTACCTTTGTACCAAACATTAATCAATTAATATAAAATTATGGAAAAGAAAAGTTTAGATTTTGATT
TAAAGTCAGTAGTTTACACGAAAGAAACAAAAGTGATGGACTACCATTTCGAGACGGAAAACGGCAAGTACGTAGGTCAATTAACAACGGTA
TCGACAGAGCCGGACAAGTACAACATTACCCACTGTACGGCTGATGTGTCAGAGAAACAAATGGTAGAAATGCCTGGAACTTCCGGTAGTCC
AATTCTGCAAGAACAATACGTTCCGGTCGGATCGCTTGCCATCCGTGACGGTCGCTTTGAGGCAAACCAGTTTCCTCTATCTACTAAAACAT
CCGTCTATGTGAACGACTTTCAAAATTTCATCTTTGCGTTAACCGCACCTAAAACAGTAGAATAATGAATGTCACACAAGAACAGTTAAGGT
TAATGCTTGTATCGGTGATAAGTCCGATACTTGCGTTTCTTACCCCTACGAGCGGTTTTATAACCGCACTTGTGTTCATGTTCGGCTTTAAC
ATTGTTTGCGGTCTGCGTGCCGATGGGGTTAATTTGTCGGCAAATGGTGTTCGTAGGTTCACTATGCTGAAATTCATCTCAGCCGTGCAGGA
ACTTATTTTGTACATCCTTGTGATAACCGTTATCTTTTCGTCTGTGGCTAAGATGGGGGATCACGACGCAGCCGTTCTATCGGCAAAGACGA
TTACATACGTCTTTATGTACGTATATCTGTCGAACGGTTTTAAGAACCTTTGTATCAGCTACCCGGATAACAAATCTTTCCGACTGATATAC
TACATTGTCCGGTTCGAGTTTAAGAGGCTGATGGGAGAACGTGCCGCAAAGATAGTCGAGGAACACGAAGAAAAGATTGAGATTGAAACTAA
GTAATTAACACGGGAGGTTTAACGCCTCCCTTTAAACTTTATCAAAATGAAGTATTTCACATTAAAAGAGCTAACACGCTCAACAACAGCAA
CGGCAAAAGGCATTGATAATACGCCAACACCGGAAGTTGAAAAGAATTTGACCTTATTAGTAGAAAACGTATTAGACCCTCTACGGGAGATT
TACGGCAAACCGATCACGGTTAATTCGGGTTATCGGTGTCCTGAGTTAAATAAAGCCGTCGGAGGTTCTGCAACGTCTGACCATTGTTTTGA
TGAAAAAACAGAAATTTTAACTACAAAGGGGTGGAGATCATATAATAATATATCCAAAGATGATATATTATATACATACAATGTAGATAATG
ATATAATAGAGAAAAAAACTATAAATAGGATCGTTATAAGAGAATATGACGGGGATATGATCGGATTTAAAAATAGCAGCGTTGACCTACTT
GTTACGGACGAACATAGAATGCTTATAAGTTACCAAAGGCATAAGTATAAAAGAAAGGG

CLONE E16

GGCATAAGTATAAAAGAAAGGGTAGTAGAAAAATATCCGAAAAGGGGGCTTTGTATTTCGATAGTTTGAAAACTAATAATGACAAATTTCAT
TTTGAGCTCGCTAAAGATGTATTAGGTAAAAGACGAATTTTCAAATGTTCTTCTTATAAAAATGGGGTGAGTAGTGATATAAGAGTTATGAA
AATATGCCTTGCATTTATTTGCGATGGTTTTTATAACAAGAAAGCAATAGGTTTTAGATTCAAAAAAGAAAGAAAGATAAAAGAACTCGAAT
ATATATTGAATAATGTTGGGTGGGAATATACGAAGCGAGTAGATAAATGCGGAGTTACTAATTTCTATCTAAGAAAAAAATACTATGATATT
ATTTTAAATATCGTAGGGAAGGATAAGAATATACCTATTGACGTATTGGAATACAATAGCGAACTACTAAAAGAACTTTTATTTTACTATTG
TTCGTATGACGGTCGCTTCGACAAAAGAAATAATAATACGGGTTTCGCAATCGGTACAACCAATAAACATAATGTTGATGTGCTTCAATCCA
TGGCTTGTATGTGCGGAATAAGATCAAATATCTTATATTACAAAGAAAGAGAGTATAACATAAAGGGGCAAACAGGTATAGCTAAACCTTAT
TATATTCTTTCGGGTTCTTTGAATACTCAATCAGATTTACAATCATGTGGATCTTTTGTAGAGAAATACAATGGAGTCGTATGGTGTGTTAG
CAATGATAATGAAACGGTTATCGTGCGAAGAAATGGTAAGGTTAGCATACAAGGTAACTGTAAAGGTTTTGCGGCTGATATTACAGGAGGGA
GCAAGGAAGAGAATGAACGGTTGTTTAATATCATTAAACACAACTTTCATTTCTCCCAGTTGATTGACGAGAAAAATTTCAGTTGGGTTCAT
GTGTCGTACAACCCGAATAACTTAAAAAACCAAATACTAAAACTATGAAACGACAATTATTTGCGTTTTTAGCGACTTTTGTGCTTTGCCTT
GGCATTGTGTCGCTATTACTGATAAACGCTGATTTACGGAAGAAAAAGTCTATTGCAGAAAGAAATGTTAGCGTCCTCACAACTCAGAACGT
TGCGTATCGGACGAAAAGCGGTCAAAGTGCCATGAAAGTAGAGGAATTGAATCTGACTTTAAAGCAGTACCGGAACACCATACAAGGGAAGG
ATAACACTATAAAGGAGCTAAAGCAGTCTATTAAGGACTTGAAAAGTCACACAAGCGTTCAAACATCAACTGAGACGCATTTTAGCACGCCA
GTACGGGATAGTGTTGTTCTTCGTGATAGTTTGGTTATCGACACAATGAAATGCGTAAATATGCGCTCTAAATGGCTTGATATATCCGGCTG
CATAGATAGCAACGGCACGTTTGCCGGAACAACCGTTTCCCGTGACAGCTTAGAGATACTTAATATAGAGCATAGAAAGCGGTTTTTGTGGT
TTCGACTAAAGAAGGTGAAGTATAGGGAGTTTATCGTAACGAGCAAAAACCCCTACACTGATATAACAGGTTTTAACGTAACTACGATAATA
AAGTGATAATTCCATGTTAAAACAGTTAATGCACGTTAAAGTATTTGCCACTGAGAAATATATCCGTATATTTGCAGCGTAGAAGTTATTAC
TAACGTCACTAACAGCGGTTATTGATTTTCATAAAGTTATGTTTGTTTAATTATTAATATTCACATTCTCAAACTGTCGGTATGCGAATATA
GACAGTTTTTAATTCGAACATTTTCACTAACTATATATATTGGGTTTTGTCATAATTACATTTTTCCCCCTCCGCTTGTGAAAGTAGAGGGG
TTTTTTATTGCCTTATCCGAACACGCCTAAAAAGTTAAATTCGTGTTAAACATTAAACTTATGCTTTGATATTTAAAATATCTCCTTAACTT
TGCAACATCAAAAGGAAACGAATTACTAACAATAAAACTTAGAGTTATGGAAGAAAAGGAATTTATTTATTGCTTGACCGGAGAGATTAACG
TATTAGGCACTGTCAAGGCTAAAACAATAAAAAGTGCTATGAAACTTGTAGCGGCTATTCAGAGAGGTGCTATATTGAATGATCCGGAAAGG
AAATCAATCTTTTGGAGCGTTTCACGTGCTGATCTCCCGTTTAAACTTGGTCGTATTGTATACACAATATGCTATCCCGATGGGTCTATTCG
TTCACATGTATGCTAACAATAAAAATTTAGAGTTATGGAATTAGTAAAATTCAGAGAGGCAAAGAGGATAATGGAAGAAAAAGCTTTTTTGC
AAAAAACGCTTGAAAGGTTTCAGTCCGGCTATCTTAGTAGAACAGATTTATATTTCAGTTCGGGAAGTAGCGTAACATTTTCAGAAGGGGAT
GGCGAATTTTATGAAGGTTTGTGCAAAGACTTAGAAAA

CLONE E17

GTCTATAAGAGAATCTATTGAAAAGCGTATTGGCTATCTTGAATCTAAATTTGATAAACTATGATACGATCATTTAGTAAGTCGGGTTCAAC
ATCTATGCTGACAGATAAGGAAAAAGCGTTTAACCGCTACTGCCTAACTAACAAGGAAGTTTCATATAACTTAATGCGTATAGAAATGGCAG
TTGTTCAAATGTCGCATTACGGCAACCGTTCATCAGATGTCACGCTAACAACCGATAGTTCTGAGGTTTTGGATGCAATTTATACAGTCCTA

ACAAATGAAGGGTTTAAATACTCTTTCAATTTACCTAATAAAGTATTAACCATAAGTATTTTTTAATTTAAAATTTAATCAAAATGAAAGAA
GAAGTAAAATTGTTCAGAGCGTTAATCATTGTTTTTGTGTTACTTGTGTTCACCTTCGTGTTAACTTCGTGCGGTGATGATAGTGACAATGT
GTATCAAACAGAATATTCTATTGATGTTCCGGAATGGCAGACGGTTTATGTTAATGGTGAGGTTACAACGTCTATGTCTCCATATGTTTGGG
AACATGTGGACTTATCAGACAAATGTGTTAGAGTATTCTCAGCAGGGCATGTTAGTTATCACAAGGTTACAAAGGTGTCACACGATGATTTA
GGCTTTACCGTTTATTCAATAGAAAGTAGCAATAGCGAAAGGTTTGCATACAATAAGAATAAAGGTATATTGCAATATTGGTGCACAAGAAA
TGGCATTGAAACCGTTGTTGTTTATCGTGAATTAAAGTAAGTTTCATTTTACCCTCACCCGGTGGAGGTTAACCGGGTTATTAAGTATGAAA
GTAATTGTTAGATTTAAAGACGAAGAAGAAATTACCAATTTTGAAGCTAAATACGGTTTAGAGGTAGAGAACCATAACGGACAAAGATATAT
CATTGAGTTCGATAATATGGGAAATTTGGTGGTTAATAGTCCGAACGGTGCGTTATTGGTAAAACCACAATGCAGTAATAAAATATCAATTA
GACCCGAATAACATGAAAGAGATAAACGAAACTCAATTACAGCTATCGACTGAGGGAAAAAGACTTCCCGATATGATAAAGCAGGCGAACGG
CATACACGAACTTGTTAAGCAGAAACTTTCTGAGTATAACTCAATAGAGTATACCGATGATAATATAAAGGTGGCAAAAGCCGATAGAGCCA
CTTTAAACAAGGCGAAAAAGGGACTTAACGACAGCCGTATAGAACTTGAAAAGGCTTGGATGAAACCGTTCAACGAACTAAAGGATGTTGTT
AACGAAACTTGTAAGCTGATCGGTGAGGCTTCTTCGCGCATAGATAGCAAGATAAAGGAAACGGAGGAAAAGGAGAAGCAAAAGAAACTGGA
TCAAATAAGGGAGTATTTCGAGGAACACAATGAAAATCTTATCTTGTTTGATTTTGCTTTCCGTCCGGATTGGCTTAATAAGACCAAAGCAC
TTTCAGTTGTGAAAATGGAGATAGACGAATTATTTAAAACGGTTGACGATGATCTTAACAGACTGAAAGAGCATTTTGCCGGAGAGGCGTTT
TATATTCCGGTTATCGACAAATATACGTCTACACTCGATTATAACAAGTCGTTCGATTATGGAAATCACCTAAAAGAAGCTGCAATACAAGC
CGCAAACAGACAGTTTGAACAGAGGGTGACAGATAACACGCCTCAGCAACAAAAGCCCGAAATTAAGCTCCAAAACGAGCCAAAGACGAACG
AAGAAGAAGTTTATATACGAGGTTTTAAAGTCCATGTAACGAGAAAGCAGGCTTTTGCGCTTGCTGAGTTTATGAATAGCCACAATATAAAG
TTTGAAAGTATATCAATATAGACGGTAGCCCAATTGGGCTACCTTTTTTGTTTTGTTTGCAATTGTTGATCTATTGTTGAAACCTAAGGTTT
CACTTAAACTTTCAAATAATGTGCTTATATTTGCAGTGTCGAAAGAAACAAAGTAGTAACAATTAAAAATTAGAATTATGAATGAAATTAAA
TGCGTTAAATGCGGATGTGTGATTAATACAGATGCAGGATACTATGCTGCTTTTGATGGTGGCTTTTGCAGAAAGTGTTGGATTAAACAATC
AGACAAATTTAAGAGTGAACAGTTGGTGAAAGCACTCGAAAAGAGATTCAAAGGTAATATTAACAATAATTTATTTTAAAATGGAACAGTAT
TTAGACTTACTAAAAGAGACTTTAAATTATGGTGAAAAGAGATCAGATCGAACAGGAACGGGAACTATTAGCTTATTCGGTTTACAACGATC
TTATGATTTGCGTGACGGTTTCCCGCTTGTCACAACTAAGAAGGTATTCACGAAGGGAATTATATATGAGCTTCTTTGGATGTTAAAAGGAG
ACACCAATATAAAATACCTAAATGAAAGTGGTGTTCATATTTGGGACGAATGGGCAAAGCC

CLONE E18

ATTTGGGACGAATGGGCAAAGCCTTCCGGTGATCTTGGACGTATATACGGTAAACAATGGCGTGACTGGCGTATAAATAGCAAGTTAAGAGT
AGATCAAATTGATTCAGTTATAGATATGATTAAGTTCAACCCGAAGTCAAGAAGGTTAATTGTTAGTGCTTGGAATGTTGGAGAAATACACA
TGATGGCACTTCCTCCGTGTCACTGCTTTTTTCAGTTCTATGTGTCTGAGTCCGGTTATTTGGATTTGAAACTGTATCAAAGAAGTGCAGAC
CTATTTTTAGGCGTTCCTTTCAACATTGCGTCTTATTCTATCTTGCTGTCTATGGTAGCGCAGGTTTGCGGCTTAAAGCCTCGTAGATTCAT
TCACACTATCGGGGACGGACATATATATTTGAATCACGTTGAACAGGTGAAAGAACAATTGAGTAGAGAGCCGTTCGCCCTTCCCAAATTGG
AATTAAACCCGAATGTTCGTAATATATTCGATTTTAAGTATGAAGATATTAAGATAGTAAATTATAACTGCCATCCGGCTATAAAGGGAGAG
GTTGCGGTATGAATGAAAAAGAATTTTACAGGTTTTTAGCCTATAATAAATTGGTATATTTTGAAAGATACCTTCACATGGAATCTGTATAT
TATCTGAATAACTTGCTAAAGAAAACCGTTAATTCGTATTTGAGAGATTGTATATTGAACGCTATAAATCATAAATTAGCGGGATTATAATT
TAAAAGGGATGTGCAACGCTTTGCCATCCCTTTTTAGTTTCTATATATCACATACCGAAACTATCGTTGCTCTATGAAACAAATCTAACAAT
ATGTAGTAACAAGTATGAAAGTGATGCAAAGGTAGGCTTTTGTCAGTTTCTAATGATCAAAACGGATCGTTTTACATTTCATTAACAATAAA
ATTAAAGAATTTCTTTGCGTATTTAAAGTTTATCCTTAACTTTGCAACATCAAAAAAGAAGTAGTAACATTAAAAACGAATAATATGCAGAT
TAAAAAAGATCAAATTTACAAATTGCTTGTGCAGGTTTGCAAGAATGAAGGCATTCCATTCTCCTACGAAAAACTTGTTTTGTTTTTGAATA
AGTACATTGATGAAGATGAAGAAGATTCAGTATTTGGATATACAATATCTGATATTGATATTTCAATCGCTAAACATATATCGGTTGATATT
TGCGGAATGCTTACATTAAGCAATATTATCAGTCAATTAACTTGTATCGGTGGGGGAGATTGCCCGAATTGCGGAGGTTTACTCAGATTGAT
AGAATCTTATCCCAAATTTAGCAAACAGTATTGCGATCGTGATTGTGAGCCGGAGAGAGAGGAAGAAAATGTATATGAATGTTTAACATGTG
GAAAGGAGGTTGTTTTATGAATATTGAAAACACAATGATCCGTATCAATGATGCGATTATAAGCGCACGTATGAACGGCAAAAAGATTACGA
AAAAGGATATTGCGGCTTTGCTGTGGAAGGACTCAAAGCAAAGAACGCAGGCGGTAAACATGTCTGCCTTGTGTAATCACAAAACCCAAACT
ATAAAAATAGAGTGGGTGAAAGAGATATGCGAGGCTACCGGAGTCGATGCGAATTTCTTGTTTAATATTAACCCTAAAAAATAAAAGTTATG
ATTAAAAATTTGCCTAACATTCAAAACGAAATGAATGTTCAAAAGTCGAGATATAACAAGTTTGGCGGATACAATTACCGTTCGTGTGAGGA
TATTTTGCAAGAAGCGAAAAGGGTGTGCGAAAAATACGGATGTTATGTTATGGTGACTGACTCTATCGAATTTATAGAAGGGCGTTTTTACG
TGAAGGCAACCGCAAAGATTGTTGAAACTGAAACCGGGTCTATTGAAACGTGTTCGGCTTTTGCACGTGAAGAAGACAGCAAAAAGGGGATG
GACTCGGCACAGCTAACCGGGGCGACATCCAGTTATGCACGAAAATACGCCTTATGTGGACTTTTTGCGATAGATGATAGCATAGATACTGA
TTCTACGAACGGAGAGCCGGAAGCGAAAGAAAAACGGCAAAAGACAGCCTCAAAACAAGCTATCAACCAAAGTAATACTGGAAGCAACTCAA
ATTATTTGGGTGTGCTTCTTGAAGAAATAAAAAAAGCAACAACTTATAAACATTTGGGCGATATTCACAAGAATAACGCTAATTACCATCAA
AATAGTGAGTTCATGAACGCTTTAGTTGTCCGTAAGGCGGAACTTGAAAAGGCGGAAGCGGAAGCAAAGAAAGTATAAATAATTCGGGGGAT
GCGTTCCCCCAATAAAAATAAAAGCAATATGAAAGAACTAACATTACTCCCCAAATTGGTTAATGCTGATGTAACGTATATCAGCGAAACAC
ATGAATATTTTTCAAGCGATTTTAGAAAGCTGAGAGGGATAACGGGTTTTATCAATG

CLONE E19

GGGATAACGGGTTTTATCAATGATCAATTATTTCCCGGCAAACTTGACAATATACCGGATAATATTTTGAGATCGGCAACTGAGAGAGGGAA
AGCGGTTCATGATGAAGTTGAGAGGATCGACAAAGAAGGTATTGAACCGGAAACGGTCTACGGAGAGAACTATTTGAATTTAAAAGCCGGAA
GCGGTTTAATTCATATCGCATCTGAGTATATTCTAACTGATAACGAGTTTATCGCCTCACCGACCGATAAAGTATATTTGGGTAGCTCTGAT
AAATCAGTAGTTTTAGGTGATGTTAAAACTACCTATAAACTTGATTTGCTTTATCTGTCTTGGCAGCTATCAATATACGCTTACCTTTTCGA
GAGACAAAACCCAAATTTGAAAGTAGAGGGACTTATCGCAATTTGGCTAAGAGGTGACAAGGATAAGGACGGAATTTTCTCTGTTGAACGCA
TACCGGACAGCGAAATAGAATTATTCCTTAATTGCTGTAAGAATGGAGTTCGATATGTAGACAATGCAAGCAAAGATAGCTACGTAGCAAAA
TTGGAATCATTACCCGCAAAAGTAGCACATATTGAAGAAGGCGTTTACGAACTTCTTGAAATGCAAAAGAAGATAGACGAACATTTAGGCAA
GTTCAAAGAACAGTTGTTAGGTCTGATGTCTGAGGCGAAAGCTGATAATATAAAAGGGGAACTTATTTCAGTCACAAGGAAGAAAGCGTATA
GCCGTGAATCACTTGATTCTAAAGCACTGAAAGAACAATATCCGGAAATATACGATCAGTTCGTTAAAACATCAAATGTTAAAGAGTCAATT
CAATTAAAAGCGTTATAATTATGGTTATAAATGAAGATTTAGCAAAAGAAATCGGTTTAGATGATTCAGTAGTATATTCAGTTATGTTTCTT
ATACTTTGTACTGATACATATAAGGATAAGTTTAAGGGATGTAGGGTAAAGAAAGAACCTAATACCGTTTTTATTACGATTTCTAAACTTAG
AGAAATTATTCCTTTTATGTCTAAGAGCAAATTATATAATTCTGTAAACAGATTATTAAAGCGTGGGTATATAAAAGAGGCTAATTATAGAC
TACCCGGCACTAATACAACTAAGTGTTATACAATCGGAGAACTAAAAACAGCAAATAAATGAAACAATTTCATGAAGAAAACCCACCGATTG
AGGAAGATTTTAACCCGTGGGATGAAAAAGAAAGATCAGTATAATTTTAATAAAACTAACGAACAGGCAAAGGATAATTATGTACTTGAAAA
CGATTTAGATTATGAAGAATAGACTTATGAAAAGGATTGTTGGTTATTCGCATTATGTGGATAAACTTGTATATGCAACGGTAGACGGGGGA
AGAAGGAAAATAATGAAATTAAAGCCAAGGAAAAAGAAGTTTTATACTTCCCTCGAATATTACAGGGCTATACAAAAGATGTTTGAAGGATG
ATAACAATATCTGAAAGTTTTGCGAACGAGATCGGTTTAAATACAACCCGATGTTACCAACTTGTTAATGTGTCACAACATTTCGAGTAACC
TATAATACACCCACCAACGTTTTTTAGTTGTGTGGGTGTTTTTTGTACAGCACTATTCTGTGCAGTCTGTCTATACCTAATTATCAGATATT
TGCAAAACAAAGAATGTTAAATCATAAAAAATAGATGTTCTTCCATTGCAGTATATAATAATAAACGCTATATTTGCAGAGTAAAATTTAAA
ACAGTATGTATATGAAAGTAGAAAACTTAGTTAAGATTAAGAGTTATGCCGATTTAAAGGGAGTTACAGTACCTTGGATATGGAGGCTTATT
AAGAGGGGAAAATTAGAGTATATTCAAATTGATGGTGCATGCTTCATTGAGTTAACGGATGAAGAACTGAAAAAGTATGCGGAGTACAAAGA
ACGGATAAGTTCATTTTTGAATAGCAAATAGTATTAATCATTAAAATTTTAGAAAATGAAAGAATTAGTTTTTAAAGGAGAATCAAATCAAG
TTTTAACTAACAGCTTATTGGTAGCTGAAAAGTTCGGGAAAGAGCACCAACATGTATTGCGTGATGTGAGAAATTTAATAAATGGGGGTGTG

TCCAAAATTGGTGATACCCCCGCATTTGCGGAAGCAACTTACATTCATCCGCAAAACGGACAAGAATACCCGATGTTTATAATGAATAGAGA<br>CGGTTTTACATTGTTGGCGATGGGCTTTACCGGAGAAAAGGCGCTTCAATTTAAATTGGAGTACATTAACGCCTTTAATAAAATGGAGGAAA<br>CTATTAAGAACGGAGGTTTTAACGTGCCTAAATCGTTCCGTGAAGCATTATTGCTTGCAGCCGAACAGCAAGAAGTTATAGAGAATCAGCAA<br>AAGCAGATCGAAGAAAAAAAATGCAAAAATCGAAGCTGATAAGCCGAAAGTTCTGTTCAGTGAAGCAGTC
CLONE E20<br>GTTCTGTTCAGTGAAGCAGTCTCCGCCTCGAACAAATCTATCTTAGTGCGTGAACTTGCAAAACTTATCACACAAAACGGTTATCAGATCGG<br>GGAAAAGCAGTTATACGAGCGATTGAGAAAAGCCGGATACCTTTGCAGCGTTGGAGAATCACGCAATCAACCTACACAGACATACATGAATA<br>TCGGTTTGTTTGAGATTAAGAAGCGTGTTATTATGGACGGTGATGAAGCAAAGGTTTACAATACAACTGTCGTTACGCCAAAAGGAGTACAT<br>TATTTCATTAATAAGTTTTTAGGGAAGGGAATGAAATGACACATTGTTTTGACGATAAAGTAGCAACAAAGTTAGGAGTTGAAGCGGCATGC<br>GTATTGCACAACTTCGCTTTTTGGATAAACAAGAATATTGCCGATAACCACAATTATTTTGAGGGCAGATATTGGACTTATAACACAAGGGA<br>AGCGTTATCTAAACTATTCCCGTATATGAGTCAATCTAAGATATATAGAGTGATAGGAAAGTTGGAGGAAGAAGGCTATTTGTTGAAGGGGA<br>ATTTTAATAAATCGGGTATAGATAGAACAACGTGGTACGCATTAACAGATAAGTGTATAAAATTCCTTTTTGAGTGCGGATATACGCTTATA<br>GGCTATTCTGAGCCGATTTTGCAAAATTGCAAAATGCAAGTTGCAGAAATGAACAATGCAAGTTGCAGAAATGAACAAACAATACCAGATAG<br>TATATATACAGATAGTAATACTAAATCTACTAACGTAGATTATAGTATAGCCACGCGCGAAGAATCTGTTTTGTTCCCGGTTGAAAAGAAAC<br>CTTTAGCCTCAGAGATATTTGGCTTTACTGCAAAAGCCTTAGATGTGACTAAGAAAGTGATAGAGCGAACAGATAGTTTTTTCGATCAGCTA<br>ACATTCCCGTTCGAGTCGGAGGAATTTAAAAAAGCCTTTTATGTGCTAATGACTCAACCAAAGTGGCGGGTAAAGACTAAGACTCTAACAGC<br>TATGCAAGCAAACCTAAACGAGATTGCGCAATTTGAAGAAGGTTTTGCTATGCTATTGATAAATCAGAGCATATCTAAGGGATGGGCTTCAC<br>TGGTATACGAGTCAACGCTAAAACAGTATATGCAATGGCTACGGGAAAAGACGGGAGTCTCCGGAAATACACAGCCTGCAAACAATACTAAA<br>TCGTATTTTCAGAGTGACGAACAGCGCAGGATGTATCAGTCTTATTTAACGGAGGATTTTACATAGCATTTTAAGGCTTAAATTTCAATTTT<br>AATCACTAAGACAATAAAAGTATCATGTATTTGGAGAAAATCGAAAATTCGGGCGGAAAATTAGCAAAATACGAAGGTTGCGGATTGTTTAT<br>AGAGAAGAACCGAAAATTTTATGAAAGTGGCAACTTCGGACAGCTATCAAAAGTAGATCAAAAGATATTCCGTGATTCTACTTTGCTTTTGG<br>TGTCCGAATGTACAGACGAAAGAAAAAGAATAGATAATTTTTCTAAGGTTCTTAACGGAGTATGTTTAGAGACTGGTTTAAAAATGCCGGAT<br>GTCCGGGACGCAGGTAGTATATTTTATGCCGTTTGCGATGTGATAGATATGTATTTCTCTGATCTGTCGTTCAATGAAATTCGTTTAGCGTG<br>GAGGTTACTTGCTGTCGGGGAACTCGACCCGTTTTTGCCAAAAGACAGATATGGTAGTCCGGATAAAAATCATTATGGCTCTCTTTCGGTTG<br>ATTACATTTCAAAAGTCCTAAAGGCGTATAAGAAAAGAAAGGTTGAAACGATGGAACGAGTTTCTCAGATTATGCCGGACGAAAAGCCAAAG<br>CCGACACCCGAACAGGAAAAGATGTTTTTGAATTTGCAGGCATACAATTTTATTCTCGCCCTTTTGAAGTATAAATATTCGGGTCGTTTCCG<br>AATAGAGCGTGACAGGATAATAAACGAGTCTACATTTGCATACATGGAAAGATTGGGGTATGATATGTCGGTAGTACCGACGTTGGCGGACA<br>AGAAAGAAGCTTTGTTTCAATTTCAAGGTAGACCCGTAAATAGCTTTGCGCAAATTTTCGAAAAAGAGTGTATTTCGAGATTTGGGATAGAC<br>CACGAAGCAGTTTATTTTCGTGCGGTACTGATAGCCAAGAAAAGAAAGTTATTCCAGTATTGGGATGAAATGTTAGCTTTCTCAAATGAAGG<br>TGATAGATCAGAAGATAATATTTGGAAGTTGTACTACTACATTCAATAAAACCAAAAGTTATGAATAGAAGAAAAGTAAAAAAGAACGGTTA<br>TCGGATAAGGCTTACAAAGCCTTCCGATAAATTCGTTTATGTCTCTGACTCGTTAACATACGAAAGGAGAAAAAAGGAGGGAAAGAGATGTT<br>ATACTCTGTATTGCAAATATGCGTCTATTAACTATTTGT

Supplementary Figure S1. A PROMALS alignment of the amino acid sequences of the R subunit from *S. aureus* N315 with the R subunit of the Type IC enzyme, EcoR124I. The yellow shaded box indicates the motif essential for DNA cleavage in the nuclease domain. The green shaded boxes indicate the motifs in the motor domain essential for the ATPase-driven DNA translocation function associated with R subunits. The boxed sequences with the number of the RM_EB_X name underneath are the end points of the nuclease domain fragment fused to HsdM. “h” means alpha helix, “e” means beta strand, sequences in red or blue are the predicted or known secondary structures for the *S. aureus* sequence and the EcoR124I sequence respectively. The * indicates identical residues. (PROMALS: towards accurate multiple sequence alignments of distantly related proteins. Jimin Pei and Nick V. Grishin. *Bioinformatics* 2007; doi: 10.1093/bioinformatics/btm017).

```
Conservation:                       *                            ****  ** *    *   ***          * * *  *
EcoR124I_GAANNNNNNRTCG_10        1  MTHQTHTIAESNNFIVLDKYIKAEPTGDSYQSESDLERELIQDLRNQGYEFISVKSQSAMLANVREQLQN   70
HsdR_subunit__SauN315ORF1        1  M---------------------------AYQSEYALENEMMNQLEQLGYERVTIRDNKQLLDNFRTILNE   43
Consensus_ss:                                    eeeeeee            hhhhhhhhhhhhhh   ee     hhhhhhhhhhhhhh

Conservation:                            * *    * *  *            *    *            ***        ***    ** *
EcoR124I_GAANNNNNNRTCG_10       71  -----LNGVVFNDSEWRRFTEQYLDNPSDGILDKTRKIHIDYICDFIFDDERLENIYLIDKKNLMRNKVQ  135
HsdR_subunit__SauN315ORF1       44  RHADKLEGNPLTDKEFQRLLTMIDG---KSIFESARILRDKL--PLRRDDESEIYLSFLDKKSWCKNKFQ  108
Consensus_ss:                       hhhhhh      hhhhhhhhhhhh      hhhhhhhhhhhe  eeeee  eeeeeeeee       eee

Conservation:                          *         ******* ******* *** **  * *****  ** *        ** * * * ***
EcoR124I_GAANNNNNNRTCG_10      136  IIQQFEQAGSHANRYDVTILVNGLPLVQIELKKRGVAIREAFNQIHRYSKESFNSENSLFKYLQLFVISN  205
HsdR_subunit__SauN315ORF1      109  VTNQVSVEDTYKARYDVTILINGLPLVQVELKRRGIDINEAFNQVKRYRKQN---YTGLFRYIQMFIISN  175
Consensus_ss:                       eeeeeeee     eeeeeeee  eeeeeeee      hhhhhhhhhhhhhh     hhh   eeeeeeee
                                                                                     8

Conservation:                       *  **** *              *    *  *  *  *         *      *         *  ***
EcoR124I_GAANNNNNNRTCG_10      206  GTDTRYFANTTKRDKNSFDFTMNWAKSDNTLIKDLKDFTATCFQKHTLLNVLVNYSVFDSSQT-LLVMRP  274
HsdR_subunit__SauN315ORF1      176  GVETRYFSNNDSELLK--SHMFYWSDKQNNRINTLQSFAESFMRPCQLAKMISRYMIINETDRILMAMRP  243
Consensus_ss:                          eeeee                         hhhhhhhhhh hhhhhhhhhhh              h
                                           9                         4       5 3       6                 7

Conservation:                       **  * *                     ** ***************       * * ** * *******
EcoR124I_GAANNNNNNRTCG_10      275  YQIAATERILWKIKSSFTAKNWSKPESGGYIWHTTGSGKTLTSFKAARLATELDFIDKVFFVVDRKDLDY  344
HsdR_subunit__SauN315ORF1      244  YQVYAVEALIQQATE---------TGNNGYVWHTTGSGKTLTSFKASQILSQQDDIKKVIFLVDRKDLDS  304
Consensus_ss:                       hhhhhhhhhhhhhhhhhhhhh      eeeeee     hhhhhhhhhhhhhh      eeeee  hhhhh
                                                               2         10           11

Conservation:                       **  *   *    *    **  * * *       *******          *        **** *****
EcoR124I_GAANNNNNNRTCG_10      345  QTMKEYQRFSPDSVNGSENTAGLKRNLDKDDNKIIVTTIQKLNNLMKAESDLPVY--NQQVVFIFDECHR  412
HsdR_subunit__SauN315ORF1      305  QTEEEFNKFAKGAVDKTFNTSQLVRQLNDKSLPLIVTTIQKMAKAIQGNAHLLEQYKTNKVVFIIDECHR  374
Consensus_ss:                       hhhhhhhhh     eeeee hhhhhhhh     eeeeehhhhhhhhhhhhhhhhh     eeeeee hhh

Conservation:                       ****      *  **    ******* ****      ***  *** ** * * *** *  ** * ***
EcoR124I_GAANNNNNNRTCG_10      413  SQFGEAQKNLKKKFKRYYQFGFTGTPIFPENALG-SETTASVFGRELHSYVITDAIRDEKVLKFKVDYND  481
HsdR_subunit__SauN315ORF1      375  SQFGDMHRLVKQHFKNAQYFGFTGTPRFPENSSQDGRTTADIFGRCLHTYLIRDAIHDGNVLGFSVDYIN  444
Consensus_ss:                         hhhhhhhhhhh    eeeeee       hhhhh   hhhh         hhhhhh      eeeeeee

Conservation:                                   *     *       *   *    *  * **    *               * *  *
EcoR124I_GAANNNNNNRTCG_10      482  VRPQFKSLETETDEKKLSAAENQQAFLHPMRIQEITQYILNNFRQKTHRTFPGSKGFNAMLAVSSVDAAK  551
HsdR_subunit__SauN315ORF1      445  TFKNKAL--KAEDNSMVEAIDTEEVWLADKRVELVTRHIINNHDKYTR-----NRQYSSIFTVQSIHALI  507
Consensus_ss:                          hhhhhhhhhhhhhhhhhhhhhhh  hhhhhhhhhhhhhhhhhhhhhh        eeeee  hhhhh

Conservation:                        ** *****               ** ** *  **                       * *   *  **
EcoR124I_GAANNNNNNRTCG_10      552  AYYATFKRLQEEAANKSATYKPLRIATIFSFAANEEQNAIGEISDETFDTSAMDSSAKEFLDAAIREYNS  621
HsdR_subunit__SauN315ORF1      508  KYYETFKRLNKKLEQPL------TIAGIFTFKPNEDDRDGEVPY-----------HSREKLEIMISDYNK  560
Consensus_ss:                       hhhhhhhhhhhhhhh        eeeeeeee   hhhhhhhhhhh          hhhhhhhhhhhhhhh

Conservation:                        * ******     *          **   ** **** *******   **** ***** ** * ** ***
EcoR124I_GAANNNNNNRTCG_10      622  HFKTNFSTD--SNGFQNYYRDLAQRVKNQDIDLLIVVGMFLTGFDAPTLNTLFVDKNLRYHGLMQAFSRT  689
HsdR_subunit__SauN315ORF1      561  KFETNFSTDTTNEYFNHISKNVKKGVKDSKIDILIVVNMFLTGFDSKVLNTLYVDKNLMYHDLIQAYSRT  630
Consensus_ss:                       hh             hhhhhhhhhh     eeeeeee  hhh      eeeeee     hhhhhhhhhhh

Conservation:                       **     * ** **  ***   * **   *   *     *  ** **   *  **
EcoR124I_GAANNNNNNRTCG_10      690  NRIYDATKTFGNIVTFRDLERSTIDAITLFGDKNTKNVVLEKSYTEYMEGFTDAATGEAKRGFMTVVSEL  759
HsdR_subunit__SauN315ORF1      631  NRVEKESKPFGKIVNYRDLKKETDDALRVFSQTNDTDTILMRSYEEYKKEFMDAYRE---------LKMI  691
Consensus_ss:                                eeeeee    hhhhhhhhhhh        hhhhhhhhhhhhhhhhhhhhhhhhhhhhhhhh

Conservation:                                *  * * * **            *   ***         *
EcoR124I_GAANNNNNNRTCG_10      760  EQRFPDPTSIESEKEKKDFVKLFGEYLRAENILQNYDEFATLKALQQIDLSDPVAVEKFKAEHYVDDEKF  829
HsdR_subunit__SauN315ORF1      692  VPTPHMVDDIQDEEELKRFVEAYRLLAKIILRLKAFDEFEFTIDEIGMDEQENED---------------  746
Consensus_ss:                       hhhhhhhhh   hhhhhhhhhhhhhhhhhhhhhhhhhhhhhhhhhhhhhhhhhhhhhhhhhhhhhhhhhh
```

```
Conservation:                                       * * *    *       *           *  **        **  **
EcoR124I_GAANNNNNNRTCG_10     830  AELQTIRLPADRKIQDYRSAYNDIRDWQRREKEAEKKEKSTTDWDDVVFEVDLLKSQEINLDYILGLIFE  899
HsdR_subunit__SauN315ORF1     747  ----------------YKSKYLAVYDQVK---RATAEKNKVSILNDIDFEIEMMRNDTINVNYIMNILRQ  797
Consensus_ss:                      hhhhhh    hhhhhhhhhhhhhhhhhhhhhhhhhhh  hhhhhhhhhhhhhhhhhhh  hhhhhhhhhh

Conservation:                           *       *  * *         * *  *   **       *     *      *       *
EcoR124I_GAANNNNNNRTCG_10     900  HNRQNKGKGEMIEEVKRLIRS---SLGNRAKEGLVVDFIQQTNLDDLPDKASIIDAFFTFAQREQQREAE  966
HsdR_subunit__SauN315ORF1     798  IDLEDKAEQRRNQEQIRRILDHADDPTLRLKRDLIREFIDNVVP-SLNKDDDIDQEYVNFESIKKEAEFK  866
Consensus_ss:                      hhh  hhhhhhhhhhhhhhhhh   hhhhhhhhhhhhhhhhhh      hhhhhhhhhhhhhhhhhhhhh

Conservation:                          *    * * *                           **         *
EcoR124I_GAANNNNNNRTCG_10     967  ALIKEENLNEDAAKRYIRTSLKREYATENGTELNETLPKLSPLNPQYKTKKQAVFRKSSRLLRSLKA 1033
HsdR_subunit__SauN315ORF1     867  GFAGERSIDEQALKTISNDYQYSGVVNP---HHLKKMIGDLPLKEK-RKARKAIESFVAETTEKYGV  929
Consensus_ss:                      hhhhhh   hhhhhhhhhhhhh hhhhhhhhhhhhhhhhh    hhhhhhhhhhhhhhhhhhhhh
```

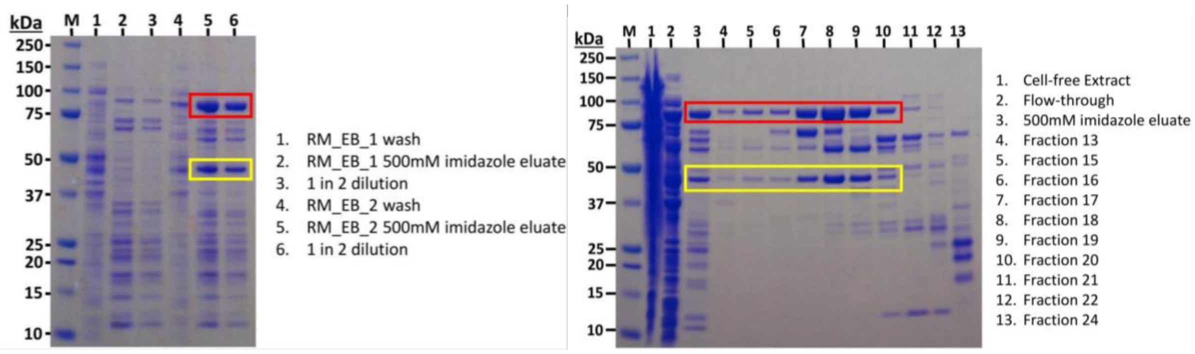

Figure S2. Nickel affinity purification of the RM_EB_2 RM fusion protein (left panel). Size exclusion purification of the RM_EB_2 RM fusion protein collected from the nickel affinity column (right panel). The red rectangle highlights the RM fusion protein, whilst the yellow rectangle indicates the HsdS subunit. M indicates the marker lane.

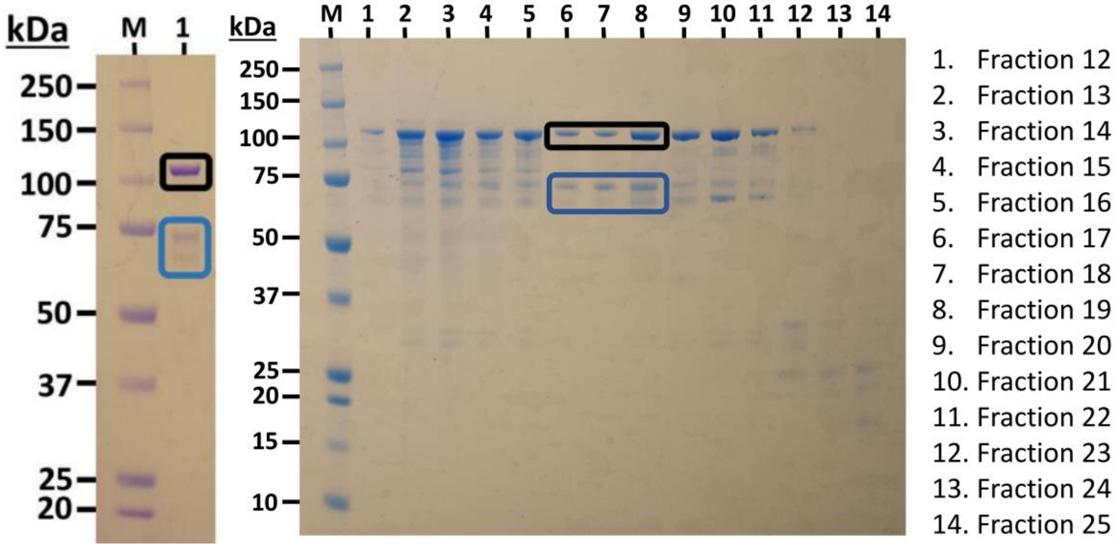

Figure S3. SDS-PAGE analysis of the purified MS_{fus} protein (black box, left panel). The gel also shows a couple of contaminants (light blue box), the smaller of which was identified as a truncated form of the fusion. M is a marker lane. SDS-PAGE analysis of samples from the Sephacryl S200 size exclusion purification of the MS_{fus} protein (right panel). The bands highlighted by the black box are the target protein, whilst the blue box highlights the co-eluting fragments. M – marker, lanes 1 to 14 are fractions 12 to 25 from the elution.

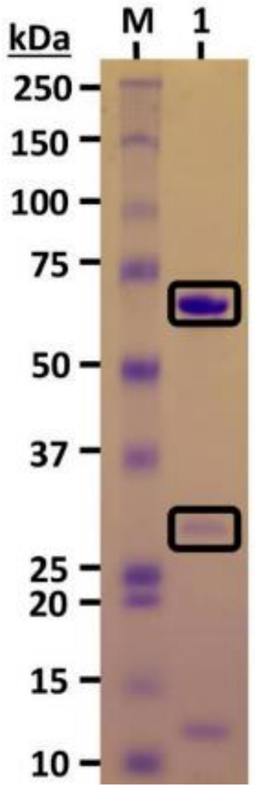

Figure S4. SDS-PAGE analysis of the purified M½S MTase. The black boxes highlight the HsdM subunit (upper) and the half HsdS protein (lower). M is a marker lane.

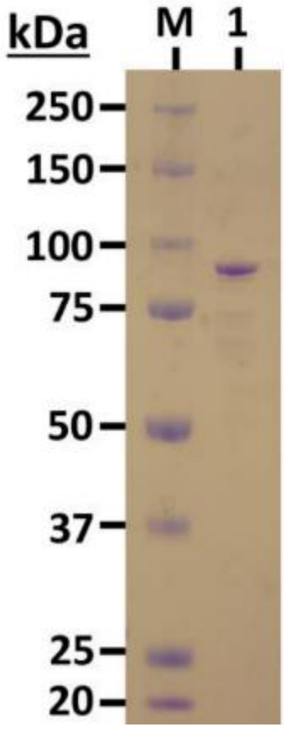

Figure S5. SDS-PAGE analysis of the purified M½S_{fus} MTase fusion. M is a marker lane.

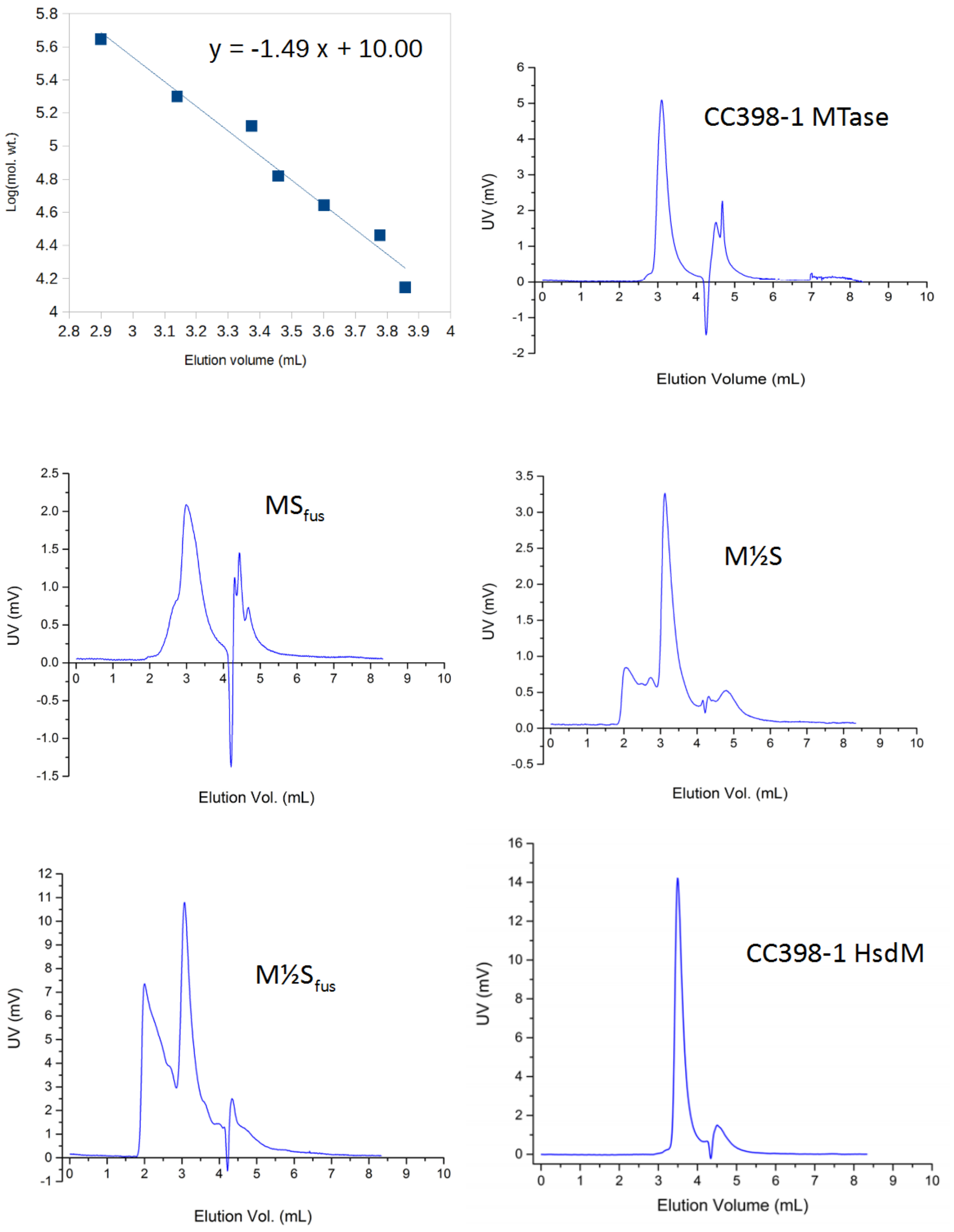

Figure S6. Analytical size exclusion analyses of wild type MTase (CC398-1), the MTase derivatives and the purified HsdM subunit from the CC398-1 RM system (CC398-1 HsdM). In the top left panel from left to right the calibration proteins are apoferritin, β-amylase, bovine serum albumin (dimer), bovine serum albumin (monomer), ovalbumin, carbonic anhydrase, α-lactalbumin. HPLC gel filtration used a Biosep-SEC-S3000 (30 cm x 0.46 cm) gel filtration column (Phenomenex); 50 μL samples in 20 mM Tris, 20 mM MES, 0.2 M NaCl, 10 mM $MgCl_2$, 7 mM β-mercaptoethanol, and 0.1 mM EDTA, pH 6.5, were injected onto the column. The flow rate was 0.5 mL/min, and detection was at 280 nm. The baseline oscillation at ~4.3 minutes in the elution profiles is due to the elution of the small amount of glycerol present in each injected protein sample. Material eluting prior to the main peak at ~3 minutes is due to protein aggregation in the injected sample.

Table S5. Molecular weights of main elution peaks from analytical size exclusion chromatography shown in Figure S6.

Species	Assumed stoichiometry	Expected Mol. Wt. (kDa) for assumed stoichiometry	Measured Mol. Wt. (kDa)
MTase	M_2S_1	166	241
M½S	$(M½S)_2$	172	225
MS_{fus}	$(MS_{fus})_2$	213	325
$M½S_{fus}$	$(M½S_{fus})_2$	173	251
HsdM	$(HsdM)_1$	59	61

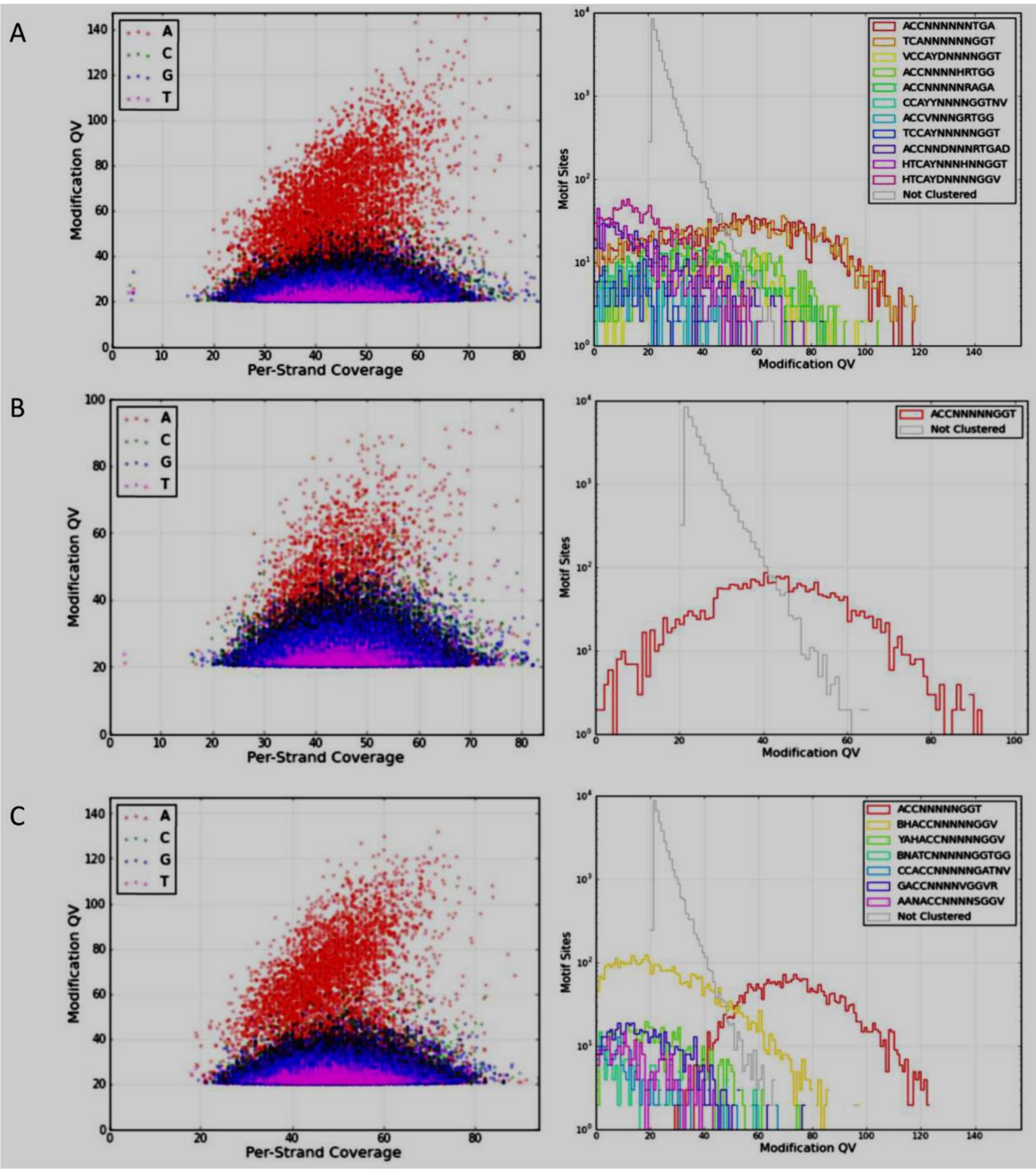

Figure S7. Results from SMRT Sequencing analysis of *E. coli* ER2796 genomic DNA, after modification by the MS$_{fus}$ MTase (A), M½S MTase (B), and the M½S$_{fus}$ MTase (C). Graphs on the left-hand side are scatter plots, which show the frequency of detection of specific base modification. For each of the MTases there is a higher frequency of points corresponding to N6-methylated adenine (red) with high quality value (QV). This indicates that the MTases are adenine MTases. The graphs on the right-hand side show the frequency of detection of methylated DNA sequence motifs. Again, in each case the red line shows the methylated motif detected at the highest frequency. In the case of the MS$_{fus}$ MTase (A), this is the wild-type motif, ACCN$_5$RTGA and its complement. For the other two MTases, M½S MTase (B) and the M½S$_{fus}$ MTase (C), this is the palindromic sequence, ACCN$_5$GGT.

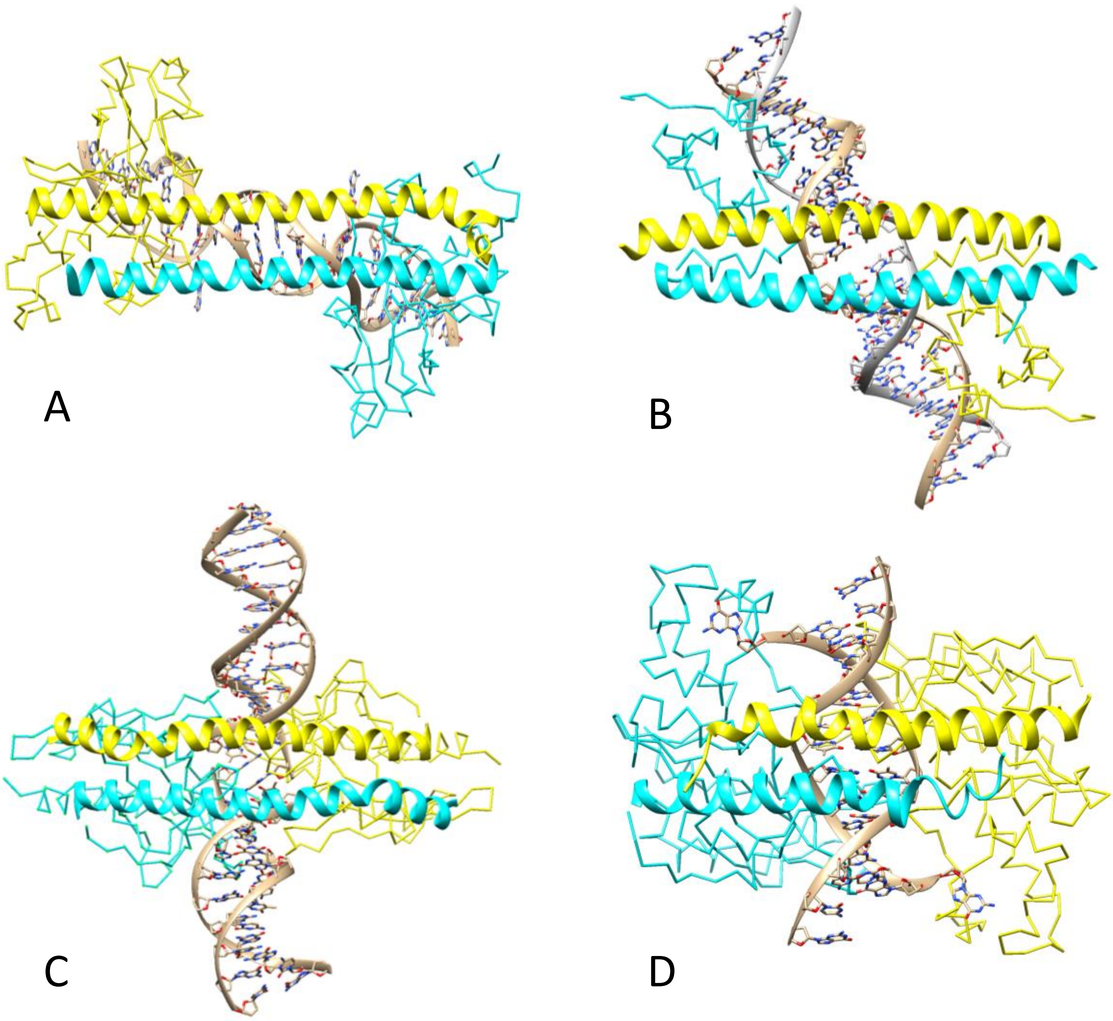

Supplementary figure S8. The angle between the two α helices (yellow and cyan, one from each protein subunit) and the helical axis of the bound DNA increases as the two parts of the target sequences (and the methylation/cleavage sites) become closer. (A) EcoKI HsdS shown bound to AACNNNNNNGTGC (PDB: 2Y7H, underlined bases are locations for methylation and 8 bp apart, amino acids 1-430 shown). (B) BmrR transcription factor bound to operator DNA containing the internal repeat TCCGGANNNNCCGGA (PDB:3D71, amino acids 1-117 shown, DNA sequences only 4 bp apart). (C) SwaI REase bound to DNA containing target ATTT^AAAT (PDB: 5TH3, blunt end cleavage marked by ^, palindromic target has no non-specific bp). (D) HincII REase bound to DNA containing target GTY^RAC (PDB: 2GIE, blunt end cleavage marked by ^, palindromic target has no non-specific bp).
